# Systematic Review to Inform a World Health Organization (WHO) Clinical Practice Guideline: Benefits and Harms of Transcutaneous Electrical Nerve Stimulation (TENS) for Chronic Primary Low Back Pain in Adults

**DOI:** 10.1007/s10926-023-10121-7

**Published:** 2023-11-22

**Authors:** Leslie Verville, Cesar A. Hincapié, Danielle Southerst, Hainan Yu, André Bussières, Douglas P. Gross, Paulo Pereira, Silvano Mior, Andrea C. Tricco, Christine Cedraschi, Ginny Brunton, Margareta Nordin, Gaelan Connell, Heather M. Shearer, Jessica J. Wong, Léonie Hofstetter, Andrew Romanelli, Brett Guist, Daphne To, Kent Stuber, Sophia da Silva-Oolup, Maja Stupar, Danny Myrtos, Joyce G.B. Lee, Astrid DeSouza, Javier Muñoz Laguna, Kent Murnaghan, Carol Cancelliere

**Affiliations:** 1 Institute for Disability and Rehabilitation Research and Faculty of Health Sciences, Ontario Tech University, Oshawa, Canada; 2 EBPI-UWZH Musculoskeletal Epidemiology Research Group, University of Zurich and Balgrist University Hospital, Zurich, Switzerland; 3 Epidemiology, Biostatistics and Prevention Institute (EBPI), University of Zurich, Zurich, Switzerland; 4 University Spine Centre Zurich (UWZH), Balgrist University Hospital and University of Zurich, Zurich, Switzerland; 5 Département chiropratique, Université du Québec à Trois-Rivières, Trois-Rivières (Québec), Québec, Canada; 6 School of Physical and Occupational Therapy, Faculty of Medicine and Health Sciences, McGill University, Québec, Canada; 7 Department of Physical Therapy, University of Alberta, Edmonton, Canada; 8 Department of Neurosurgery, Centro Hospitalar Universitário São João, Porto, Portugal; 9 Faculty of Medicine, University of Porto, Porto, Portugal; 10 Department of Research and Innovation, Canadian Memorial Chiropractic College, Toronto, Canada; 11 Li Ka Shing Knowledge Institute, St. Michael’s Hospital, Unity Health Toronto, Toronto, Canada; 12 Epidemiology Division and Institute for Health Policy, Management, and Evaluation, Dalla Lana School of Public Health, University of Toronto, Toronto, Canada; 13 Queen’s Collaboration for Health Care Quality Joanna Briggs Institute Centre of Excellence, Queen’s University, Kingston, Canada; 14 Division of General Medical Rehabilitation, Geneva University and University Hospitals, Geneva, Switzerland; 15 Division of Clinical Pharmacology and Toxicology, Multidisciplinary Pain Centre, Geneva University Hospitals, Geneva, Switzerland; 16 EPPI-Centre, UCL Institute of Education, University College London, England, United Kingdom; 17 Department of Health Research Methods, Evidence and Impact, Faculty of Health Sciences, McMaster University, Hamilton, Canada; 18 Departments of Orthopedic Surgery and Environmental Medicine, NYU Grossman School of Medicine, New York University, New York, United States; 19 Bloorview Research Institute, Holland Bloorview Kids Rehabilitation Hospital, Toronto, Canada; 20 Department of Clinical Education, Canadian Memorial Chiropractic College, Toronto, Canada; 21 Department of Undergraduate Education, Canadian Memorial Chiropractic College, Toronto, Canada; 22 Parker University Research Center, Dallas, United States; 23 Department of Graduate Education, Canadian Memorial Chiropractic College, Toronto, Canada; 24 Library and Information Services, Canadian Memorial Chiropractic College, Toronto, Canada

**Keywords:** Low back pain, Systematic review, Meta-analysis, Transcutaneous electrical nerve stimulation

## Abstract

**Purpose:**

To evaluate benefits and harms of transcutaneous electrical nerve stimulation (TENS) for chronic primary low back pain (CPLBP) in adults to inform a World Health Organization (WHO) standard clinical guideline.

**Methods:**

We searched for randomized controlled trials (RCTs) from various electronic databases from July 1, 2007 to March 9, 2022. Eligible RCTs targeted TENS compared to placebo/sham, usual care, no intervention, or interventions with isolated TENS effects (i.e., combined TENS with treatment B versus treatment B alone) in adults with CPLBP. We extracted outcomes requested by the WHO Guideline Development Group, appraised the risk of bias, conducted meta-analyses where appropriate, and graded the certainty of evidence using GRADE.

**Results:**

Seventeen RCTs (adults, n = 1027; adults ≥ 60 years, n = 28) out of 2010 records and 89 full text RCTs screened were included. The evidence suggested that TENS resulted in a marginal reduction in pain compared to sham (9 RCTs) in the immediate term (2 weeks) (mean difference (MD) = -0.90, 95% confidence interval  -1.54 to -0.26), and a reduction in pain catastrophizing in the short term (3 months) with TENS versus no intervention or interventions with TENS specific effects (1 RCT) (MD = -11.20, 95% CI -17.88 to -3.52). For other outcomes, little or no difference was found between TENS and the comparison interventions. The certainty of the evidence for all outcomes was very low.

**Conclusions:**

Based on very low certainty evidence, TENS resulted in brief and marginal reductions in pain (not deemed clinically important) and a short-term reduction in pain catastrophizing in adults with CPLBP, while little to no differences were found for other outcomes.

**Supplementary Information:**

The online version contains supplementary material available at 10.1007/s10926-023-10121-7.

## Introduction

Electrical stimulation therapies are therapeutic adjuncts used in the management of chronic pain conditions such as osteoarthritis, fibromyalgia, and chronic primary low back pain (CPLBP) [1–3]. They include a range of non-invasive peripheral stimulation techniques to relieve pain. Among these therapies, transcutaneous electrical nerve stimulation (TENS) and interferential therapy are the most used low volatage electrical stimulation therapies [1, 2].

Both are reported to have similar mechanisms of action, namely acting through segmental inhibition or activation of descending pain-inhibitory systems [1]. TENS units are widely available and accessible globally [4]. A TENS unit is a battery-powered device that can be self-administered and delivers electrical impulses through electrodes placed on the intact skin surface near the source of maximal pain. Interferential therapy involves a different form of electrical stimulation than TENS, and treatment is administered using two pairs of electrodes usually in a clinical setting. Compared to interferential therapy, TENS is used more frequently as a self-delivered intervention given that it is inexpensive and easily accessible.

In 2008, Khadilkar and colleagues published a Cochrane systematic review to assess the effectiveness of TENS versus placebo for the management of CPLBP (4 randomized controlled trials [RCTs], 585 patients) [4]. Their outcomes of interest were pain, functional status, generic health status, work disability, participant satisfaction, treatment side effects, physical examination measures (e.g., range of motion), medication use, and use of medical services. The authors concluded that the evidence did not support the use of TENS for the routine management of CPLBP. Similarly, Resende et al. (2018) found low-quality evidence that TENS did not improve function immediately after therapy when compared to placebo [5]. However, little is known about the effects of TENS versus other interventions and benefits and harms in people with CPLBP – pain between the lower costal margin and the gluteal fold with no specific underlying cause of more than three months duration. Therefore, to develop clinical practice guideline recommendations for the management of CPLBP in adults, the World Health Organization (WHO) commissioned the current systematic review to update the evidence and expand the aims of the Cochrane review [4] by assessing additional comparators (e.g., no intervention, usual care), important outcomes (e.g., psychological functioning, social participation including work), and conducting additional subgroup analyses (e.g., gender/sex, race/ethnicity).

The objectives of this systematic review of RCTs were to determine: (1) the benefits and harms (as reported in RCTs) of TENS compared to placebo/sham, usual care, or no intervention for the management of CPLBP in adults, including older adults (aged ≥ 60 years); and (2) whether the benefits and harms of TENS vary by age, gender/sex, presence of leg pain, race/ethnicity, or national economic development of the countries where the RCTs were conducted.

## Methods

This systematic review was conducted as part of a series of reviews to inform the WHO guideline on the management of CPLBP in adults. The development of this guideline was ongoing at the time of submission of this manuscript. The methods are detailed in the methodology article of this series [3, 6].

Briefly, we updated and expanded the scope of the previously published high-quality Cochrane systematic review by Khadilkar et al. (2008) [4]. We registered our review protocol with Prospero (CRD42022314817) on 7 March 2022. We searched MEDLINE (Ovid), CINAHL (EBSCO), Embase (Ovid), Cochrane Central Register of Controlled Trials (Wiley), PEDRO, and the WHO International Clinical Trials Registry Platform (ICTRP) from the period of 1 July 2007 (end date of previous Cochrane review) to 9 March 2022 (see Online Resource 1). We also searched the reference lists of systematic reviews and included RCTs.

We included RCTs that compared TENS to placebo/sham, usual care, and no intervention (including comparison interventions where the attributable effect of TENS could be isolated, e.g., TENS + medication vs. same medication alone) in adults (aged ≥ 20 years) with CPLBP. TENS interventions could be applied using device settings of any of the stimulation parameters including pulse intensity, frequency, duration, and type (burst or continuous). RCTs of electrical stimulation administered percutaneously using needles were excluded. In addition to the main critical outcomes requested by the WHO Guideline Development Group (GDG) and assessed for all reviews in this series (pain, function, health-related quality of life, harms, psychological functioning, and social participation including work), we also assessed additional critical outcomes requested by the WHO GDG for this review – the change in use of medications and falls in older adults (aged ≥ 60 years). We reported outcomes based on post-intervention follow-up intervals including: (1) immediate term (closest to 2 weeks after the intervention period); (2) short term (closest to 3 months after the intervention period); (3) intermediate term (closest to 6 months after the intervention period); (4) long term (closest to 12 months after the intervention period); and (5) extra-long term (more than 12 months after the intervention period).

We assessed between-group differences to determine the magnitude of the effect of an intervention and to assess its effectiveness [7, 8] (details in the methodology article in this series) [6]. Briefly, we considered a mean difference (MD) of ≥ 10% of the scale range (e.g., MD = 1 on visual analogue scale 0 to 10) or ≥ 10% difference in risk for dichotomous outcomes to be a minimally important difference (MID) [9, 10]. If the standardized mean difference (SMD) was calculated, SMD ≥ 0.2 was considered a MID [11].

Pairs of reviewers independently screened studies for eligibility, and critically appraised risk of bias using the Cochrane ROB 1 tool [12], modified from the Cochrane Back and Neck Methods Guidelines [13]. One reviewer extracted data for all included RCTs, which was then verified by a second reviewer. Any disagreements were resolved by consensus between paired reviewers or with a third reviewer when necessary. Forms and guidance for screening, risk of bias assessment, and data extraction were adapted from those used by Hayden et al. in the conduct of the ‘exercise for chronic low back pain’ collaborative review, in which members of our team participated [14]. The forms were modified and completed using a web-based electronic systematic review software DistillerSR Inc. [15].

In addition to the main sub-group analyses conducted for all reviews in this series (age, gender/ sex, presence of leg pain, race/ethnicity, national economic development of country where RCT was conducted), we conducted the following pre-specified sub-group and sensitivity analyses: number of treatment sessions (i.e., ≥ 10 sessions vs. <10 sessions) and removal of RCTs rated as high risk of bias.

We conducted random-effects meta-analyses and narrative synthesis where meta-analysis was not appropriate [16], and graded the certainty of evidence using Grading of Recommendations Assessment, Development and Evaluation (GRADE) [17]. The comparisons no intervention and interventions where the specific effects of TENS could be isolated were combined in meta-analyses. Meta-analyses were conducted using R [18, 19], and GRADE Evidence Profiles and GRADE Summary of Findings tables were developed using GRADEpro software [20].

## Results

**Fig. 1:**
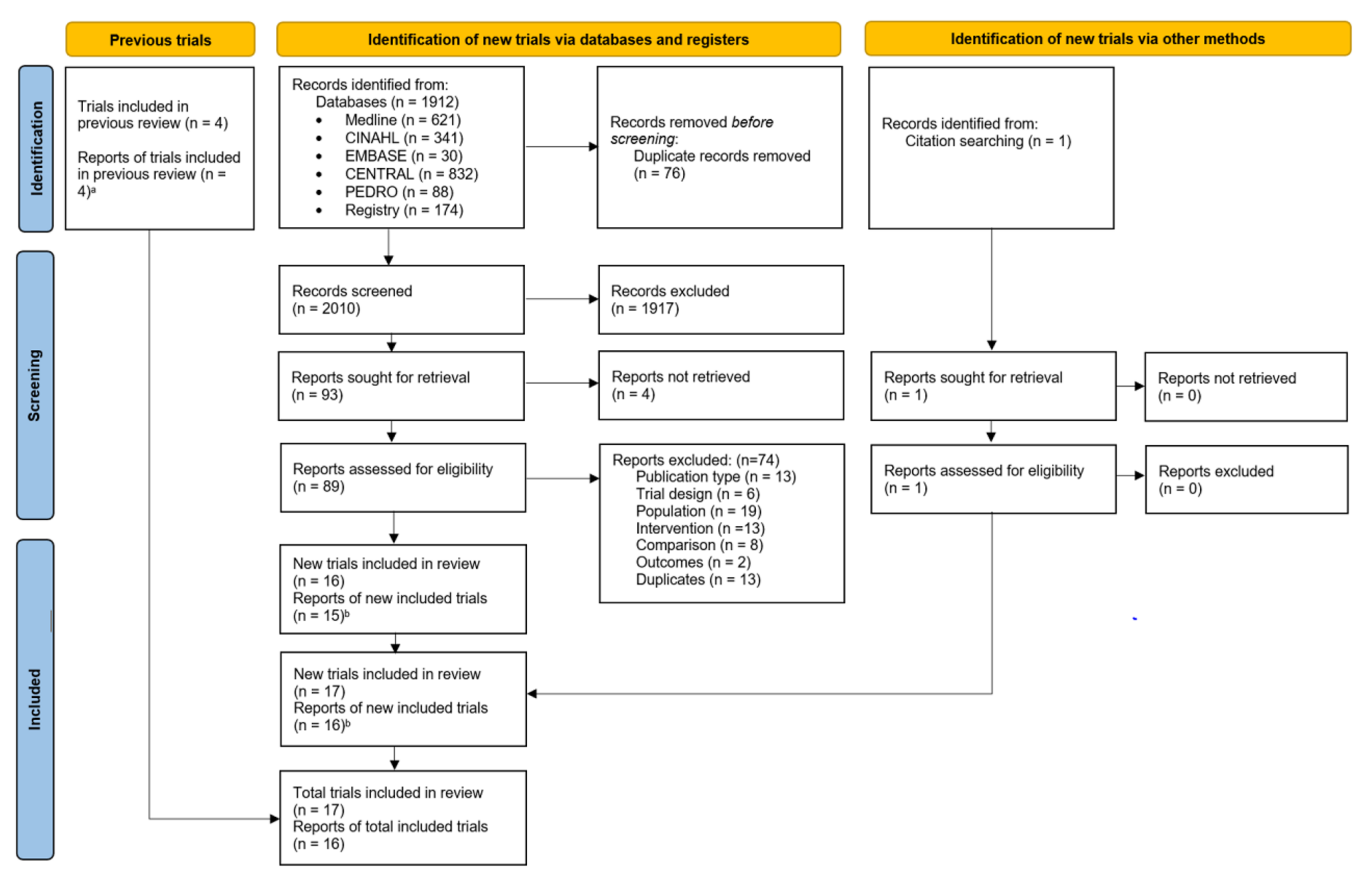
Flow diagram of literature search ^a^4 RCTs from previous review were also identified from current search; thus, they did not add to the total ^b^1 report contained 2 RCTs [2]

We screened 2010 records and 89 full-text reports (Fig. 1). We identified five unpublished RCTs in the WHO ICTRP, of which we contacted the authors with contact information listed (four of the five). One author responded to inform us that the RCT was ongoing, and therefore was not included in our review. Thus, none of the five unpublished RCTs identified in the WHO ICTRP were included (because the other three authors did not reply). We included 17 published RCTs (16 reports) [2, 21–35] with a total of 1027 adults (ranging from 11 to 134 adults per RCT) from predominantly healthcare settings (see Online Resources 2, 3). The RCTs were conducted in high-income economies [36]: Canada (1 RCT) [23], Greece (1 RCT) [31], Japan (1 RCT) [2], and the United States (3 RCTs) [22, 29, 32]; upper-middle income economies: Brazil (3 RCTs) [24–26], China (1 RCT) [23], and Turkey (3 RCTs) [30, 34, 35]; and lower-middle income economies: Egypt (1 RCT) [27], Iran (1 RCT) [21], and Nigeria (2 RCTs) [28, 33]. The mean age ranged from 22 to 64 years; two RCTs (both included in one report) assessed older adults (n = 28) [2]. The percentage of females within the RCTs ranged from 13 to 100%. In eight RCTs, adults had CPLBP without leg pain [21, 23, 24, 27, 28, 31, 32, 35], in four RCTs (three reports) adults had CPLBP either with or without leg pain (radicular or non-radicular) [2, 29, 30], in a single RCT, adults had CPLBP with radiculopathy [22], and presence of leg pain was not reported in four RCTs [25, 26, 33, 34]. Where reported by authors, CPLBP duration ranged from 31 weeks to 13 years.

The TENS interventions in the included RCTs involved electrode placement over the paravertebral lumbosacral area and sometimes to the affected leg, using conventional continuous or burst pulse types. TENS was compared to either sham TENS (11 RCTs) [2, 22–24, 26, 28, 30–32, 34, 35], no intervention (1 RCT) [29], or interventions where the specific effects of TENS were isolated (9 RCTs) [2, 21, 24, 25, 27, 30–33] (Table 1). Sham TENS used similar electrode placements as the intervention groups, except the current was switched off. We did not find any RCTs comparing TENS to usual care. The outcomes were assessed in the immediate term (closest to 2 weeks after the intervention period) (14 RCTs in 13 reports) [2, 21–28, 30, 32–34], or short term (closest to 3 months after the intervention period) (1 RCT) [29], or both (2 RCTs) [31, 35]. None of the included RCTs assessed outcomes in the intermediate (closest to 6 months after the intervention period), long (closest to 12 months after the intervention period) or extra long (> 12 months after the intervention period) term. The RCTs were rated as overall high (14, 82%), or unclear (3, 18%) risk of bias (Online Resource 4). The agreement on overall ROB ratings was high (weighted overall kappa score 0.95).

**Table 1:** Number of included RCTs by comparison and outcome

Outcome	Follow-up				
	Immediate (2 weeks)	Short (3 months)	Intermediate (6 months)	Long (12 months)	Extra-long (> 12 months)
**TENS versus sham**					
Pain	10^a^	2	-	-	-
Function	4	2	-	-	-
HRQoL	2	-	-	-	-
Fear avoidance	-	-	-	-	-
Catastrophizing	-	-	-	-	-
Depression	-	1	-	-	-
Anxiety	-	-	-	-	-
Self-efficacy	-	-	-	-	-
Social participation	-	-	-	-	-
Medication use	-	-	-	-	-
Falls	-	-	-	-	-
Harms	1	-	-	-	-
**TENS versus no intervention or interventions where effects of TENS were isolated**					
Pain	8	2	-	-	-
Function	6	2	-	-	-
HRQoL	1	-	-	-	-
Fear avoidance	-	-	-	-	-
Catastrophizing	-	1	-	-	-
Depression	-	1	-	-	-
Anxiety	-	-	-	-	-
Self-efficacy	-	-	-	-	-
Social participation	-	-	-	-	-
Medication use	-	-	-	-	-
Falls	-	-	-	-	-
Harms	1	-	-	-	-
**TENS versus usual care**					
All outcomes	-	-	-	-	-

HRQoL: health-related quality of life.

^a^2 RCTs included older adults (aged ≥ 60 years)

### Certainty of Evidence

The certainty of the evidence for all outcomes was very low, and was downgraded due to risk of bias, inconsistency, indirectness, and imprecision of the effect estimates (see Online Resources 5, 6 and 7).

### TENS Versus Sham

#### All Adults

Due to very low certainty evidence, it is uncertain whether TENS reduces ***pain ****(scale 0 to 10, 0 = no pain)* in the immediate term (9 RCTs; mean difference (MD) = -0.90, 95% confidence interval (CI) -1.54 to -0.26) (see Online Resource 7, plot 1.1.1) [2, 23, 24, 26, 28, 30, 31, 34, 35]. The effect estimate did not reach the threshold for what we considered to be a minimally important between-group difference (MD = -1) [6]. It is uncertain whether TENS makes little or no difference to pain in the short term (2 RCTs; MD = -0.40, 95% CI -2.21 to 1.41) (plot 1.1.2) [31, 35].

**Fig. 2:**
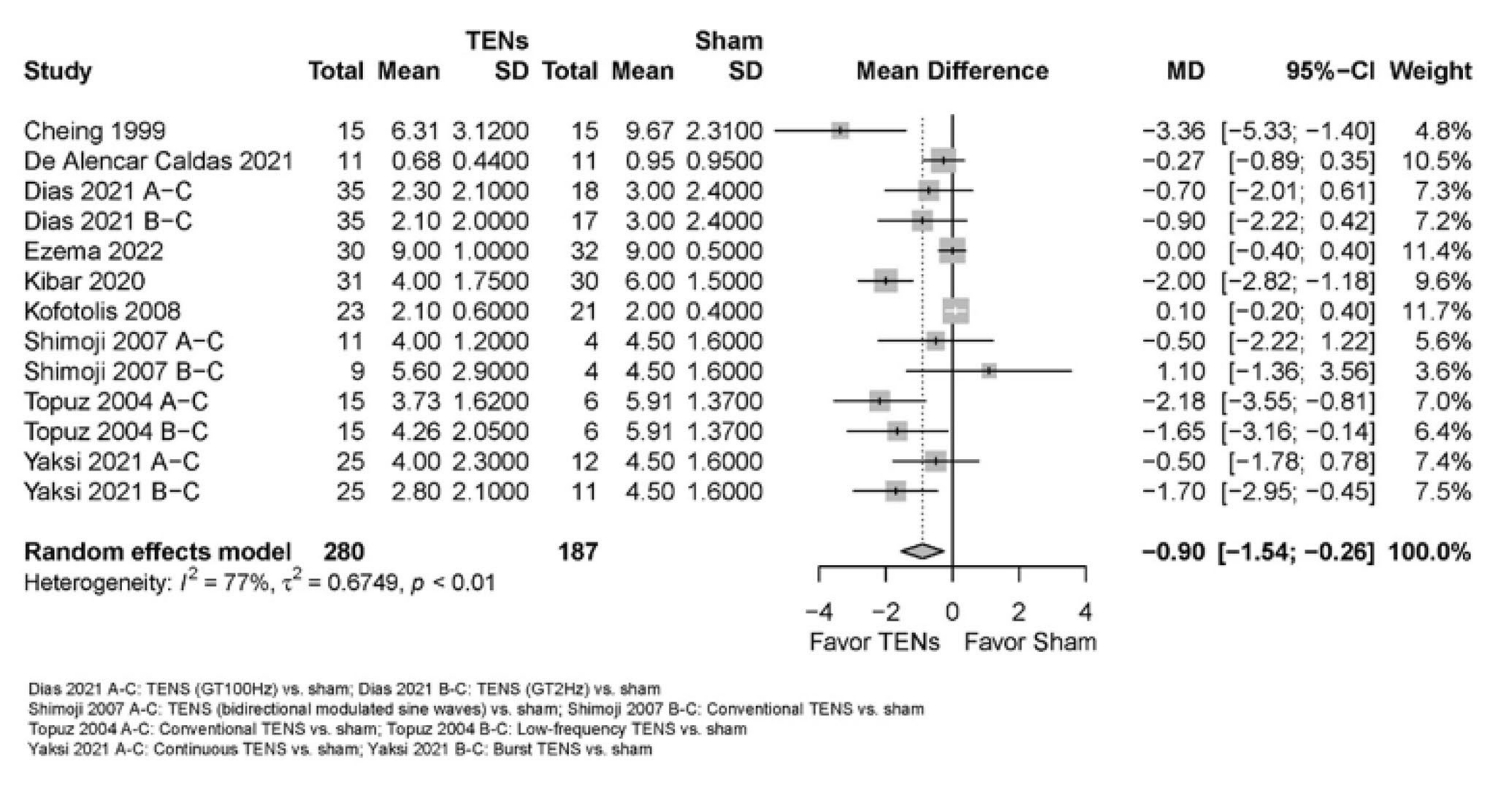
TENS versus sham for pain in the immediate term (closest to 2 weeks); scale range is 0 to 10

Due to very low certainty evidence, it is uncertain whether TENS makes little or no difference to ***function*** in the immediate (4 RCTs; standardized mean difference (SMD) = -0.96, 95% CI -3.20 to 1.28; *benefit indicated by lower values*) (plot 1.2.1) [24, 30, 31, 32], or short term (2 RCTs; MD = -0.24, 95% CI -4.30 to 3.81; *scale 0 to 50, 0 = no disability*) (plot 1.2.2) [31, 35]. It is uncertain whether TENS makes little or no difference to ***health-related quality of life ****(scale 0 to 100, 0 = poor quality of life; PCS: physical component summary, MCS: mental component summary)* in the immediate term (2 RCTs; ***PCS***: MD = 3.21, 95% CI -21.17 to 27.59; plot 1.3.1; ***MCS***: MD = 3.57, 95% CI -30.06 to 37.20; plot 1.4.1) [24, 34]. It is uncertain whether TENS makes little or no difference to ***depression ****(scale 0 to 63, 0 = no depression)* in the short term (1 RCT; MD = 3.04, 95% CI -19.15 to 25.22) (plot 1.5.1) [35]. It is uncertain whether TENS makes little or no difference to ***adverse events/harms*** (1 RCT) (no plot, narrative synthesis) [35]. None of the other RCTs assessed adverse events.

### Older Adults (aged ≥ 60 Years)

Due to very low certainty evidence, in older adults, it is uncertain whether TENS makes little or no difference to ***pain ****(scale 0 to 10, 0 = no pain)* in the immediate term (1 RCT; MD = 0.13, 95% CI -9.80 to 10.06) (plot 1.6.1.1) [2].

### TENS Versus no Intervention or Interventions where the Attributable Effect of TENS could be Isolated

#### All Adults

Due to very low certainty evidence, it is uncertain whether TENS makes little or no difference to ***pain ****(scale 0 to 10, 0 = no pain)* in the immediate (8 RCTs; MD = -0.19, 95% CI -0.51 to 0.14) (see Online Resource 7, plot 2.1.1) [2, 24, 25, 27, 30–33], or short term (2 RCTs; MD = -0.98, 95% CI -16.83 to 14.88) (plot 2.1.2) [29, 31].

**Fig. 3:**
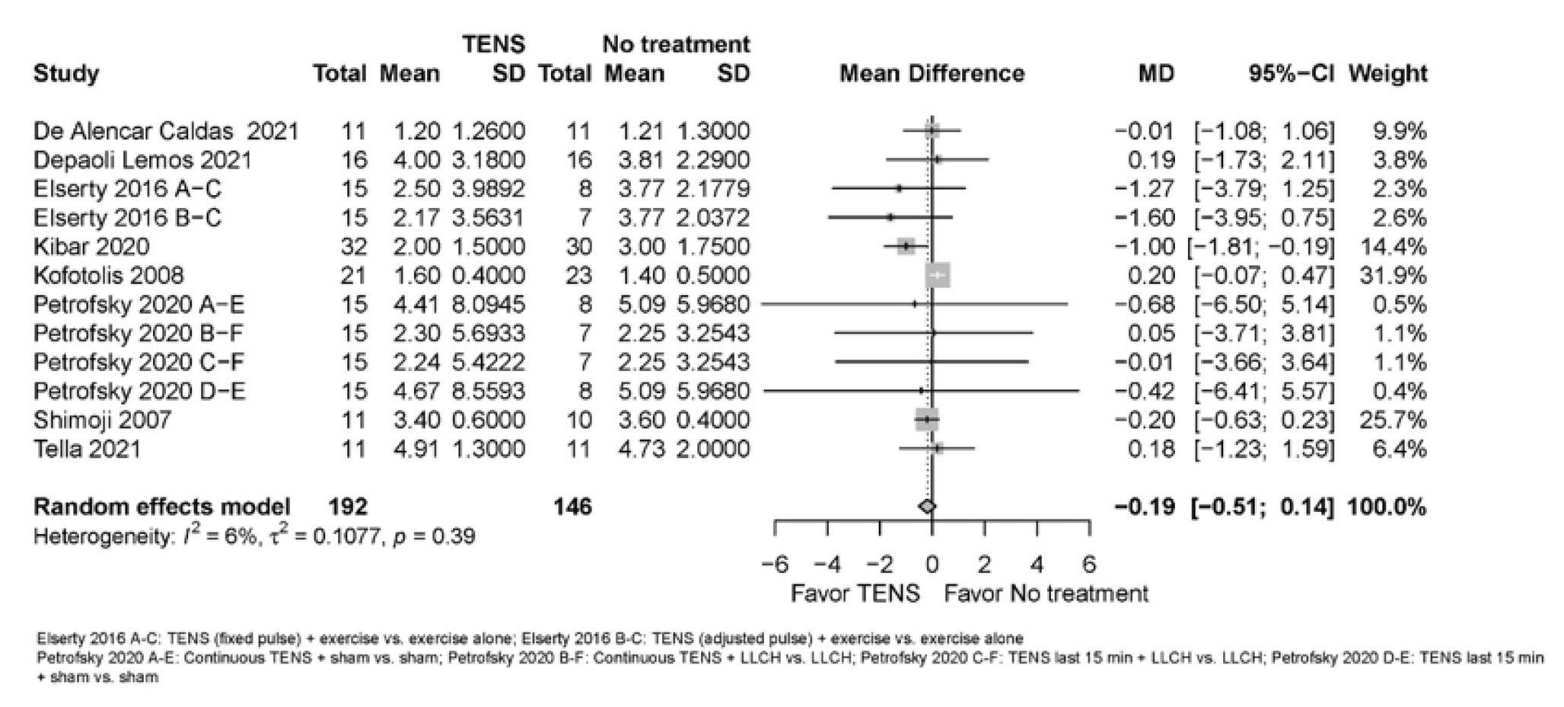
TENS versus no intervention or interventions where the effects of TENS were isolated for pain in the immediate term (closest to 2 weeks); scale range is 0 to 10

Due to very low certainty evidence, it is uncertain whether TENS makes little or no difference to ***function ****(benefit indicated by lower values)* in the immediate (6 RCTs; SMD = -0.32, 95% CI -0.71 to 0.07) (plot 2.2.1) [21, 24, 25, 27, 30, 31], or short term (2 RCTs; SMD = 1.05, 95% CI -18.51 to 20.61) (plot 2.2.2) [29, 31]. It is uncertain whether TENS makes little or no difference to ***health-related quality of life ****(scale 0 to 100; 0 = poor health-related quality of life)* in the immediate term (***PCS***: MD = -6.82, 95% CI -27.06 to 13.42; plot 2.3.1; ***MCS***: MD = -2.91, 95% CI -10.25 to 4.43; plot 2.4.1) [24]. It is uncertain whether TENS makes little or no difference to ***depression ****(scale 0 to 21; 0 = no depression)* in the short term (1 RCT; MD = -1.40, 95% CI -5.57 to 2.77) (plot 2.5.1) [29]. It is uncertain whether TENS reduces ***pain catastrophizing ****(scale 0 to 52; 0 = no catastrophizing)* in the short term (1 RCT; MD = -11.20, 95% CI -17.88 to -3.52) (plot 2.6.1) [30]. It is uncertain whether TENS makes little or no difference to ***adverse events/harms*** in the immediate term (1 RCT) (no plot, narrative synthesis) [31].

### Subgroup and Sensitivity Analyses

The results of the subgroup analyses did not substantially alter our main findings; however, the subgroups require further investigation. For all comparisons, the subgroups were small (consisting of 1–3 RCTs with sample sizes ranging from 11 to 134 adults per group) and yielded small, pooled effects with marked imprecision (wide 95% CIs) and unclear clinical implications. Therefore, subgroup differences could not be explained and/or the differences between subgroups would likely not result in different recommendations for different subgroups (see Online Resource 7). This is due to the very low certainty evidence and little or no differences between TENS and comparisons for all outcomes.

## Discussion

The evidence regarding the benefits and harms of TENS for CPLBP in adults is based on 17 RCTs with a total of 1027 adults. Of these, two RCTs (one report, n = 28) assessed older adults (aged ≥ 60 years) [2]. Most of the RCTs (14, 82%) were rated as having a high overall risk of bias, and three (18%) were rated as unclear overall risk of bias. The certainty of the evidence for all outcomes was very low. For most outcomes there was little or no difference between TENS and sham, no intervention, or interventions where the attributable effect of TENS could be isolated (i.e., combined TENS with treatment B versus treatment B alone). In the comparison of TENS with sham, in the immediate term, evidence suggested a marginal reduction in pain that did not meet our MID (MD = -1) [6] and was not found in the RCTs exclusively assessing older adults. Additionally, no harms were reported, but this was assessed in only one RCT. In the comparison of TENS with no intervention, evidence suggested a reduction in pain catastrophizing in the short term.

Our findings are similar to those in the review by Khadilkar et al. (2008) [4] concluding that the evidence does not support the use of TENS in the routine management of CPLBP. There are, however, some notable methodological differences between these two reviews. First, Khadilkar et al. exclusively included RCTs comparing TENS to sham. We expanded the breadth of the comparisons and identified nine RCTs comparing TENS to no intervention or interventions where the effects of TENS could be isolated. Our review differed in our assessment of the risk of bias. Aside from using a different risk of bias tool, the interpretation of each type of bias differed between reviews. Khadilkar et al. used an arbitrary cut-off score to determine the level of quality (high versus low quality) attributed to each RCT; whereas we based our assessment on the inherent impact that each type of bias could have contributed to the RCT and its interpretation of results [37]. Interestingly, one RCT relevant to both the Khadilkar and current review [34] was judged to be high quality by Khadilkar et al. and high risk of bias (low quality) by our review team.

Since the publication of Khadilkar et al. in 2008, we identified one related systematic review assessing the effectiveness of TENS versus placebo for CLBP, the findings of which aligned with ours. Resende et al. (2018) [5] found low-quality evidence that TENS did not improve function immediately after therapy. No other results were available that specifically assessed the effects of TENS versus placebo in people with CLBP. Our review differed by specifically assessing the effects of TENS (not in combination with IFC) on multiple additional outcomes such as pain, psychological functioning, and social participation including work.

Our systematic review has strengths. To begin with, our team consisted of clinical and methodological experts from around the world, specializing in LBP, systematic reviews, and evidence syntheses. Secondly, our review process involved conducting thorough and peer-reviewed literature searches without any language restrictions. Third, during the screening and ROB assessments, a core team member (with the most expertise and reliability in screening and ROB evaluations) was involved in each screening and ROB pair. Fourth, our ROB assessments did not rely on summary scores or the number of items at risk of bias, as some other systematic reviews have done. Instead, we created supplementary guidance forms based on the ROB1 criteria [12, 13], which allowed reviewers to consider critical flaws in the studies [6]. Our use of these forms resulted in high agreement on overall ROB ratings. Lastly, we maintained transparency throughout the review process, providing detailed ROB assessments and footnotes for grading the certainty of the evidence (see Online Resources 4, 5). These notes give readers a better understanding of our judgements and allow them to reach their own conclusions.

Our review has potential limitations. One limitation is the possibility that we missed relevant RCTs. However, we attempted to address this by using comprehensive and peer-reviewed literature search strategies developed with the assistance of experienced health sciences librarians. Additionally, we searched the reference lists of included RCTs and related systematic reviews. Another limitation is that we did not search the grey literature, which could introduce publication bias as studies published in peer-reviewed journals tend to report larger intervention effects than those in the grey literature [38]. Nonetheless, we believe our review was not impacted by publication bias. We searched for unpublished RCTs in the WHO ICTRP registry and contacted authors of unpublished RCTs. Responses from authors indicated that incomplete RCTs were the main reason for non-publication. Moreover, unpublished studies are known to represent a small proportion of studies and rarely impact results and conclusions [39]. However, it may be important to include such studies in limited scenarios or where there are potential conflicts of interest in published research [39].

Of note, the WHO GDG sought a homogeneous population comprising adults with CPLBP [6]. That is, we excluded RCTs that included post-surgical adults within 12 months post-surgery, adults who had undergone fusion or disc replacement surgery at any time, pregnant individuals, and adults with a clearly determined specific cause for their LBP (e.g., vertebral fracture, malignancy, inflammatory disease). Some reviews use a majority criterion, deeming eligible studies whereby most participants (e.g., ≥ 80%) do not have certain conditions or characteristics. We excluded a highly cited RCT by Deyo et al. (1990) [40] for two reasons. First, it included adults who had surgery or chymopapain therapy (TENS group: 7/65, 11%; sham group: 6/60, 10%), and the results were not stratified based on whether surgery was received, or type of treatment received. Further, they provided no information on the timing of surgery or type of surgery. Second, Deyo et al. included adults with spondylolysis, spondylolisthesis, compression fracture, or scoliosis (TENS group: 7/65, 11%; sham group: 7/60, 12%), and did not stratify their results based on type of LBP (i.e., primary vs. secondary CLBP). While authors were contacted in most cases to clarify study eligibility, this RCT was published 32 years ago; and for pragmatic reasons, authors were not contacted. Nonetheless, we conducted a sensitivity analysis including the results of this RCT and our findings did not change (i.e., we are uncertain if TENS makes little or no difference to pain, function, and adverse events, since the certainty of the evidence is very low). In the current review, no other RCTs were excluded solely for the inclusion of post-surgical participants or participants with secondary CLBP.

We identified several gaps in the evidence that applied across all comparisons involving TENS. First, we did not identify any RCTs reporting on the benefits or harms of TENS beyond the short term (i.e., 3 months after the intervention period). Second, we did not identify any RCTs assessing the effects of TENS on fear avoidance, anxiety, self-efficacy, or social participation including work. Finally, we were unable to assess whether benefits or harms of TENS vary by race/ethnicity. For the comparison of TENS to sham in all adults, we found no RCTs assessing the effects of treatment on catastrophizing behaviours. In older adults (aged ≥ 60 years), two small RCTs assessed pain; we found no RCTs assessing function, health-related quality of life, depression, fear avoidance, change in medication use, falls, or harms. We were also unable to assess whether the benefits or harms of TENS in older adults vary by gender, presence of leg pain/symptoms, or in people from higher versus lower income countries. For the comparison of TENS to no intervention or interventions where the effects of TENS could be isolated, we found no RCTs reporting on fear avoidance behaviours, anxiety, self-efficacy, or social participation including work, and no RCTs in older adults. Finally, we found no RCTs comparing TENS to usual care. These identified gaps may be an avenue for further research, as well as comparing the effects of different doses, frequencies, and durations of TENS. Harms, including those that may be associated with persistent long-term use, should also be investigated systematically.

## Conclusion

Based on very low certainty evidence, adults with CPLBP experienced brief and marginal reductions in pain (not deemed clinically important) and a short-term reduction in pain catastrophizing with the use of TENS. Harms from TENS are unclear, as only one included RCT assessed adverse events. The remaining evidence showed little to no difference in benefits between TENS and the comparison interventions for a range of other outcomes (e.g., function, health-related quality of life, depression). Care plans for patients should be created through shared decision making, considering the scientific data and other contextual factors, such as patient preferences and values.

### Electronic Supplementary Material

Below is the link to the electronic supplementary material.

Supplementary Material 1

## Data Availability

The datasets generated during and/or analysed during the current study are available from the corresponding author on reasonable request.
