# Supplementary material for: Systematic Review to Inform a World Health Organization (WHO) Clinical Practice Guideline: Benefits and Harms of Transcutaneous Electrical Nerve Stimulation (TENS) for Chronic Primary Low Back Pain in Adults
Source: J Occup Rehabil. 2023 Nov 22;33(4):651–60. doi: 10.1007/s10926-023-10121-7 (PMC10684422; doi:10.1007/s10926-023-10121-7)
Supplement: Supplementary file 1 — Supplementary Material 1 [file 10926_2023_10121_MOESM1_ESM.docx]

Systematic review to inform a World Health Organization (WHO) clinical practice guideline: Benefits and harms of transcutaneous electrical nerve stimulation (TENS) for chronic primary low back pain in adults: Supplementary Information

Verville L, Hincapié CA, Southerst D, Yu H, Bussières A, Gross DP, Pereira P, Mior S, Tricco AC, Cedraschi C, Brunton G, Nordin M, Connell G, Shearer HM, Wong JJ, Hofstetter L, Romanelli A, Guist BP, To D, Stuber K, da Silva-Oolup S, Stupar M, Myrtos D, Lee J, DeSouza A, Muñoz Laguna J, Murnaghan K, Cancelliere C

Corresponding authors:

Carol Cancelliere

Institute of Disability and Rehabilitation Research and Faculty of Health Sciences, Ontario Tech University, Oshawa, Ontario, Canada

Email: [carolina.cancelliere@ontariotechu.ca](mailto:carolina.cancelliere@ontariotechu.ca)

Cesar A. Hincapié

EBPI-UWZH Musculoskeletal Epidemiology Research Group, University of Zurich and Balgrist University Hospital, Zurich, Switzerland

Epidemiology, Biostatistics and Prevention Institute (EBPI), University of Zurich, Zurich, Switzerland

University Spine Centre Zurich (UWZH), Balgrist University Hospital and University of Zurich, Zurich, Switzerland

Email: [cesar.hincapie@uzh.ch](mailto:cesar.hincapie@uzh.ch)

**Online Resource 1.** Literature search strategies

**A. Database & Platform:**  MEDLINE (Ovid)

**Years of search:** July 1, 2007 – March 9, 2022

**Date search run:**  March 9, 2022 **Number of records retrieved:** 621

**Search Strategy:**

1. Low Back Pain/
2. exp Back Pain/
3. Back Injuries/
4. exp Back Muscles/in [Injuries]
5. Intervertebral Disc Degeneration/
6. Intervertebral Disc Displacement/
7. Intervertebral Disc/in [Injuries]
8. Lumbar Vertebrae/in [Injuries]
9. Lumbosacral Plexus/in [Injuries]
10. Lumbosacral Region/in [Injuries]
11. Coccyx/in [Injuries]
12. Osteoarthritis, Spine/
13. Osteoarthritis/
14. Piriformis Muscle Syndrome/
15. Polyradiculopathy/
16. Sacroiliac Joint/in [Injuries]
17. Sciatica/
18. Spinal Curvatures/
19. Spinal Diseases/
20. Spinal Injuries/
21. Spinal Stenosis/
22. exp Spondylolysis/
23. Spondylosis/
24. Synovial Cyst/
25. Zygaphophyseal Joint/in [Injuries]
26. ((low* adj2 (back adj2 pain*)) or (low-back* adj2 pain*) or (lower-back* adj2 pain*) or (low* adj2 back-pain*)).mp.
27. ((low* adj2 (back adj2 injur*)) or (low-back* adj2 injur*) or (lower-back* adj2 injur*) or (low* adj2 back-injur*)).mp.
28. ((low* adj2 (back adj2 trauma*)) or (low-back adj2 trauma*) or (lower-back* adj2 trauma*) or (low* adj2 back-trauma*)).mp.
29. ((low* adj2 (trunk adj2 pain*)) or (lower-trunk* adj2 pain*) or (low* adj2 trunk-pain*)).mp.
30. lumbar* adj3 (disc* adj3 (extru* or degenerat* or displac* or herniat* or prolaps* or sequestered or slipped or protru* or avuls*)).mp.
31. lumbar* adj3 (disk* adj3 (extru* or degenerat* or displac* or herniat* or prolaps* or sequestered or slipped or protru* or avuls*)).mp.
32. lumbar* adj3 (pain* or facet* or (nerve adj2 root*) or osteoarth* or radicul* or stenos* or spondylo* or zygapophys* or injur* or discomfort* or dysfunction* or sore* or herniat*).mp.
33. lumbo* adj3 (pain* or facet* or (nerve adj2 root*) or osteoarth* or radicul* or stenos* or spondylo* or zygapophys* or injur* or discomfort* or dysfunction* or sore* or herniat*).mp.
34. back adj3 (ach* or injur* or pain* or sprain* or strain* or disorder*).mp.
35. backach*.mp.
36. back-pain*.mp.
37. intervertebral* adj3 (disc* adj3 (extru* or degenerat* or displac* or herniat* or prolaps* or sequestered or slipped or protru* or avuls*)).mp.
38. intervertebral* adj3 (disk* adj3 (extru* or degenerat* or displac* or herniat* or prolaps* or sequestered or slipped or protru* or avuls*)).mp.
39. coccy* adj2 (ach* or injur* or pain* or sprain* or strain*).mp.
40. (coccygodyn* or coccalg* or coccygalg*).mp.
41. dorsalg*.mp.
42. lumbago*.mp.
43. lumboischialg*.mp.
44. (piriformis* adj2 syndrome*).mp.
45. sacral* adj3 (pain* or facet* or (nerve adj2 root*) or osteoarth* or radicul* or stenos* or spondylo* or zygapophys* or injur* or discomfort* or dysfunction* or sore* or herniat*).mp.
46. sacro* adj3 (pain* or facet* or (nerve adj2 root*) or osteoarth* or radicul* or stenos* or spondylo* or zygapophys* or injur* or discomfort* or dysfunction* or sore* or herniat*).mp.
47. "si" adj2 (joint* adj3 (pain* or facet* or (nerve adj2 root*) or osteoarth* or radicul* or stenos* or spondylo* or zygapophys* or injur* or discomfort* or dysfunction* or sore* or herniat*)).mp.
48. sacrococcy* adj2 (ach* or injur* or pain* or sprain* or strain*).mp.
49. sacrum* adj2 (ach* or injur* or pain* or sprain* or strain*).mp.
50. sciatic*.mp.
51. stenos* adj2 (spine* or spinal* or vertebral*).mp.
52. (spine* or spinal*) adj2 osteoarthr*.mp.
53. spine* adj3 (condition* or diseas* or disabilit* or disorder* or degenerat* or pain* or stenos*).mp.
54. spinal* adj3 (condition* or diseas* or disabilit* or disorder* or degenerat* or pain* or stenos*).mp.
55. spondylo*.mp.
56. tailbone* adj3 (ach* or injur* or pain* or sprain* or strain*).mp.
57. vertebr* adj3 (ach* or injur* or pain* or sprain* or strain*).mp
58. poly-radicul* or polyradicul*.mp.
59. neuropath* adj2 (lumbar* or lumbo* or sacral* or sacro* or (low* adj2 back) or low-back* or lower-back* or spine* or spinal* or L1 or L2 or L3 or L4 or L5).mp.
60. radiculopath* adj3 (lumbar* or lumbo* or sacral* or sacro* or (low* adj2 back) or low-back* or lower-back* or spine* or spinal* or L1 or L2 or L3 or L4 or L5).mp.
61. radiating* adj3 (lumbar* or lumbo* or sacral* or sacro* or (low* adj2 back) or low-back* or lower-back* or spine* or spinal* or L1 or L2 or L3 or L4 or L5).mp.
62. radicular* adj3 (lumbar* or lumbo* or sacral* or sacro* or (low* adj2 back) or low-back* or lower-back* or spine* or spinal* or L1 or L2 or L3 or L4 or L5).mp.
63. lumborum* adj3 (ach* or injur* or pain* or sprain* or strain*).mp.
64. longissimus* adj3 (ach* or injur* or pain* or sprain* or strain*).mp.
65. (erector adj2 spin*) adj3 (ach* or injur* or pain* or sprain* or strain*).mp.
66. synovial* adj2 cyst*.mp.
67. thoracolumbar* adj3 (pain* or facet* or (nerve* adj2 root*) or osteoarthr* or radicul* or stenos* or spondylo* or zygapohys* or injur* or trauma* or discomfort* or dysfunction* or sore* or herniat*).mp.
68. thoraco-lumbar* adj3 (pain* or facet* or (nerve* adj2 root*) or osteoarthr* or radicul* or stenos* or spondylo* or zygapohys* or injur* or trauma* or discomfort* or dysfunction* or sore* or herniat*).mp.
69. curvatur* adj2 (spine* or spinal*).mp.
70. (pathol* adj2 (lumbar* or (low* adj2 back) or low-back* or (lower* adj2 back) or lower-back* or thoracolumbar* or thoraco-lumbar* or intervertebral* or lumbosacral* or lumbo-sacral* or sacral* or sacro-iliac* or sacroiliac*)).mp.
71. or/1-70
72. Transcutaneous Electric Nerve Stimulation/
73. Electric Stimulation Therapy/
74. Electric Stimulation/
75. TENS.mp.
76. ALTENS.mp.
77. TNS.mp.
78. TENMS.mp.
79. C-TENS.mp.
80. A-TENS.mp.
81. transcutan* adj3 (electric* or nerve* or stimul* or neurostimul*).mp.
82. trans-cutan* adj3 (electric* or nerve* or stimul* or neurostimul*).mp.
83. transdermal* adj3 (electric* or nerve* or stimul* or neurostimul*).mp.
84. trans-dermal* adj3 (electric* or nerve* or stimul* or neurostimul*).mp.
85. (peripheral* adj2 (conditioning adj2 stimul*)).mp.
86. (microamperage* adj2 stimul*).mp.
87. (transabdominal adj3 neurostimul*).mp.
88. (trans-abdominal adj3 neurostimul*).mp.
89. electroanalges*.mp.
90. electro-analges*.mp.
91. electrotherap*.mp.
92. electro-therap*.mp.
93. (electr* adj2 (stimul*)).mp.
94. or/ 72-93
95. exp Randomized Controlled Trial/
96. exp Randomized Controlled Trials as Topic/
97. Controlled Clinical Trial/
98. exp Controlled Clinical Trials as Topic/
99. exp Clinical Trials as Topic/
100. exp Clinical Trial/
101. Double-Blind Method/
102. Single-Blind Method/
103. Cross-Over Trials/
104. Placebos/
105. Placebo Effect/
106. Random Allocation/
107. random*.mp.
108. clinical* adj2 trial*mp.
109. controlled* adj2 (trial*).mp.
110. blind* adj2 (doubl* or singl*).mp.
111. placebo*.mp.
112. (crossover* or cross-over*).mp.
113. randomized controlled trial.pt.
114. controlled clinical trial.pt.
115. clinical trial.pt.
116. or/ 95-1115
117. 71 AND 94 AND 116
118. exp Animals/
119. exp Humans/
120. 118 NOT 119
121. Limit 117 NOT 120
122. (comment or clinical conference or congress or consensus development conference or editorial or letter or review or systematic review or guideline or practice guideline or case reports).pt.
123. Limit 121 NOT 122
124. Limit 123 to dt=20070701-20220301
125. Limit 123 to rd=20070701-20220309
126. 124 or 125

**B. Database & Platform:**  CINAHL (EBSCO)

**Years of search:** July 1, 2007- March 9, 2022

**Date search run:**  March 9, 2022, **Number of records retrieved:**  341

**Search Strategy:**

1. MH Low Back Pain
2. MH Back Pain+
3. MH Back Injuries
4. MH Intervertebral Disc Displacement
5. MH Intervertebral Disc/IN
6. MH Lumbar Vertebrae/IN
7. MH Lumbosacral Plexus/IN
8. MH Coccyx/IN
9. MH Osteoarthritis, Spine
10. MH Osteoarthritis
11. MH Piriformis Muscles/IN
12. MH Polyradiculopathy/
13. MH Sacroiliac Joint/IN
14. MH Sciatica
15. MH Spinal Curvatures
16. MH Spinal Diseases
17. MH Spinal Injuries
18. MH Spinal Stenosis
19. MH Spondylolysis+
20. MH Spondylosis
21. MH Synovial Cyst
22. MH Zygaphophyseal Joint/IN
23. TI ((low* n2 (back n2 pain*)) or (low-back* n2 pain*) or (lower-back* n2 pain*) or (low* n2 back-pain*)) or AB ((low* n2 (back n2 pain*)) or (low-back* n2 pain*) or (lower-back* n2 pain*) or (low* n2 back-pain*))
24. TI ((low* n2 (back n2 injur*)) or (low-back* n2 injur*) or (lower-back* n2 injur*) or (low* n2 back-injur*)) or AB ((low* n2 (back n2 injur*)) or (low-back* n2 injur*) or (lower-back* n2 injur*) or (low* n2 back-injur*))
25. TI ((low* n2 (back n2 trauma*)) or (low-back n2 trauma*) or (lower-back* n2 trauma*) or (low* n2 back-trauma*)) or AB ((low* n2 (back n2 trauma*)) or (low-back n2 trauma*) or (lower-back* n2 trauma*) or (low* n2 back-trauma*))
26. TI ((low* n2 (trunk n2 pain*)) or (lower-trunk* n2 pain*) or (low* n2 trunk-pain*)) or AB ((low* n2 (trunk n2 pain*)) or (lower-trunk* n2 pain*) or (low* n2 trunk-pain*))
27. TI lumbar* n3 (disc* n3 (extru* or degenerat* or displac* or herniat* or prolaps* or sequestered or slipped or protru* or avuls*)) or AB lumbar* n3 (disc* n3 (extru* or degenerat* or displac* or herniat* or prolaps* or sequestered or slipped or protru* or avuls*))
28. TI lumbar* n3 (disk* n3 (extru* or degenerat* or displac* or herniat* or prolaps* or sequestered or slipped or protru* or avuls*)) or AB lumbar* n3 (disk* n3 (extru* or degenerat* or displac* or herniat* or prolaps* or sequestered or slipped or protru* or avuls*))
29. TI lumbar* n3 (pain* or facet* or (nerve n2 root*) or osteoarth* or radicul* or stenos* or spondylo* or zygapophys* or injur* or discomfort* or dysfunction* or sore* or herniat*) or AB lumbar* n3 (pain* or facet* or (nerve n2 root*) or osteoarth* or radicul* or stenos* or spondylo* or zygapophys* or injur* or discomfort* or dysfunction* or sore* or herniat*)
30. TI lumbo* n3 (pain* or facet* or (nerve n2 root*) or osteoarth* or radicul* or stenos* or spondylo* or zygapophys* or injur* or discomfort* or dysfunction* or sore* or herniat*) or AB lumbo* n3 (pain* or facet* or (nerve n2 root*) or osteoarth* or radicul* or stenos* or spondylo* or zygapophys* or injur* or discomfort* or dysfunction* or sore* or herniat*)
31. TI back n3 (ach* or injur* or pain* or sprain* or strain* or disorder*) or AB back n3 (ach* or injur* or pain* or sprain* or strain* or disorder
32. TI backach* or AB backach*
33. TI back-pain* or AB back-pain*
34. TI intervertebral* n3 (disc* n3 (extru* or degenerat* or displac* or herniat* or prolaps* or sequestered or slipped or protru* or avuls*)) or AB intervertebral* n3 (disc* n3 (extru* or degenerat* or displac* or herniat* or prolaps* or sequestered or slipped or protru* or avuls*))
35. TI intervertebral* n3 (disk* n3 (extru* or degenerat* or displac* or herniat* or prolaps* or sequestered or slipped or protru* or avuls*)) or AB intervertebral* n3 (disk* n3 (extru* or degenerat* or displac* or herniat* or prolaps* or sequestered or slipped or protru* or avuls*))
36. TI coccy* n2 (ach* or injur* or pain* or sprain* or strain*) or AB coccy* n2 (ach* or injur* or pain* or sprain* or strain*)
37. TI (coccygodyn* or coccalg* or coccygalg*) or AB (coccygodyn* or coccalg* or coccygalg*)
38. TI dorsalg* or AB dorsalg*
39. TI lumbago* or AB lumbago*
40. TI lumboischialg* or AB lumboischialg*
41. TI (piriformis* n2 syndrome*) or AB (piriformis* n2 syndrome*)
42. TI sacral* n3 (pain* or facet* or (nerve n2 root*) or osteoarth* or radicul* or stenos* or spondylo* or zygapophys* or injur* or discomfort* or dysfunction* or sore* or herniat*) or AB sacral* n3 (pain* or facet* or (nerve n2 root*) or osteoarth* or radicul* or stenos* or spondylo* or zygapophys* or injur* or discomfort* or dysfunction* or sore* or herniat*)
43. TI sacro* n3 (pain* or facet* or (nerve n2 root*) or osteoarth* or radicul* or stenos* or spondylo* or zygapophys* or injur* or discomfort* or dysfunction* or sore* or herniat*) or AB sacro* n3 (pain* or facet* or (nerve n2 root*) or osteoarth* or radicul* or stenos* or spondylo* or zygapophys* or injur* or discomfort* or dysfunction* or sore* or herniat*)
44. TI "si" n2 (joint* n3 (pain* or facet* or (nerve n2 root*) or osteoarth* or radicul* or stenos* or spondylo* or zygapophys* or injur* or discomfort* or dysfunction* or sore* or herniat*)) or AB "si" n2 (joint* n3 (pain* or facet* or (nerve n2 root*) or osteoarth* or radicul* or stenos* or spondylo* or zygapophys* or injur* or discomfort* or dysfunction* or sore* or herniat*))
45. TI sacrococcy* n2 (ach* or injur* or pain* or sprain* or strain*) or AB sacrococcy* n2 (ach* or injur* or pain* or sprain* or strain*)
46. TI sacrum* n2 (ach* or injur* or pain* or sprain* or strain*) or AB sacrum* n2 (ach* or injur* or pain* or sprain* or strain*)
47. TI sciatic* or sciatic*
48. TI stenos* n2 (spine* or spinal* or vertebral*) or AB stenos* n2 (spine* or spinal* or vertebral*)
49. TI (spine* or spinal*) n2 osteoarthr* or AB (spine* or spinal*) n2 osteoarthr*
50. TI spine* n3 (condition* or diseas* or disabilit* or disorder* or degenerat* or pain* or stenos*) or AB spine* n3 (condition* or diseas* or disabilit* or disorder* or degenerat* or pain* or stenos*)
51. TI spinal* n3 (condition* or diseas* or disabilit* or disorder* or degenerat* or pain* or stenos*) or AB spinal* n3 (condition* or diseas* or disabilit* or disorder* or degenerat* or pain* or stenos*)
52. TI spondylo* or AB spondylo*
53. TI tailbone* n3 (ach* or injur* or pain* or sprain* or strain*) or AB tailbone* n3 (ach* or injur* or pain* or sprain* or strain*)
54. TI vertebr* n3 (ach* or injur* or pain* or sprain* or strain*) or AB vertebr* n3 (ach* or injur* or pain* or sprain* or strain*)
55. TI (poly-radicul* or polyradicul*) or AB (poly-radicul* or polyradicul*)
56. TI neuropath* n2 (lumbar* or lumbo* or sacral* or sacro* or (low* n2 back) or low-back* or lower-back* or spine* or spinal* or L1 or L2 or L3 or L4 or L5) or AB neuropath* n2 (lumbar* or lumbo* or sacral* or sacro* or (low* n2 back) or low-back* or lower-back* or spine* or spinal* or L1 or L2 or L3 or L4 or L5)
57. TI radiculopath* n3 (lumbar* or lumbo* or sacral* or sacro* or (low* n2 back) or low-back* or lower-back* or spine* or spinal* or L1 or L2 or L3 or L4 or L5) or AB radiculopath* n3 (lumbar* or lumbo* or sacral* or sacro* or (low* n2 back) or low-back* or lower-back* or spine* or spinal* or L1 or L2 or L3 or L4 or L5)
58. TI radiating* n3 (lumbar* or lumbo* or sacral* or sacro* or (low* n2 back) or low-back* or lower-back* or spine* or spinal* or L1 or L2 or L3 or L4 or L5) or AB radiating* n3 (lumbar* or lumbo* or sacral* or sacro* or (low* n2 back) or low-back* or lower-back* or spine* or spinal* or L1 or L2 or L3 or L4 or L5)
59. TI radicular* n3 (lumbar* or lumbo* or sacral* or sacro* or (low* n2 back) or low-back* or lower-back* or spine* or spinal* or L1 or L2 or L3 or L4 or L5) or AB radicular* n3 (lumbar* or lumbo* or sacral* or sacro* or (low* n2 back) or low-back* or lower-back* or spine* or spinal* or L1 or L2 or L3 or L4 or L5)
60. TI lumborum* n3 (ach* or injur* or pain* or sprain* or strain*) or AB lumborum* n3 (ach* or injur* or pain* or sprain* or strain*)
61. TI longissimus* n3 (ach* or injur* or pain* or sprain* or strain*) or AB longissimus* n3 (ach* or injur* or pain* or sprain* or strain*)
62. TI (erector n2 spin*) n3 (ach* or injur* or pain* or sprain* or strain*) or AB erector n2 spin*) n3 (ach* or injur* or pain* or sprain* or strain*)
63. TI synovial* n2 cyst* or AB synovial* n2 cyst*
64. TI thoracolumbar* n3 (pain* or facet* or (nerve* n2 root*) or osteoarthr* or radicul* or stenos* or spondylo* or zygapohys* or injur* or trauma* or discomfort* or dysfunction* or sore* or herniat*) or AB thoracolumbar* n3 (pain* or facet* or (nerve* n2 root*) or osteoarthr* or radicul* or stenos* or spondylo* or zygapohys* or injur* or trauma* or discomfort* or dysfunction* or sore* or herniat*)
65. TI thoraco-lumbar* n3 (pain* or facet* or (nerve* n2 root*) or osteoarthr* or radicul* or stenos* or spondylo* or zygapohys* or injur* or trauma* or discomfort* or dysfunction* or sore* or herniat*) or AB thoraco-lumbar* n3 (pain* or facet* or (nerve* n2 root*) or osteoarthr* or radicul* or stenos* or spondylo* or zygapohys* or injur* or trauma* or discomfort* or dysfunction* or sore* or herniat*)
66. TI curvatur* n2 (spine* or spinal*) or AB curvatur* n2 (spine* or spinal*)
67. TI (pathol* n2 (lumbar* or (low* n2 back) or low-back* or (lower* n2 back) or lower-back* or thoracolumbar* or thoraco-lumbar* or intervertebral* or lumbosacral* or lumbo-sacral* or sacral* or sacro-iliac* or sacroiliac*)) or AB (pathol* n2 (lumbar* or (low* n2 back) or low-back* or (lower* n2 back) or lower-back* or thoracolumbar* or thoraco-lumbar* or intervertebral* or lumbosacral* or lumbo-sacral* or sacral* or sacro-iliac* or sacroiliac*))
68. or/1-67
69. MH Transcutaneous Electric Nerve Stimulation
70. MH Electrotherapy
71. MH Electric Stimulation
72. TI TENS or AB TENS
73. TI ALTENS or AB ALTENS
74. TI TNS or AB TNS
75. TI TENMS or AB TENMS
76. TI C-TENS or C-TENS
77. TI A-TENS or A-TENS
78. TI transcutan* n3 (electric* or nerve* or stimul* or neurostimul*) or AB anscutan* n3 (electric* or nerve* or stimul* or neurostimul*)
79. TI trans-cutan* n3 (electric* or nerve* or stimul* or neurostimul*) or AB trans-cutan* n3 (electric* or nerve* or stimul* or neurostimul*)
80. TI transdermal* n3 (electric* or nerve* or stimul* or neurostimul*) or AB transdermal* n3 (electric* or nerve* or stimul* or neurostimul*)
81. TI trans-dermal* n3 (electric* or nerve* or stimul* or neurostimul*) or AB trans-dermal* n3 (electric* or nerve* or stimul* or neurostimul*)
82. TI (peripheral* n2 (conditioning n2 stimul*)) or AB (peripheral* n2 (conditioning n2 stimul*))
83. TI (microamperage* n2 stimul*) or AB (microamperage* n2 stimul*)
84. TI (transabdominal n3 neurostimul*) or AB (transabdominal n3 neurostimul*)
85. TI (trans-abdominal n3 neurostimul*) or AB (trans-abdominal n3 neurostimul*)
86. TI electroanalges* or AB electroanalges*
87. TI electro-analges* or AB electro-analges*
88. TI electrotherap* or AB electrotherap*
89. TI electro-therap* or AB electro-therap*
90. TI (electr* n2 (stimul*)) or AB (electr* n2 (stimul*))
91. or/ 69-90
92. MH Randomized Controlled Trials+
93. MH Clinical Trials+
94. MH Double-Blind Trials
95. MH Single-Blind Trials
96. MH Crossover Design
97. MH Placebos
98. MH Placebo Effect
99. MH Random Assignment
100. TI random* or AB random*
101. TI clinical* n2 (trial* or trial* or trials*) or AB clinical* n2 (trial* or trial* or trials*)
102. TI controlled* n2 (trial* or trial* or trials*) or AB controlled* n2 (trial* or trial* or trials*)
103. TI blind* n2 (doubl* or singl*) or AB blind* n2 (doubl* or singl*)
104. TI placebo* or AB placebo*
105. TI (crossover* or cross-over*) or AB (crossover* or cross-over*)
106. PT randomized controlled trial
107. PT controlled clinical trial
108. PT clinical trial
109. or/ 92-108
110. 68 AND 91 AND 109
111. MH Animals+
112. MH Human
113. 111 NOT 112
114. Limit 110 NOT 113
115. 114 NOT PT (abstract or brief item or book review or case trial or commentary or doctoral dissertation or editorial or letter or practice guidelines or proceedings or review or systematic review)
116. Limit 121 NOT 122
117. 116 AND EM=20070701-20220309
118. 116 AND RD=20070701-20220309
119. 117 OR 118

**C.** **Database & Platform:**  EMBASE (Ovid)

**Years of search:** July 1, 2007 - March 9, 2022

**Date search run:**  March 9, 2022, **Number of records retrieved:** 130

**Search Strategy:**

1. Low Back Pain/
2. exp Backache/
3. Intervertebral Disc Degeneration/
4. Intervertebral Disk Hernia/
5. Lumbar Disk Hernia/
6. Coccyx/
7. Spondylosis/
8. Spondylolysis/
9. Osteoarthritis/
10. Lumbar Spinal Stenosis/
11. Synovial Cyst/
12. Piriformis Syndrome/
13. Sciatica/
14. ((low* adj2 (back adj2 pain*)) or (low-back* adj2 pain*) or (lower-back* adj2 pain*) or (low* adj2 back-pain*)).mp.
15. ((low* adj2 (back adj2 injur*)) or (low-back* adj2 injur*) or (lower-back* adj2 injur*) or (low* adj2 back-injur*)).mp.
16. ((low* adj2 (back adj2 trauma*)) or (low-back adj2 trauma*) or (lower-back* adj2 trauma*) or (low* adj2 back-trauma*)).mp.
17. ((low* adj2 (trunk adj2 pain*)) or (lower-trunk* adj2 pain*) or (low* adj2 trunk-pain*)).mp.
18. lumbar* adj3 (disc* adj3 (extru* or degenerat* or displac* or herniat* or prolaps* or sequestered or slipped or protru* or avuls*)).mp.
19. lumbar* adj3 (disk* adj3 (extru* or degenerat* or displac* or herniat* or prolaps* or sequestered or slipped or protru* or avuls*)).mp.
20. lumbar* adj3 (pain* or facet* or (nerve adj2 root*) or osteoarth* or radicul* or stenos* or spondylo* or zygapophys* or injur* or discomfort* or dysfunction* or sore* or herniat*).mp.
21. lumbo* adj3 (pain* or facet* or (nerve adj2 root*) or osteoarth* or radicul* or stenos* or spondylo* or zygapophys* or injur* or discomfort* or dysfunction* or sore* or herniat*).mp.
22. back adj3 (ach* or injur* or pain* or sprain* or strain* or disorder*).mp.
23. backach*.mp.
24. back-pain*.mp.
25. intervertebral* adj3 (disc* adj3 (extru* or degenerat* or displac* or herniat* or prolaps* or sequestered or slipped or protru* or avuls*)).mp.
26. intervertebral* adj3 (disk* adj3 (extru* or degenerat* or displac* or herniat* or prolaps* or sequestered or slipped or protru* or avuls*)).mp.
27. coccy* adj2 (ach* or injur* or pain* or sprain* or strain*).mp.
28. (coccygodyn* or coccalg* or coccygalg*).mp.
29. dorsalg*.mp.
30. lumbago*.mp.
31. lumboischialg*.mp.
32. (piriformis* adj2 syndrome*).mp.
33. sacral* adj3 (pain* or facet* or (nerve adj2 root*) or osteoarth* or radicul* or stenos* or spondylo* or zygapophys* or injur* or discomfort* or dysfunction* or sore* or herniat*).mp.
34. sacro* adj3 (pain* or facet* or (nerve adj2 root*) or osteoarth* or radicul* or stenos* or spondylo* or zygapophys* or injur* or discomfort* or dysfunction* or sore* or herniat*).mp.
35. "si" adj2 (joint* adj3 (pain* or facet* or (nerve adj2 root*) or osteoarth* or radicul* or stenos* or spondylo* or zygapophys* or injur* or discomfort* or dysfunction* or sore* or herniat*)).mp.
36. sacrococcy* adj2 (ach* or injur* or pain* or sprain* or strain*).mp.
37. sacrum* adj2 (ach* or injur* or pain* or sprain* or strain*).mp.
38. sciatic*.mp.
39. stenos* adj2 (spine* or spinal* or vertebral*).mp.
40. (spine* or spinal*) adj2 osteoarthr*.mp.
41. spine* adj3 (condition* or diseas* or disabilit* or disorder* or degenerat* or pain* or stenos*).mp.
42. spinal* adj3 (condition* or diseas* or disabilit* or disorder* or degenerat* or pain* or stenos*).mp.
43. spondylo*.mp.
44. tailbone* adj3 (ach* or injur* or pain* or sprain* or strain*).mp.
45. vertebr* adj3 (ach* or injur* or pain* or sprain* or strain*).mp
46. poly-radicul* or polyradicul*.mp.
47. neuropath* adj2 (lumbar* or lumbo* or sacral* or sacro* or (low* adj2 back) or low-back* or lower-back* or spine* or spinal* or L1 or L2 or L3 or L4 or L5).mp.
48. radiculopath* adj3 (lumbar* or lumbo* or sacral* or sacro* or (low* adj2 back) or low-back* or lower-back* or spine* or spinal* or L1 or L2 or L3 or L4 or L5).mp.
49. radiating* adj3 (lumbar* or lumbo* or sacral* or sacro* or (low* adj2 back) or low-back* or lower-back* or spine* or spinal* or L1 or L2 or L3 or L4 or L5).mp.
50. radicular* adj3 (lumbar* or lumbo* or sacral* or sacro* or (low* adj2 back) or low-back* or lower-back* or spine* or spinal* or L1 or L2 or L3 or L4 or L5).mp.
51. lumborum* adj3 (ach* or injur* or pain* or sprain* or strain*).mp.
52. longissimus* adj3 (ach* or injur* or pain* or sprain* or strain*).mp.
53. (erector adj2 spin*) adj3 (ach* or injur* or pain* or sprain* or strain*).mp.
54. synovial* adj2 cyst*.mp.
55. thoracolumbar* adj3 (pain* or facet* or (nerve* adj2 root*) or osteoarthr* or radicul* or stenos* or spondylo* or zygapohys* or injur* or trauma* or discomfort* or dysfunction* or sore* or herniat*).mp.
56. thoraco-lumbar* adj3 (pain* or facet* or (nerve* adj2 root*) or osteoarthr* or radicul* or stenos* or spondylo* or zygapohys* or injur* or trauma* or discomfort* or dysfunction* or sore* or herniat*).mp.
57. curvatur* adj2 (spine* or spinal*).mp.
58. (pathol* adj2 (lumbar* or (low* adj2 back) or low-back* or (lower* adj2 back) or lower-back* or thoracolumbar* or thoraco-lumbar* or intervertebral* or lumbosacral* or lumbo-sacral* or sacral* or sacro-iliac* or sacroiliac*)).mp.
59. or/1-58
60. Transcutaneous Electric Nerve Stimulation/
61. Electrotherapy/
62. Electrostimulation/
63. TENS.mp.
64. ALTENS.mp.
65. TNS.mp.
66. TENMS.mp.
67. C-TENS.mp.
68. A-TENS.mp.
69. transcutan* adj3 (electric* or nerve* or stimul* or neurostimul*).mp.
70. trans-cutan* adj3 (electric* or nerve* or stimul* or neurostimul*).mp.
71. transdermal* adj3 (electric* or nerve* or stimul* or neurostimul*).mp.
72. trans-dermal* adj3 (electric* or nerve* or stimul* or neurostimul*).mp.
73. (peripheral* adj2 (conditioning adj2 stimul*)).mp.
74. (microamperage* adj2 stimul*).mp.
75. (transabdominal adj3 neurostimul*).mp.
76. (trans-abdominal adj3 neurostimul*).mp.
77. electroanalges*.mp.
78. electro-analges*.mp.
79. electrotherap*.mp.
80. electro-therap*.mp.
81. (electr* adj2 (stimul*)).mp.
82. (electrostimulat* or electro-stimulat*).mp.
83. or/ 60-82
84. exp Randomized Controlled Trial/
85. exp Randomized Controlled Trial (Topic)/
86. Controlled Clinical Trial/
87. exp Controlled Clinical Trial (Topic)/
88. exp Clinical Trial (Topic)/
89. exp Clinical Trial/
90. Double-Blind Procedure/
91. Single-Blind Procedure/
92. Crossover Procedure/
93. Placebo/
94. Placebo Effect/
95. Randomization/
96. random*.mp.
97. clinical* adj2 trial*mp.
98. controlled* adj2 (trial*).mp.
99. blind* adj2 (doubl* or singl*).mp.
100. placebo*.mp.
101. (crossover* or cross-over*).mp.
102. or/ 84-101
103. 59 AND 83 AND 102
104. exp Animal/
105. exp Human/
106. 104 NOT 105
107. Limit 103 NOT 106
108. (books or chapter or conference abstract or conference paper or conference review or review or editorial or letter).pt.
109. Limit 107 NOT 108
110. Limit 109 to dd=20070701-20220301
111. Limit 109 to rd=20070701-20220309
112. 110 or 111

**D. Database & Platform:**  Cochrane Central Register of Controlled Trials (Wiley)

**Years of search:** July 2007 – March 2022

**Date search run:**  March 9, 2022, **Number of records retrieved:** 832

**Search Strategy:**

#1 MeSH descriptor: [Back Injuries] explode all trees

#2 MeSH descriptor: [Back Pain] explode all trees

#3 MeSH descriptor: [Low Back Pain] this term only

#4 MeSH descriptor: [Osteoarthritis] this term only

#5 MeSH descriptor: [Osteoarthritis, Spine] this term only

#6 MeSH descriptor: [Piriformis Muscle Syndrome] this term only

#7 MeSH descriptor: [Polyradiculopathy] this term only

#8 MeSH descriptor: [Spinal Diseases] explode all trees

#9 MeSH descriptor: [Synovial Cyst] this term only

#10 MeSH descriptor: [Back Muscles] this term only and with qualifier(s): [injuries - IN]

#11 MeSH descriptor: [Intervertebral Disc Degeneration] this term only

#12 MeSH descriptor: [Intervertebral Disc Displacement] this term only

#13 MeSH descriptor: [Intervertebral Disc] this term only and with qualifier(s): [injuries - IN]

#14 MeSH descriptor: [Lumbar Vertebrae] this term only and with qualifier(s): [injuries - IN]

#15 MeSH descriptor: [Lumbosacral Plexus] this term only and with qualifier(s): [injuries - IN]

#16 MeSH descriptor: [Sacroiliac Joint] this term only and with qualifier(s): [injuries - IN]

#17 MeSH descriptor: [Sacrum] this term only and with qualifier(s): [injuries - IN]

#18 MeSH descriptor: [Lumbosacral Region] this term only and with qualifier(s): [injuries - IN]

#19 MeSH descriptor: [Zygapophyseal Joint] this term only and with qualifier(s): [injuries - IN]

#20 MeSH descriptor: [Sciatica] this term only

#21 MeSH descriptor: [Spinal Injuries] this term only

#22 MeSH descriptor: [Spinal Stenosis] this term only

#23 MeSH descriptor: [Spondylolysis] explode all trees

#24 (((low* near/2 (back near/2 pain*)) or (low-back* near/2 pain*) or (lower-back* near/2 pain*) or (low* near/2 back-pain*))):ti OR (((low* near/2 (back near/2 pain*)) or (low-back* near/2 pain*) or (lower-back* near/2 pain*) or (low* near/2 back-pain*))):ab

#25 (((low* near/2 (back near/2 injur*)) or (low-back* near/2 injur*) or (lower-back* near/2 injur*) or (low* near/2 back-injur*))):ti OR (((low* near/2 (back near/2 injur*)) or (low-back* near/2 injur*) or (lower-back* near/2 injur*) or (low* near/2 back-injur*))):ab

#26 (((low* near/2 (back near/2 trauma*)) or (low-back near/2 trauma*) or (lower-back* near/2 trauma*) or (low* near/2 back-trauma*))):ti OR (((low* near/2 (back near/2 trauma*)) or (low-back near/2 trauma*) or (lower-back* near/2 trauma*) or (low* near/2 back-trauma*))):ab

#27 (((low* near/2 (trunk near/2 pain*)) or (lower-trunk* near/2 pain*) or (low* near/2 trunk-pain*))):ti OR (((low* near/2 (trunk near/2 pain*)) or (lower-trunk* near/2 pain*) or (low* near/2 trunk-pain*))):ab

#28 (lumbar* near/3 (disc* near/3 (extru* or degenerat* or displac* or herniat* or prolaps* or sequestered or slipped or protru* or avuls*))):ti OR (lumbar* near/3 (disc* near/3 (extru* or degenerat* or displac* or herniat* or prolaps* or sequestered or slipped or protru* or avuls*))):ab

#29 (lumbar* near/3 (disk* near/3 (extru* or degenerat* or displac* or herniat* or prolaps* or sequestered or slipped or protru* or avuls*))):ti OR (lumbar* near/3 (disk* near/3 (extru* or degenerat* or displac* or herniat* or prolaps* or sequestered or slipped or protru* or avuls*))):ab

#30 (lumbar* near/3 (pain* or facet* or (nerve near/2 root*) or osteoarth* or radicul* or stenos* or spondylo* or zygapophys* or injur* or discomfort* or dysfunction* or sore* or herniat*)):ti OR (lumbar* near/3 (pain* or facet* or (nerve near/2 root*) or osteoarth* or radicul* or stenos* or spondylo* or zygapophys* or injur* or discomfort* or dysfunction* or sore* or herniat*)):ab

#31 (lumbo* near/3 (pain* or facet* or (nerve near/2 root*) or osteoarth* or radicul* or stenos* or spondylo* or zygapophys* or injur* or discomfort* or dysfunction* or sore* or herniat*)):ti OR (lumbo* near/3 (pain* or facet* or (nerve near/2 root*) or osteoarth* or radicul* or stenos* or spondylo* or zygapophys* or injur* or discomfort* or dysfunction* or sore* or herniat*)):ab

#32 (back near/3 (ach* or injur* or pain* or sprain* or strain* or disorder*)):ti OR (back near/3 (ach* or injur* or pain* or sprain* or strain* or disorder*)):ab

#33 (backach*):ti OR (backach*):ab

#34 (back-pain*):ti OR (back-pain*):ab

#35 (intervertebral* near/3 (disc* near/3 (extru* or degenerat* or displac* or herniat* or prolaps* or sequestered or slipped or protru* or avuls*))):ti OR (intervertebral* near/3 (disc* near/3 (extru* or degenerat* or displac* or herniat* or prolaps* or sequestered or slipped or protru* or avuls*))):ab

#36 (intervertebral* near/3 (disk* near/3 (extru* or degenerat* or displac* or herniat* or prolaps* or sequestered or slipped or protru* or avuls*))):ti OR (intervertebral* near/3 (disk* near/3 (extru* or degenerat* or displac* or herniat* or prolaps* or sequestered or slipped or protru* or avuls*))):ab

#37 (coccy* near/2 (ach* or injur* or pain* or sprain* or strain*)):ti OR (coccy* near/2 (ach* or injur* or pain* or sprain* or strain*)):ab

#38 ((coccygodyn* or coccalg* or coccygalg*)):ti OR ((coccygodyn* or coccalg* or coccygalg*)):ab

#39 (dorsalg* or lumbago* or lumboischialg*):ti OR (dorsalg* or lumbago* or lumboischialg*):ab

#40 ((piriformis* near/2 syndrome*)):ti OR ((piriformis* near/2 syndrome*)):ab

#41 (sacral* near/3 (pain* or facet* or (nerve near/2 root*) or osteoarth* or radicul* or stenos* or spondylo* or zygapophys* or injur* or discomfort* or dysfunction* or sore* or herniat*)):ti OR (sacral* near/3 (pain* or facet* or (nerve near/2 root*) or osteoarth* or radicul* or stenos* or spondylo* or zygapophys* or injur* or discomfort* or dysfunction* or sore* or herniat*)):ab

#42 (sacro* near/3 (pain* or facet* or (nerve near/2 root*) or osteoarth* or radicul* or stenos* or spondylo* or zygapophys* or injur* or discomfort* or dysfunction* or sore* or herniat*)):ti OR (sacro* near/3 (pain* or facet* or (nerve near/2 root*) or osteoarth* or radicul* or stenos* or spondylo* or zygapophys* or injur* or discomfort* or dysfunction* or sore* or herniat*)):ab

#43 ("si" near/2 (joint* near/3 (pain* or facet* or (nerve near/2 root*) or osteoarth* or radicul* or stenos* or spondylo* or zygapophys* or injur* or discomfort* or dysfunction* or sore* or herniat*))):ti OR ("si" near/2 (joint* near/3 (pain* or facet* or (nerve near/2 root*) or osteoarth* or radicul* or stenos* or spondylo* or zygapophys* or injur* or discomfort* or dysfunction* or sore* or herniat*))):ab

#44 (sacrococcy* near/2 (ach* or injur* or pain* or sprain* or strain*)):ti OR (sacrococcy* near/2 (ach* or injur* or pain* or sprain* or strain*)):ab

#45 (sacrum* near/2 (ach* or injur* or pain* or sprain* or strain*)):ti OR (sacrum* near/2 (ach* or injur* or pain* or sprain* or strain*)):ab

#46 (sciatic*):ti OR (sciatic*):ab ˙

#47 (stenos* near/2 (spine* or spinal* or vertebral*)):ti OR (stenos* near/2 (spine* or spinal* or vertebral*)):ab

#48 ((spine* or spinal*) near/2 osteoarthr*):ti OR ((spine* or spinal*) near/2 osteoarthr*):ab

#49 (spine* near/3 (condition* or diseas* or disabilit* or disorder* or degenerat* or pain* or stenos*)):ti OR (spine* near/3 (condition* or diseas* or disabilit* or disorder* or degenerat* or pain* or stenos*)):ab

#50 (spinal* near/3 (condition* or diseas* or disabilit* or disorder* or degenerat* or pain* or stenos*)):ti OR (spinal* near/3 (condition* or diseas* or disabilit* or disorder* or degenerat* or pain* or stenos*)):ab

#51 (spondylo*):ti OR (spondylo*):ab

#52 (tailbone* near/3 (ach* or injur* or pain* or sprain* or strain*)):ti OR (tailbone* near/3 (ach* or injur* or pain* or sprain* or strain*)):ab

#53 (vertebr* near/3 (ach* or injur* or pain* or sprain* or strain*)):ti OR (vertebr* near/3 (ach* or injur* or pain* or sprain* or strain*)):ab

#54 (poly-radicul* or polyradicul*):ti OR (poly-radicul* or polyradicul*):ab

#55 (neuropath* near/2 (lumbar* or lumbo* or sacral* or sacro* or (low* near/2 back) or low-back* or lower-back* or spine* or spinal* or L1 or L2 or L3 or L4 or L5)):ti OR (neuropath* near/2 (lumbar* or lumbo* or sacral* or sacro* or (low* near/2 back) or low-back* or lower-back* or spine* or spinal* or L1 or L2 or L3 or L4 or L5)):ab

#56 (radiculopath* near/3 (lumbar* or lumbo* or sacral* or sacro* or (low* near/2 back) or low-back* or lower-back* or spine* or spinal* or L1 or L2 or L3 or L4 or L5)):ti OR (radiculopath* near/3 (lumbar* or lumbo* or sacral* or sacro* or (low* near/2 back) or low-back* or lower-back* or spine* or spinal* or L1 or L2 or L3 or L4 or L5)):ab

#57 (radiating* near/3 (lumbar* or lumbo* or sacral* or sacro* or (low* near/2 back) or low-back* or lower-back* or spine* or spinal* or L1 or L2 or L3 or L4 or L5)):ti OR (radiating* near/3 (lumbar* or lumbo* or sacral* or sacro* or (low* near/2 back) or low-back* or lower-back* or spine* or spinal* or L1 or L2 or L3 or L4 or L5)):ab

#58 (radicular* near/3 (lumbar* or lumbo* or sacral* or sacro* or (low* near/2 back) or low-back* or lower-back* or spine* or spinal* or L1 or L2 or L3 or L4 or L5)):ti OR (radicular* near/3 (lumbar* or lumbo* or sacral* or sacro* or (low* near/2 back) or low-back* or lower-back* or spine* or spinal* or L1 or L2 or L3 or L4 or L5)):ab

#59 (lumborum* near/3 (ach* or injur* or pain* or sprain* or strain*)):ti OR (lumborum* near/3 (ach* or injur* or pain* or sprain* or strain*)):ab

#60 (longissimus* near/3 (ach* or injur* or pain* or sprain* or strain*)):ti OR (longissimus* near/3 (ach* or injur* or pain* or sprain* or strain*)):ab

#61 ((erector near/2 spin*) near/3 (ach* or injur* or pain* or sprain* or strain*)):ti OR ((erector near/2 spin*) near/3 (ach* or injur* or pain* or sprain* or strain*)):ab

#62 (synovial* near/2 cyst*):ti OR (synovial* near/2 cyst*):ab

#63 (thoracolumbar* near/3 (pain* or facet* or (nerve* near/2 root*) or osteoarthr* or radicul* or stenos* or spondylo* or zygapohys* or injur* or trauma* or discomfort* or dysfunction* or sore* or herniat*)):ti OR (thoracolumbar* near/3 (pain* or facet* or (nerve* near/2 root*) or osteoarthr* or radicul* or stenos* or spondylo* or zygapohys* or injur* or trauma* or discomfort* or dysfunction* or sore* or herniat*)):ab

#64 (thoraco-lumbar* near/3 (pain* or facet* or (nerve* near/2 root*) or osteoarthr* or radicul* or stenos* or spondylo* or zygapohys* or injur* or trauma* or discomfort* or dysfunction* or sore* or herniat*)):ti OR (thoraco-lumbar* near/3 (pain* or facet* or (nerve* near/2 root*) or osteoarthr* or radicul* or stenos* or spondylo* or zygapohys* or injur* or trauma* or discomfort* or dysfunction* or sore* or herniat*)):ab

#65 (curvatur* near/2 (spine* or spinal*)):ti OR (curvatur* near/2 (spine* or spinal*)):ab

#66 ((pathol* near/2 (lumbar* or (low* near/2 back) or low-back* or (lower* near/2 back) or lower-back* or thoracolumbar* or thoraco-lumbar* or intervertebral* or lumbosacral* or lumbo-sacral* or sacral* or sacro-iliac* or sacroiliac*))):ti OR ((pathol* near/2 (lumbar* or (low* near/2 back) or low-back* or (lower* near/2 back) or lower-back* or thoracolumbar* or thoraco-lumbar* or intervertebral* or lumbosacral* or lumbo-sacral* or sacral* or sacro-iliac* or sacroiliac*))):ab

#67 MeSH descriptor: [Transcutaneous Electric Nerve Stimulation] this term only

#68 MeSH descriptor: [Electric Stimulation Therapy] this term only

#69 MeSH descriptor: [Electric Stimulation] this term only

#70 (ALTENS or TNS or TENMS or C-TENS or A-TENS):ti OR (ALTENS or TNS or TENMS or C-TENS or A-TENS):ab

#71 (trans-cutan* near/3 (electric* or nerve* or stimul* or neurostimul*)):ti OR (trans-cutan* near/3 (electric* or nerve* or stimul* or neurostimul*)):ab

#72 (transdermal* near/3 (electric* or nerve* or stimul* or neurostimul*)):ti OR (transdermal* near/3 (electric* or nerve* or stimul* or neurostimul*)):ab

#73 (trans-dermal* near/3 (electric* or nerve* or stimul* or neurostimul*)):ti OR (trans-dermal* near/3 (electric* or nerve* or stimul* or neurostimul*)):ab

#74 ((peripheral* near/2 (conditioning n2 stimul*))):ti OR ((peripheral* near/2 (conditioning n2 stimul*))):ab

#75 ((microamperage* near/2 stimul*).):ti OR ((microamperage* near/2 stimul*)):ab

#76 ((microamperage* near/2 stimul*)):ti OR ((microamperage* near/2 stimul*)):ab

#77 ((transabdominal n3 neurostimul*)):ti OR ((transabdominal n3 neurostimul*)):ab

#78 ((trans-abdominal n3 neurostimul*)):ti OR ((trans-abdominal n3 neurostimul*)):ab

#79 (electroanalges*):ti OR (electroanalges*):ab

#80 (electro-analges*):ti OR (electro-analges*):ab

#81 (electrotherap*):ti OR (electrotherap*):ab

#82 (electro-therap*):ti OR (electro-therap*):ab

#83 ((electr* near/2 (stimul*))):ti OR ((electr* near/2 (stimul*))):ab

#84 #1 or #2 or #3 or #4 or #5 or #6 or #7 or #8 or #9 or #10 or #11 or #12 or #13 or #14 or #15 or #16 or #17 or #18 or #19 or #20 or #21 or #22 or #23 or #24 or #25 or #26 or #27 or #28 or #29 or #30 or #31 or #32 or #33 or #34 or #35 or #36 or #37 or #38 or #39 or #40 or #41 or #42 or #43 or #44 or #45 or #46 or #47 or #48 or #49 or #50 or #51 or #52 or #53 or #54 or #55 or #56 or #57 or #58 or #59 or #60 or #61 or #62 or #63 or #64 or #65 or #66

#85 #67 or #68 or #69 or #70 or #71 or #72 or #73 or #74 or #75 or #76 or #77 or #78 or #79 or #80 or #81 or #82 or #83

#86 #84 or #85

#87 #84 AND #85 with Cochrane Library publication date Between Jul 2007 and Mar 2022

**E. Database:**  PEDRO Physiotherapy Evidence Database https://pedro.org.au/

**Years of search:** 2007 – current

**Date search run:**  March 9, 2022, **Number of records retrieved:** 88

**Search Strategy:**

- Abstract & Title: (transcutan*) AND (low back pain)
- [OR] Abstract & Title: (electric stimulation) AND (low back pain)
- [OR] Abstract & Title: (electrical stimulation) AND (low back pain)
- [OR] Abstract & Title: (electroanalges*) AND (low back pain)
- [OR] Abstract & Title: (electrotherap*) AND (low back pain)

Limit: Published since 2007

Limit: Method = clinical trial

**F. Database:**  World Health Organization International Clinical Trials Registry Platform (ICTRP)

https://trialsearch.who.int

**Years of search:** July 1, 2007 – March 2022

**Date search run:**  March 9, 2022, **Number of records retrieved:** 174

**Search Strategy:**

[Basic search option]

- low back pain AND transcutaneous electrical

[OR]

- low back pain AND electrical stimulation

Records selected via ‘date of registration’ July 1, 2007- current.

**Online Resource 2.** Description of included randomized controlled trials (RCTs) (n=17 trials, 16 reports)

| **Alizadeh 2009[1]** | |
| --- | --- |
| Methods | **Trial design:** RCT  **Setting:** Iran  **TENS groups:** 1  **Comparison groups:** 1 |
| Participants | **Number of participants:** 16 (T1: 8, C1: 8)  **Mean age, years (SD):** 22 (3)  **Age stratification (60+ years):** no  **Gender (female)**^†^**:** 100%  **Chronic LBP type:** nonspecific (primary)  **Mean chronic LBP duration, years (SD):** NR  **Leg pain:** none  **Population source:** healthcare |
| Interventions | **Intervention:** TENS + NSAIDs (T1)  **Rationale:** NR  **Materials:** TENS unit  **Procedures:** TENS consisted of the placement of four coetaneous electrode pads. All treatments performed in the afternoon. NSAIDs were ibuprofen and diclofenac as prescribed by a physician  **Pulse intensity:** NR  **Pulse frequency:** 10/70 Hz  **Pulse duration:** NR  **Pulse type (burst/continuous):** NR  **Electrode placement:** NR  **Format:** in-person  **Duration:** 30 min, 15 sessions, 5 weeks  **Location:** rehabilitation centre [single centre]  **Provider:** physiotherapist  **Mode of delivery:** independent  **Tailoring:** individually prescribed NSAIDs  **Modifications:** NR  **Adherence:** NR |
| Comparisons | **Comparisons assessed:** TENS + NSAIDs (T1) vs. NSAIDs (C1)  **Intervention:** NSAIDs (C1)  **Procedure:** NSAIDs were ibuprofen and diclofenac as prescribed by a physician  **Materials:** n/a  **Format:** n/a  **Duration:** ‘taken throughout the trial’ (5 weeks)  **Location:** rehabilitation centre  **Provider:** physician  **Mode of delivery:** independent  **Tailoring:** individually prescribed  **Modifications:** NR |
| Outcomes | Function (Modified ODI, 0-45)  **Follow-up:** immediate term (closest to 2 weeks) |
| Risk of bias | High (refer to Appendix 2 for details) |
| **Bloodworth 2004[2]** | |
| Methods | **Trial design:** crossover RCT  **Setting:** United States  **TENS groups:** 4  **Comparison groups:** 2 |
| Participants | **Number of participants:** 11  **Mean age, years (range):** 55 (36-65)  **Age stratification (60+ years):** no  **Gender (female)**^†^**:** 64%  **Chronic LBP type:** LBP with radiculopathy (45% lumbosacral, 27% lumbar, 27% sacral)  **Mean chronic LBP duration, years (SD):** 3.5 years (SD not reported)  **Leg pain:** 100% (radicular)  **Population source:** NR |
| Interventions | **Intervention:** conventional TENS (back) (T1)  **Rationale:** conventional TENS, with a high-frequency periodic stimulus, produces the sensation of a paresthesia or vibration, and it purportedly activates large, myelinated primary afferent fibers that dampen the transmission of small, nonmyelinated nociceptive fibers at the level of the dorsal horn  **Materials:** TENS unit  **Procedures:**  **Pulse intensity:** 78.2 V  **Pulse frequency:** 66.34 Hz  **Pulse duration:** 0.1 ms. time interval between pulses was fixed at 15.07 ms  **Pulse type (burst/continuous):** continuous  **Electrode placement:** low back over the nerve roots ipsilateral to radicular symptoms  **Format:** in-person  **Duration:** 10 min, 1 session  **Location:** hospital [single centre]  **Provider:** NR  **Mode of delivery:** independent  **Tailoring:** none  **Modifications:** none  **Adherence:** 100%  **Intervention:** conventional TENS (leg) (T2)  **Rationale:** conventional TENS, with a high-frequency periodic stimulus, produces the sensation of a paresthesia or vibration, and it purportedly activates large, myelinated primary afferent fibers that dampen the transmission of small, nonmyelinated nociceptive fibers at the level of the dorsal horn  **Materials:** TENS unit  **Procedures:** same pulse parameters as T1  **Pulse intensity:** 78.2 V  **Pulse frequency:** 66.34 Hz  **Pulse duration:** 0.1 ms. time interval between pulses was fixed at 15.07 ms  **Pulse type (burst/continuous):** continuous  **Electrode placement:** posterior thigh over peripheral nerve trunks  **Format:** in-person  **Duration:** 10 min, 1 session  **Location:** hospital [single centre]  **Provider:** NR  **Mode of delivery:** independent  **Tailoring:** none  **Modifications:** none  **Adherence:** 100%  **Intervention:** stochastic resonance TENS (back) (T3)  **Rationale:** stochastic resonance is the idea that noise, in nonlinear systems, like electronic circuits and biological sensory neurons, can amplify or render detectable a weak signal  **Materials:** TENS unit  **Procedures:**  **Pulse intensity:** 78.2 V  **Pulse frequency:** 66.39 Hz (average over 100 second sample)  **Pulse duration:** 0.1 ms; time interval between pulses was randomized using a microprocessor and associated pulse-generating circuitry, operated by a single standard 9-V battery and mounted to the TENS unit; randomly generated 200 pulse interval sequence was repeated throughout the treatment; interval between pulse delivery ranged from 0.3 ms to 30.3 ms  **Pulse type (burst/continuous):** continuous  **Electrode placement:** low back over the nerve roots ipsilateral to radicular symptoms  **Format:** in-person  **Duration:** 10 min, 1 session  **Location:** hospital [single centre]  **Provider:** NR  **Mode of delivery:** independent  **Tailoring:** none  **Modifications:** none  **Adherence:** 100%  **Intervention:** stochastic resonance TENS (leg) (T4)  **Rationale:** stochastic resonance is the idea that noise, in nonlinear systems, like electronic circuits and biological sensory neurons, can amplify or render detectable a weak signal  **Materials:** TENS unit  **Procedures:** same pulse parameters as T3  **Pulse intensity:** 78.2 V  **Pulse frequency:** 66.39 Hz (average over 100 second sample)  **Pulse duration:** 0.1 ms; time interval between pulses was randomized using a microprocessor and associated pulse-generating circuitry, operated by a single standard 9-V battery and mounted to the TENS unit; randomly generated 200 pulse interval sequence was repeated throughout the treatment; interval between pulse delivery ranged from 0.3 ms to 30.3 ms  **Pulse type (burst/continuous):** continuous  **Electrode placement:** posterior thigh over peripheral nerve trunks  **Format:** in-person  **Duration:** 10 min, 1 session  **Location:** hospital [single centre]  **Provider:** NR  **Mode of delivery:** independent  **Tailoring:** none  **Modifications:** none  **Adherence:** 100% |
| Comparisons | **Comparisons assessed:**   1. Conventional TENS (back) (T1) vs. sham TENS (back) (C1) 2. Conventional TENS (leg) (T2) vs. sham TENS (leg) (C2) 3. Stochastic Resonance TENS (back) (T3) vs. sham TENS (back) (C1) 4. Stochastic Resonance TENS (leg) (T4) vs. sham TENS (leg) (C2)   **Intervention:** sham TENS (back) (C1)  **Materials:** portable standard sized TENS unit  **Procedures:** TENS unit switched off; electrodes were placed on the low back over the nerve roots ipsilateral to radicular symptoms  **Format:** in-person  **Duration:** 10 min, 1 session  **Location:** hospital [single centre]  **Provider:** NR  **Mode of delivery:** independent  **Tailoring:** none  **Modifications:** none  **Intervention:** sham TENS (leg) (C2)  **Materials:** portable standard sized TENS unit  **Procedures:** TENS unit switched off; electrodes were placed on posterior thigh over peripheral nerve trunks  **Format:** in-person  **Duration:** 10 min, 1 session  **Location:** hospital [single centre]  **Provider:** NR  **Mode of delivery:** independent  **Tailoring:** none  **Modifications:** none |
| Outcomes | Pain (VAS, 0-10; MPQ, 0-78)  **Follow-up:** immediate term (closest to 2 weeks) |
| Risk of bias | High (refer to Appendix 2 for details) |
| Notes | 1. Results not included in meta-analysis because trial authors only provided p-values for pain (MPQ) for relevant comparisons (stochastic resonance TENS back/leg vs. sham TENS, p=0.096; conventional TENS back/leg vs. sham TENS, p=0.519). 2. MPQ: No description of how the body mannequin was scored (Part 1) and what the range of possible scores is. |
| **Cheing 1999[3]** | |
| Methods | **Trial design:** RCT  **Setting:** Canada, China  **TENS groups:** 1  **Comparison groups:** 1 |
| Participants | **Number of participants:** 30 (T1=15, C1=15)  **Mean age, years (SD):** T1=34.7 (9.1), C1=28.2 (7.2)  **Age stratification (60+ years):** no  **Gender (female)**^†^**:** T1=27%, C1=33%  **Chronic LBP type:** NR (assumed nonspecific primary)  **Mean chronic LBP duration, years (SD):** T1=6.3 (5.7), C1=5.7 (4.3)  **Leg pain:** none (excluded people with consistent sciatica symptoms)  **Population source:** NR |
| Interventions | **Intervention:** TENS (continuous) (T1)  **Rationale:** stimulation of large diameter afferent fibres reducing transmission of pain signals through the small nociceptive afferent fibres, inhibiting pain discrimination and perception  **Materials:** dual channel portable TENS unit (Staodyn MAXIMA III)  **Procedures:**  **Pulse intensity:** approximately 2 to 3 times the sensory threshold  **Pulse frequency:** 80 Hz  **Pulse duration:** 140 μs  **Pulse type (burst/continuous):** none  **Electrode placement:** paraspinally at the lumbosacral region  **Format:** in-person  **Duration:** 60 min, 1 session  **Location:** laboratory setting [single centre]  **Provider:** NR  **Mode of delivery:** independent  **Tailoring:** none  **Modifications:** none  **Adherence:** 100% |
| Comparisons | **Comparisons assessed:** TENS (T1) vs. sham TENS (C1)  **Intervention:** sham TENS (C1)  **Materials:** dual channel portable TENS unit (Staodyn MAXIMA III) (disconnected)  **Procedures:** disconnected internal circuit. Program and electrode placement same as T1  **Format:** in-person  **Duration:** 60 min, 1 session  **Location:** laboratory setting [single centre]  **Provider:** NR  **Mode of delivery:** independent  **Tailoring:** none  **Modifications:** none |
| Outcomes | Pain (VAS, 0-100)  **Follow-up:** immediate term (closest to 2 weeks) |
| Risk of bias | High (refer to Appendix 2 for details) |
| **de Alencar Caldas 2021[4]** | |
| Methods | **Trial design:** RCT  **Setting:** Brazil  **TENS groups:** 2  **Comparison groups:** 2 |
| Participants | **Number of participants:** 56 (T1: 14, T2: 14, C1: 14, C2: 14)  **Mean age, years (SD):** T1: 29.6 (9.5), T2: 28.5 (8.7), C1: 28.4 (9.6), C2: 29 (8.1)  **Age stratification (60+ years):** no  **Gender (female)**^†^**:** 54%  **Chronic LBP type:** nonspecific (primary)  **Mean chronic LBP duration, years (SD):** NR  **Leg pain:** none (radicular pain excluded)  **Population source:** healthcare |
| Interventions | **Intervention:** burst TENS (T1)  **Rationale:** NR  **Materials:** TENS unit (KLD sonophasys device)  **Procedures:**  **Pulse intensity:** maximum intensity to generate visible muscle contraction without generating pain/discomfort due to stimulation  **Pulse frequency:** 4 Hz  **Pulse duration:** 240 μsec  **Pulse type (burst/continuous):** none  **Electrode placement:** paravertebral (L3 and L5)  **Format:** in-person  **Duration:** 30 min, 10 sessions, 4 weeks  **Location:** university hospital [single centre]  **Provider:** NR  **Mode of delivery:** independent  **Tailoring:** none  **Modifications:** none  **Adherence:** NR  **Intervention:** burst TENS + cryotherapy (T2)  **Rationale:** combined use of the two modalities is associated with potentiation  of the physiological effects that provide analgesia  **Materials:** TENS unit (KLD sonophasys device), cryotherapy device (Thermomatic TH3)  **Procedures:** same program, pulse parameters and electrode placement as T1; same cryotherapy as C2  **Pulse intensity:** maximum intensity to generate visible muscle contraction without generating pain/discomfort due to stimulation  **Pulse frequency:** 4 Hz  **Pulse duration:** 240 μsec  **Pulse type (burst/continuous):** none  **Electrode placement:** paravertebral (L3 and L5)  **Format:** in-person  **Duration:** 30 min, 10 sessions, 4 weeks  **Location:** university hospital [single centre]  **Provider:** NR  **Mode of delivery:** independent  **Tailoring:** none  **Modifications:** none  **Adherence:** NR |
| Comparisons | **Comparisons assessed:**   1. Burst TENS (T1) vs. sham TENS (C1) 2. Burst TENS + cryotherapy (T2) vs. cryotherapy (C2)   **Intervention:** sham TENS (C1)  **Materials:** TENS unit (KLD sonophasys device)  **Procedures:** same program and electrode placement as T1 except minimal therapeutic dose of burst TENS  **Format:** in-person  **Duration:** 30 min, 10 sessions, 4 weeks  **Location:** university hospital [single centre]  **Provider:** NR  **Mode of delivery:** independent  **Tailoring:** none  **Modifications:** none  **Intervention:** cryotherapy (C2)  **Materials:** cryotherapy device (Thermomatic TH3)  **Procedures:** using ice from machine (Thermomatic TH3) and a plastic bag, each containing 700g  **Format:** in-person  **Duration:** 30 min, 10 sessions, 4 weeks  **Location:** university hospital [single centre]  **Provider:** NR  **Mode of delivery:** independent  **Tailoring:** none  **Modifications:** none |
| Outcomes | Pain (VAS, 0-10); function (RMDQ, 0-24); health-related quality of life (SF-36, 0-100)  **Follow-up:** immediate term (closest to 2 weeks) |
| Risk of bias | High (refer to Appendix 2 for details) |
| Notes | Minimal therapeutic dose used in sham TENS: trial authors referenced a transient TENS device that “completely blinds investigators to treatment and does not reduce pain, thereby providing a true placebo treatment.” (Rakel 2010, p. 230) |
| **Depaoli Lemos 2021[5]** | |
| Methods | **Trial design:** RCT  **Setting:** Brazil  **TENS groups:** 1  **Comparison groups:** 1 |
| Participants | **Number of participants:** 32 (T1: 16, C1: 16)  **Mean age, years (SD):** T1: 52.5 (12.4), C1: 50.8 (13)  **Age stratification (60+ years):** no  **Gender (female)**^†^**:** T1**:** 81%, C1: 69%  **Chronic LBP type:** nonspecific (primary)  **Mean chronic LBP duration, years (SD):** T1: 5.6 (3.7), C1: 5 (3.6)  **Leg pain:** NR  **Population source:** healthcare |
| Interventions | **Intervention:** TENS + kinesiotherapy (T1)  **Rationale:**  **Materials:** portable two-channel transcutaneous electrostimulator (brand Ibramed®, model Neurodyn; ANVISA registration number 10360310012)  **Procedures:**  **Pulse intensity:** according to tolerance  **Pulse frequency:** 10 Hz  **Pulse duration:** 250 μs  **Pulse type (burst/continuous):** none  **Electrode placement:** lumbar paravertebral musculature bilaterally  **Format:** in-person  **Duration:** 20 min, 12 sessions, 4 weeks  **Location:** NR  **Provider:** NR  **Mode of delivery:** independent  **Tailoring:** intensity according to tolerance  **Modifications:**  **Adherence:** NR |
| Comparisons | **Comparisons assessed:** TENS + kinesiotherapy (T1) vs. kinesiotherapy (C1)  **Intervention:** kinesiotherapy (C1)  **Materials:** NR  **Procedures:** three 20-second sets of stretches for posterior muscle chain; 10 repetitions of strengthening exercises (supine bridge, single leg supine bridge, side bridge, prone plank) for core stabilizing muscles (held for 6” in weeks 1 and 2, 8” in week 3, 10” in week 4)  **Format:** NR  **Duration:** 12 sessions, 4 weeks  **Location:** NR  **Provider:** NR  **Mode of delivery:** independent  **Tailoring:** NR  **Modifications:** NR |
| Outcomes | Pain (VAS, 0-10); function (RMDQ, 0-24)  **Follow-up:** immediate term (closest to 2 weeks), short term (closest to 3 months) |
| Risk of bias | High (refer to Appendix 2 for details) |
| Notes | VAS scale range not described. Assumed 0-10 range based on results. |
| **Dias 2021[6]** | |
| Methods | **Trial design:** RCT  **Setting:** Brazil  **TENS groups:** 2  **Comparison groups:** 1 |
| Participants | **Number of participants:** 105 (T1: 35, T2: 35, C1: 35)  **Mean age, years (SD):** 39.6 (15.3)  **Age stratification (60+ years):** no  **Gender (female)**^†^**:** 64%  **Chronic LBP type:** nonspecific (primary)  **Mean chronic LBP duration, years (SD):** NR  **Leg pain:** NR  **Population source:** healthcare |
| Interventions | **Intervention:** TENS (GT100Hz) (T1)  **Rationale:** produces inhibitory effects on nerve transmissions of nociceptive fibers  **Materials:** TENS unit (MEDMART)  **Procedures:**  **Pulse intensity:** sensory level  **Pulse frequency:** 100 Hz  **Pulse duration:** 100 μs  **Pulse type (burst/continuous):** none  **Electrode placement:** lumbar paravertebral  **Format:** individual  **Duration:** 30 min, 1 session  **Location:** NR  **Provider:** physical therapist  **Mode of delivery:** in-person  **Tailoring:** pulse intensity set to sensory level  **Modifications:** none  **Adherence:** 100%  **Intervention:** TENS (GT2Hz) (T2)  **Rationale:** depolarizes the fast pain fibers capable of activation of descending analgesia  **Materials:** TENS unit (MEDMART)  **Procedures:**  **Pulse intensity:** motor level  **Pulse frequency:** 2 Hz  **Pulse duration:** 100 μs  **Pulse type (burst/continuous):** none  **Electrode placement:** lumbar paravertebral  **Format:** in-person  **Duration:** 30 min, 1 session  **Location:** NR  **Provider:** physical therapist  **Mode of delivery:** independent  **Tailoring:** pulse intensity set to motor level  **Modifications:** none  **Adherence:** 100% |
| Comparisons | **Comparisons assessed:**   1. TENS (GT100Hz) (T1) vs. sham TENS (C1) 2. TENS (GT2Hz) (T2) vs. sham TENS (C1)   **Intervention:** sham TENS (C1)  **Materials:** TENS unit (MEDMART)  **Procedures:** same program duration and electrode placement as T1 and T2 except no current  **Format:** in-person  **Duration:** 30 min, 1 session  **Location:** NR  **Provider:** physical therapist  **Mode of delivery:** independent  **Tailoring:** none  **Modifications:** none |
| Outcomes | Pain (NRS, 0-10; MPQ)  **Follow-up:** immediate term (closest to 2 weeks) |
| Risk of bias | High (refer to Appendix 2 for details) |
| Notes | Results from NRS were used in meta-analysis |
| **Elserty 2016[7]** | |
| Methods | **Trial design:** RCT  **Setting:** Egypt  **TENS groups:** 2  **Comparison groups:** 1 |
| Participants | **Number of participants:** 120 (T1: 40, T2: 40, C1: 40)  **Mean age, years (SD):** T1: 36, T2: 35, C1: 35 (SD not reported)  **Age stratification (60+ years):** no  **Gender (female)**^†^**:** 69%  **Chronic LBP type:** nonspecific (primary)  **Mean chronic LBP duration, years (SD):** NR  **Leg pain:** none (excluded radiating pain)  **Population source:** healthcare |
| Interventions | **Intervention:** TENS (fixed amplitude) + exercise (T1)  **Rationale:** NR  **Materials:** TENS unit  **Procedures:**  **Pulse intensity:** according to patient report of comfortable tingling sensation  **Pulse frequency:** 120 Hz  **Pulse duration:** 100 ms  **Pulse type (burst/continuous):** none  **Electrode placement:** level of the lumbar spine bilaterally  **Format:** in-person  **Duration:** 40 min, 12 sessions, 4 weeks  **Location:** hospital [single centre]  **Provider:** NR  **Mode of delivery:** independent  **Tailoring:** the pulse amplitude gradually increased until the patient reported a comfortable tingling sensation  **Modifications:** NR  **Adherence:** unclear  **Intervention:** TENS (adjusted amplitude) + exercise (T2)  **Rationale:** NR  **Materials:** TENS unit  **Procedures:**  **Pulse intensity:** according to patient report of comfortable tingling sensation, adjusted every 5 minutes (increased again until patient reported a comfortable tingling sensation)  **Pulse frequency:** 120 Hz  **Pulse duration:** 100 ms  **Pulse type (burst/continuous):** none  **Electrode placement:** level of the lumbar spine bilaterally  **Format:** in-person  **Duration:** 40 min, 12 sessions, 4 weeks  **Location:** hospital [single centre]  **Provider:** NR  **Mode of delivery:** independent  **Tailoring:** the pulse amplitude gradually increased until the patient reported a comfortable tingling sensation  **Modifications:** NR  **Adherence:** unclear |
| Comparisons | **Comparisons assessed:**   1. TENS (fixed pulse) + exercise (T1) vs. exercise (C1) 2. TENS (adjusted pulse) + exercise (T2) vs. exercise (C1)   **Intervention:** exercise (C1)  **Materials:** n/a  **Procedures:** bridging exercise and quadruped progression exercises  **Format:** NR  **Duration:** NR  **Location:** NR  **Provider:** NR  **Mode of delivery:** NR  **Tailoring:** NR  **Modifications:** NR |
| Outcomes | Pain (VAS, 0-10); function (ODI 0-100)  **Follow-up:** immediate term (closest to 2 weeks) |
| Risk of bias | High (refer to Appendix 2 for details) |
| **Ezema 2022[8]** | |
| Methods | **Trial design:** RCT  **Setting:** Nigeria  **TENS groups:** 1  **Comparison groups:** 1 |
| Participants | **Number of participants:** 70 (T1: 35, C1: 35)  **Mean age, years (SD):** 53.3 (5.1)  **Age stratification (60+ years):** no  **Gender (female)**^†^**:** T1: 13%, C1: 25%  **Chronic LBP type:** lumbar spondylosis  **Mean chronic LBP duration, months (SD):** 13.1 (9)  **Leg pain:** none  **Population source:** healthcare |
| Interventions | **Intervention:** bi-channel burst modulation TENS (T1)  **Rationale:** post-stimulation analgesic effect  **Materials:** TENS unit (MediHightec Medical-MH6200 Combo; made in Taiwan)  **Procedures:**  **Pulse intensity:** 40 mA (maximum tolerable intensity)  **Pulse frequency:** 100 Hz, 2 Hz burst rate  **Pulse duration:** 150 μs burst width at 10 μs per step  **Pulse type (burst/continuous):** burst  **Electrode placement:** lumbar paraspinal bilaterally  **Format:** in-person  **Duration:** 30 min, 1 session  **Location:** hospital [multicenter]  **Provider:** physiotherapist  **Mode of delivery:** independent  **Tailoring:** maximum tolerable intensity varied by participant  **Modifications:** NR  **Adherence:** 100% |
| Comparisons | **Comparisons assessed:** TENS (T1) vs. sham TENS (T2)  **Intervention:** sham TENS (C1)  **Materials:** TENS unit (MediHightec Medical-MH6200 Combo; made in Taiwan)  **Procedures:** all parameters set at zero except the timer (after 30 min session). Electrode placement: lumbar paraspinal bilaterally  **Format:** in-person  **Duration:** 30 min, 1 session  **Location:** hospital [multicenter]  **Provider:** physiotherapist  **Mode of delivery:** independent  **Tailoring:** none  **Modifications:** NR |
| Outcomes | Pain (NRS, 0-10)  **Follow-up:** immediate term (closest to 2 weeks) |
| Risk of bias | Moderate (refer to Appendix 2 for details) |
| **Jamison 2019[9]** | |
| Methods | **Trial design:** RCT  **Setting:** United States  **TENS groups:** 1  **Comparison groups:** 1 |
| Participants | **Number of participants:** 68 (T1: 35, C1: 33)  **Mean age, years (SD):** 46.2 (12.7)  **Age stratification (60+ years):** no  **Gender (female)**^†^**:** 60%  **Chronic LBP type:** diagnosed axial musculoskeletal or discogenic back pain with or without radiculopathy based on hospital record review  **Mean chronic LBP duration, years (SD):** 13.2 (11.9)  **Leg pain:** mixed with and without leg pain (mixed radicular and non-radicular)  **Population source:** healthcare |
| Interventions | **Intervention:** high-frequency TENS (home program) (T1)  **Rationale:** diffuse noxious inhibitory control (DNIC) response, lateral inhibition within the spinal cord, stimulation of cortical coding areas involved with pain in the brain, and general distraction and selective attention  **Materials:** TENS unit (Quell, NeuroMetrix, Inc., Waltham, MA, USA)  **Procedures:** pulse parameters: intensity: NR; frequency: NR; duration: NR. Electrode placement:  **Pulse intensity:** NR  **Pulse frequency:** NR  **Pulse duration:** NR  **Pulse type (burst/continuous):** none  **Electrode placement:** around the calf of one leg  **Format:** home-based  **Duration:** encouraged to wear device 2 hours daily, 90 sessions, 12 weeks  **Location:** home-based  **Provider:** self-administered  **Mode of delivery:** independent  **Tailoring:** NR  **Modifications:** NR  **Adherence:** NR |
| Comparisons | **Comparisons assessed:** high-frequency TENS (home program) (T1) vs. no treatment (C1)  **Intervention:** no treatment (C1)  **Materials:** n/a  **Procedures:** all participants asked to use the smartphone pain app to monitor their daily assessments (as T1)  **Format:** n/a  **Duration:**  **Location:** n/a  **Provider:** n/a  **Mode of delivery:** n/a  **Tailoring:** n/a  **Modifications:** n/a |
| Outcomes | Pain (BPI, 0-10), function (PDI, 0-70), functional limitations (NRS, 0-10), depression (HADS, 0-21), adverse events (recorded only when reported by participants), catastrophizing (PCS, 0-52)  **Follow-up:** immediate term (closest to 2 weeks) |
| Risk of bias | Moderate (refer to Appendix 2 for details) |
| Notes | 1. ‘Average’ pain measured by BPI data was used in meta-analysis. The intensity of pain in general, at its worst, at its least, and pain “right now” over the past 24 hours were also reported as measured by BPI. |
| **Kibar 2020[10]** | |
| Methods | **Trial design:** RCT  **Setting:** Turkey  **TENS groups:** 2  **Comparison groups:** 2 |
| Participants | **Number of participants:** 134 (T1: 34, T2: 33, C1: 34, C2: 33)  **Mean age, years (SD):** T1: 53.6 (13.7), T2: 53.5 (13.7), C1: 53.7 (11.6), C2: 59 (7.9)  **Age stratification (60+ years):** no  **Gender (female)**^†^**:** T1: 68%, T2: 72%, C1: 77%, C2: 67%  **Chronic LBP type:** LBP with or without radicular pain  **Mean chronic LBP duration, years (SD):** NR  **Leg pain:** Mixed with and without leg pain (radicular)  **Population source:** healthcare |
| Interventions | **Intervention:** TENS (continuous and burst) (T1)  **Rationale:** the gate-control theory suggests that TENS may prevent pain pathways in the spinal cord dorsal horn from working. Additionally, it is thought that stimulating the nervous system via transcutaneous electrodes modifies pain perception and induces release of endogenous analgesic substances such as endorphins  **Materials:** TENS unit (Intelect® Legend Stimulator (DJO, LLC, Vista, CA, USA)  **Procedures:** order of treatment at each session was conventional, burst, and conventional. All intervention and comparison groups received hot pack on lower back (20 minutes) and exercise instructions (pelvic tilt, hamstring stretching, quadruped cat and camel exercises)  **Pulse intensity:** continuous 0-60 mA, burst  **Pulse frequency:** 100 Hz, burst 2 Hz every 3 seconds  **Pulse duration:** continuous 50-100 μs, burst 100-400 μs  **Pulse type (burst/continuous):** combination of conventional (continuous stimulation) and burst mode  **Electrode placement:** lumbar and sacral paravertebral level  **Format:** in-person  **Duration:** 30 min, 15 sessions, 3 weeks  **Location:** NR  **Provider:** physiotherapist  **Mode of delivery:** independent  **Tailoring:** none  **Modifications:** NR  **Adherence:** NR  **Intervention:** TENS + interferential therapy (IFT) (T2)  **Rationale:** the gate-control theory suggests that TENS may prevent pain pathways in the spinal cord dorsal horn from working. Additionally, it is thought that stimulating the nervous system via transcutaneous electrodes modifies pain perception and induces release of endogenous analgesic substances such as endorphins. IFT also affects the tissues through the gate-control theory, but it works by clearing pain-inducing chemicals from the affected area via increased blood flow  **Materials:** TENS and IFC units (Intelect® Legend Stimulator (DJO, LLC, Vista, CA, USA)  **Procedures:** all elements of intervention same as T1, except 30 minutes of TENS followed by 30 minutes IFT. IFT intervention same as C2. All intervention and comparison groups received hot pack on lower back (20 minutes) and exercise instructions (pelvic tilt, hamstring stretching, quadruped cat and camel exercises)  **Pulse intensity:** continuous 0-60 mA, burst  **Pulse frequency:** 100 Hz, burst 2 Hz every 3 seconds  **Pulse duration:** continuous 50-100 μs, burst 100-400 μs  **Pulse type (burst/continuous):** combination of conventional (continuous stimulation) and burst mode  **Electrode placement:** lumbar and sacral paravertebral level  **Format:** in-person  **Duration:** 60 minutes, 15 sessions, 3 weeks  **Location:** NR  **Provider:** physiotherapist  **Mode of delivery:** independent  **Tailoring:** none  **Modifications:** NR  **Adherence:** NR |
| Comparisons | **Comparisons assessed:**   1. TENS (T1) vs. sham TENS (C1) 2. TENS + IFT (T2) vs. IFT (C2)   **Intervention:** sham TENS (C1)  **Materials:** TENS unit (Intelect® Legend Stimulator (DJO, LLC, Vista, CA, USA)  **Procedures:** no current; lights on device. All intervention and comparison groups received hot pack on lower back (20 minutes) and exercise instructions (pelvic tilt, hamstring stretching, quadruped cat and camel exercises)  **Format:** in-person  **Duration:** 30 minutes, 15 sessions, 3 weeks  **Location:** NR  **Provider:** physiotherapist  **Mode of delivery:** independent  **Tailoring:** none  **Modifications:** NR  **Intervention:** IFT (C2)  **Materials:** IFC unit (Intelect® Legend Stimulator (DJO, LLC, Vista, CA, USA)  **Procedures:** true IFT with a four-electrode arrangement therapy included a rectangular alternating waveform with carrier frequencies of 4000 and 4100 Hz for 30 minutes; beat frequency = 100 Hz. All intervention and comparison groups received hot pack on lower back (20 minutes) and exercise instructions (pelvic tilt, hamstring stretching, quadruped cat and camel exercises)  **Format:** in-person  **Duration:** 30 minutes, 15 sessions, 3 weeks  **Location:** NR  **Provider:** physiotherapist  **Mode of delivery:** independent  **Tailoring:** none  **Modifications:** NR |
| Outcomes | Pain during activity, rest, at night (VAS, 0-10); function (RMDQ, 0-24)  **Follow-up:** immediate term (closest to 2 weeks) |
| Risk of bias | High (refer to Appendix 2 for details) |
| Notes | 1. Reported median (min-max); converted to MD (SD) for pooled MD in meta-analysis 2. Not included in meta-analysis since it increased heterogeneity and resulted in I^2^ >75% when included 3. Only VAS at rest was used in meta-analysis (values are similar for VAS during activity and at night) |
| **Kofotolis 2008[11]** | |
| Methods | **Trial design:** RCT  **Setting:** Greece  **TENS groups:** 2  **Comparison groups:** 2 |
| Participants | **Number of participants:** 92 (T1: 23, T2: 23, C1: 23, C2: 23)  **Mean age, years (SD):** T1: 37.5 (8.6), T2: 41.2 (5), C1: 41 (5.5), C2: 42.2 (7.8)  **Age stratification (60+ years):** no  **Gender (female)**^†^**:** 100%  **Chronic LBP type:** nonspecific (primary)  **Mean chronic LBP duration, weeks (SD):** T1: 34.7 (8.8), T2: 34.1 (8.5), C1: 30.6 (6.6), C2: 36.3 (8.2)  **Leg pain:** none  **Population source:** healthcare |
| Interventions | **Intervention:** TENS + rhythmic stabilization (T1)  **Rationale:** rhythmic stabilization is a proprioceptive neuromuscular facilitation technique employing isometric contraction of antagonistic patterns and results in co-contraction of the antagonists  **Materials:** TENS unit  **Procedures:** 20 minutes of TENS followed by 5 minutes of rest; then 20 minutes of rhythmic stabilization. Rhythmic stabilization consisted of alternating (trunk flexion–extension) isometric contractions against resistance for 10 seconds, no motions intended  **Pulse intensity:** strong but comfortable  **Pulse frequency:** 4 Hz  **Pulse duration:** 200 ms  **Pulse type (burst/continuous):** none  **Electrode placement:** fascia thoracolumbaliis and approximately 10cm proximal to this, along the midline of the muscle (i.e., directly over the site of pain)  **Format:** in-person  **Duration:** 45 min, 20 sessions, 4 weeks  **Location:** NR  **Provider:** physical therapist  **Mode of delivery:** independent  **Tailoring:** pulse intensity of TENS varied by participant  **Modifications:** NR  **Adherence:** 91.3%  **Intervention:** TENS (T2)  **Rationale:** NR  **Materials:** TENS unit  **Procedures:** same as T1 (without rhythmic stabilization)  **Pulse intensity:** strong but comfortable  **Pulse frequency:** 4 Hz  **Pulse duration:** 200 ms  **Pulse type (burst/continuous):** none  **Electrode placement:** fascia thoracolumbaliis and approximately 10cm proximal to this, along the midline of the muscle (i.e., directly over the site of pain)  **Format:** in-person  **Duration:** 25 min, 20 sessions, 4 weeks  **Location:** NR  **Provider:** physical therapist  **Mode of delivery:** independent  **Tailoring:** pulse intensity of TENS varied by participant  **Modifications:** NR  **Adherence:** 100% |
| Comparisons | **Comparisons assessed:**   1. TENS + rhythmic stabilization (T1) vs. rhythmic stabilization (C1) 2. TENS (T2) vs. sham TENS (C2)   **Intervention:** rhythmic stabilization (C1)  **Materials:** NR  **Procedures:** PNF technique employing isometric contraction of antagonistic patterns, resulting in co-contraction of the antagonists. Program: alternating (trunk flexion-extension) isometric contractions against resistance for 10 seconds, no motions intended; 3 sets of 15 repetitions at maximal resistance by provider; resting intervals of 30 seconds and 60 seconds were provided after completion of 15 repetitions for each pattern and between sets, respectively  **Format:** in-person  **Duration:** 20 min, 20 sessions, 4 weeks  **Location:** NR  **Provider:** physical therapist  **Mode of delivery:** independent  **Tailoring:** intensity progressed through 4-week period based on mobility progress of participants  **Modifications:** NR  **Intervention:** sham TENS (C2)  **Materials:** TENS unit  **Procedures:** placebo stimulation at the same sites for the same duration and period as the TENS group, using placebo units identical to the real TENS units in appearance, with the indicator lamp lit up when being switched on (internal circuit disconnected)  **Format:** in-person  **Duration:** 25 min, 20 sessions, 4 weeks  **Location:** NR  **Provider:** physical therapist  **Mode of delivery:** independent  **Tailoring:** NR  **Modifications:** NR |
| Outcomes | Pain (Borg Scale, 0-10); function (ODI, 0-50)  **Follow-up:** short term (closest to 3 months) |
| Risk of bias | High (refer to Appendix 2 for details) |
| **Petrofsky 2020[12]** | |
| Methods | **Trial design:** RCT  **Setting:** United States  **TENS groups:** 4  **Comparison groups:** 2 |
| Participants | **Number of participants:** 90 (participants per group not reported)  **Mean age, years (SD):** T1: 47.3 (13.3), T2: 41.9 (12.7), T3: 38.4 (13.1), T4: 47.9 (13.4), C1: 43.3 (11), C2: 39.7 (12.1)  **Age stratification (60+ years):** no  **Gender (female)**^†^**:** NR  **Chronic LBP type:** nonspecific (primary)  **Mean chronic LBP duration, years (SD):** NR  **Leg pain:** none  **Population source:** NR |
| Interventions | **Intervention:** continuous TENS + sham heat (T1)  **Rationale:** inhibiting purine receptors in the peripheral pain nociceptor pathways through voltage-gated calcium channels  **Materials:** TENS unit (Challenge 8000a powered muscle stimulator (MPTS, Tustin, CA, USA)  **Procedures:**  **Pulse intensity:** 11.6 +/- 1.2 mA  **Pulse frequency:** NR  **Pulse duration:** NR  **Pulse type (burst/continuous):** none  **Electrode placement:** levels L1 – S1  **Format:** in-person  **Duration:** 240 min (4 hours), 1 session  **Location:** laboratory setting  **Provider:** physical therapist  **Mode of delivery:** independent  **Tailoring:** intensity of TENS varied by patient  **Modifications:** NR  **Adherence:** 100%  **Intervention:** continuous TENS + low-level continuous heat (LLCH) (T2)  **Rationale:** inhibiting purine receptors in the peripheral pain nociceptor pathways through voltage-gated calcium channels. These channels, the TRPV1 and TPRV4 (transient receptor potential vanilloid type 1 and 4) receptors on sensory neurons, are sensitive to hot and warm temperatures, respectively. LLCH increases elasticity of tissues  **Materials:** TENS unit (Challenge 8000a powered muscle stimulator (MPTS, Tustin, CA, USA)  **Procedures:** Same program duration, pulse parameters and electrode placement as T1 along with dry heat wrap placed around low back  **Pulse intensity:** 11.6 +/- 1.2 mA  **Pulse frequency:** NR  **Pulse duration:** NR  **Pulse type (burst/continuous):** none  **Electrode placement:** levels L1 – S1  **Format:** in-person  **Duration:** 240 min (4 hours), 1 session  **Location:** laboratory setting  **Provider:** physical therapist  **Mode of delivery:** independent  **Tailoring:** intensity of TENS varied by patient  **Modifications:** NR  **Adherence:** 100%  **Intervention:** TENS last 15 min + LLCH (T3)  **Rationale:** LLCH increases elasticity of tissues  **Materials:** TENS unit (Challenge 8000a powered muscle stimulator (MPTS, Tustin, CA, USA), dry heat wrap (ThermaCare, Pfizer Consumer Healthcare, Richmond, VA)  **Procedures:** same pulse intensity and electrode placement as T1. 15 minutes of TENS stimulation at the end of every hour in conjunction with 4 hours of continuous heat delivered using a dry heat wrap placed around the lower back  **Pulse intensity:** 11.6 +/- 1.2 mA  **Pulse frequency:** NR  **Pulse duration:** NR  **Pulse type (burst/continuous):** none  **Electrode placement:** levels L1 – S1  **Format:** in-person  **Duration:** 240 min (4 hours), 1 session  **Location:** laboratory setting  **Provider:** physical therapist  **Mode of delivery:** independent  **Tailoring:** intensity of TENS varied by patient  **Modifications:** NR  **Adherence:** 100%  **Intervention:** TENS last 15 min + sham heat (T4)  **Rationale:** inhibiting purine receptors in the peripheral pain nociceptor pathways through voltage-gated calcium channels  **Materials:** TENS unit (Challenge 8000a powered muscle stimulator (MPTS, Tustin, CA, USA)  **Procedures:** same pulse intensity and electrode placement as T1. 15 minutes of TENS stimulation at the end of every hour in conjunction with 4 hours of sham heat and stimulation  **Pulse intensity:** 11.6 +/- 1.2 mA  **Pulse frequency:** NR  **Pulse duration:** NR  **Pulse type (burst/continuous):** none  **Electrode placement:** levels L1 – S1  **Format:** in-person  **Duration:** 240 min (4 hours), 1 session  **Location:** laboratory setting  **Provider:** physical therapist  **Mode of delivery:** independent  **Tailoring:** intensity of TENS varied by patient  **Modifications:** NR  **Adherence:** 100% |
| Comparisons | **Comparisons assessed:**   1. Continuous TENS + sham heat (T1) vs. sham heat (C1) 2. Continuous TENS + LLCH (T2) vs. LLCH (C2) 3. TENS last 15 min + LLCH (T3) vs. LLCH (C2) 4. TENS last 15 min + sham heat (T4) vs. sham heat (C1)   **Intervention:** sham heat (C1)  **Materials:** NR  **Procedures:** 4 hours of sham heat and sham stimulation (electrodes placed same as T1, stimulation turned off after threshold intensity determined)  **Format:** in-person  **Duration:** 240 min (4 hours), 1 session  **Location:** laboratory setting  **Provider:** physical therapist  **Mode of delivery:** independent  **Tailoring:** NR  **Modifications:** NR  **Intervention:** low-level continuous heat (LLCH) (C2)  **Materials:** dry heat wrap (ThermaCare, Pfizer Consumer Healthcare, Richmond, VA)  **Procedures:** dry heat wrap placed around low back for 4 hours  **Format:** in-person  **Duration:** 240 min (4 hours), 1 session  **Location:** laboratory setting  **Provider:** physical therapist  **Mode of delivery:** independent  **Tailoring:** NR  **Modifications:** NR |
| Outcomes | Pain (VAS, 0-100)  **Follow-up:** immediate term (closest to 2 weeks) |
| Risk of bias | High (refer to Appendix 2 for details) |
| Notes | 1. Number per group not reported (assumed 15 per group) 2. VAS converted to 0-10 scale for pooled MD in meta-analysis |
| **Shimoji 2007[13]** (11253) | |
| Methods | **Trial design:** RCT  **Setting:** Japan  **TENS groups:** 2  **Comparison groups:** 1 |
| Participants | **Number of participants:** 28 (T1: 11, T2: 9, C1: 8)  **Mean age, years (SD):** T1: 62 (4), T2: 61 (7), C1: 61 (3)  **Age stratification (60+ years):** yes  **Gender (female)**^†^**:** T1: 82%, T2: 89%, C1: 87%  **Chronic LBP type:** mostly spondylosis deformans with/without OA  **Mean chronic LBP duration, years (SD):** T1: 2.8 (1.1), T2; 2.4 (1.4), C1: 2.1 (0.9)  **Leg pain:** mixed with and without leg pain (mixed radicular and non-radicular)  **Population source:** NR |
| Interventions | **Intervention:** TENS (bidirectional modulated sine wave [BMW]) (T1)  **Rationale:** inhibiting effects at dorsal roots, at the dorsal columns, or at peripheral nerves  **Materials:** TENS unit (model HV-F3000 prototype device, Omron Healthcare Co. Ltd.)  **Procedures:**  **Pulse intensity:** 4.5-23 mA (according to tolerance)  **Pulse frequency:** 122 Hz  **Pulse duration:** NR  **Pulse type (burst/continuous):** none  **Electrode placement:** paravertebral  **Format:** in-person  **Duration:** 15 min, 1 session  **Location:** NR  **Provider:** NR  **Mode of delivery:** independent  **Tailoring:** targeted painful area of each patient  **Modifications:** NR  **Adherence:** 100%  **Intervention:** TENS (conventional bidirectional pulsed wave [CPW]) (T2)  **Rationale:** inhibiting effects at dorsal roots, at the dorsal columns, or at peripheral nerves  **Materials:** TENS unit (model HV-F3000 prototype device, Omron Healthcare Co. Ltd.)  **Procedures:**  **Pulse intensity:** 1.5-8.7 mA (according to tolerance)  **Pulse frequency:** 1200 Hz  **Pulse duration:** 200 ms  **Pulse type (burst/continuous):** none  **Electrode placement:** paravertebral  **Format:** in-person  **Duration:** 15 min, 1 session  **Location:** NR  **Provider:** NR  **Mode of delivery:** independent  **Tailoring:** targeted painful area of each patient  **Modifications:** NR  **Adherence:** 100% |
| Comparisons | **Comparisons assessed:**   1. TENS (BMW) (T1) vs. sham TENS (C1) 2. TENS (CPW) (T2) vs. sham TENS (C1)   **Intervention:** sham TENS (C1)  **Materials:** TENS unit (model HV-F3000 prototype device, Omron Healthcare Co. Ltd.)  **Procedures:** no current. All current wave outputs from the TENS devices were not displayed during treatment; all patients were told that the electrotherapeutic device might have effects at subthreshold levels, which they might not be able to feel. Same program and electrode placement as T1 and T2  **Format:** in-person  **Duration:** 15 min, 1 session  **Location:** NR  **Provider:** NR  **Mode of delivery:** independent  **Tailoring:** targeted painful area of each patient  **Modifications:** NR |
| Outcomes | Pain (NRS, 0-10), adverse events (author report)  **Follow-up:** immediate term (closest to 2 weeks) |
| Risk of bias | High (refer to Appendix 2 for details) |
| Notes | Same report as 16000 |
| **Shimoji 2007[13]** (16000) | |
| Methods | **Trial design:** RCT  **Setting:** Japan  **TENS groups:** 1  **Comparison groups:** 1 |
| Participants | **Number of participants:** 21 (T1: 11, C1: 10)  **Mean age, years (SD):** T1: 62 (3), C1: 64 (6)  **Age stratification (60+ years):** yes  **Gender (female)**^†^**:** T1: 82%, C1: 80%  **Chronic LBP type:** mostly spondylosis deformans with/without OA  **Mean chronic LBP duration, years (SD):** T1: 2.5 (0.9), C1: 2.8 (1.1)  **Leg pain:** mixed with and without leg pain (mixed radicular and non-radicular)  **Population source:** NR |
| Interventions | **Intervention:** TENS + massage (T1)  **Rationale:** inhibiting effects at dorsal roots, at the dorsal columns, or at peripheral nerves  **Materials:** TENS unit (model HV-F3000 prototype device, Omron Healthcare Co. Ltd.)  **Procedures:** bidirectional modulated sine wave (BMW)  **Pulse intensity:** 4.5-23 mA (according to tolerance)  **Pulse frequency:** 122 Hz  **Pulse duration:** NR  **Pulse type (burst/continuous):** none  **Electrode placement:** paravertebral  **Format:** in-person  **Duration:** 15 min, 10 sessions, 5 weeks; 15-minute massage to painful area  **Location:** NR  **Provider:** NR  **Mode of delivery:** independent  **Tailoring:** targeted painful area of each patient  **Modifications:** NR  **Adherence:** NR |
| Comparisons | **Comparisons assessed:** TENS + massage (T1) vs. sham TENS + massage (C1)  **Intervention:** sham TENS + massage (C1)  **Materials:** TENS unit (model HV-F3000 prototype device, Omron Healthcare Co. Ltd.)  **Procedures:** no current. All current wave outputs from the TENS devices were not displayed during treatment; all patients were told that the electrotherapeutic device might have effects at subthreshold levels, which they might not be able to feel.  **Format:** in-person  **Duration:** 5 weeks, at least 2 days interval between treatments; 15-minute massage to painful area  **Location:** NR  **Provider:** NR  **Mode of delivery:** independent  **Tailoring:** targeted painful area of each patient  **Modifications:** NR |
| Outcomes | Pain (NRS, 0-10)  **Follow-up:** immediate term (closest to 2 weeks) |
| Risk of bias | High (refer to Appendix 2 for details) |
| Notes | Same report as 11253 |
| **Tella 2021[14]** | |
| Methods | **Trial design:** RCT  **Setting:** Nigeria  **TENS groups:** 1  **Comparison groups:** 1 |
| Participants | **Number of participants:** 34 (T1: 17, C1: 17)  **Mean age, years (SD):** 49.9 (11.73) (not individually reported)  **Age stratification (60+ years):** no  **Gender (female)**^†^**:** 70% (not individually reported)  **Chronic LBP type:** nonspecific (primary)  **Mean chronic LBP duration, years (SD):** NR  **Leg pain:** NR  **Population source:** healthcare |
| Interventions | **Intervention:** TENS + exercise (T1)  **Rationale:** stimulate large-diameter tactile Aβ fibers which close the gate at the substantia gelatinosa of the spinal cord before the arrival of nociceptive Aδ fiber at the hypothetical pain gate. In another assertion, electrical stimulation affects the brain to release endogenous opiates and serotonin that produces analgesia  **Materials:** MH8000P TENS (Taiwan) device  **Procedures:**  **Pulse intensity:** 20-80mA (to tolerance)  **Pulse frequency:** 150 Hz  **Pulse duration:** NR  **Pulse type (burst/continuous):** none  **Electrode placement:** on painful area  **Format:** in-person  **Duration:** 20 min, 10 sessions, 5 weeks  **Location:** physiotherapy outpatient clinic  **Provider:** researcher  **Mode of delivery:** independent  **Tailoring:** targeted to painful area of each patient; intensity varied by patient  **Modifications:** NR  **Adherence:** NR |
| Comparisons | **Comparisons assessed:** TENS + exercise (T1) vs. exercise (C1)  **Intervention:** exercise (C1)  **Materials:** n/a  **Procedures:** active range of motion exercises (ankle, knee, hips); stretching exercises (hip flexors, knee flexors, piriformis); core stabilization exercises (abdominal bracing and drawing in maneuver, each performed 10 repetitions for 2 times per week); isometric strengthening exercises (gluteus medius and maximus, 10 repetitions for 2 times per week); postural re-education in lying, sitting, and standing  **Format:** in-person  **Duration:** 30 min, 10 sessions, 5 weeks  **Location:** physiotherapy outpatient clinic  **Provider:** researcher  **Mode of delivery:** independent  **Tailoring:** postural education when required  **Modifications:** NR |
| Outcomes | Pain (NRS, 0-10), function (RMDQ, 0-24)  **Follow-up:** immediate term (closest to 2 weeks) |
| Risk of bias | High (refer to Appendix 2 for details) |
| **Topuz 2004[15]** | |
| Methods | **Trial design:** RCT  **Setting:** Turkey  **TENS groups:** 2  **Comparison groups:** 1 |
| Participants | **Number of participants:** 45 (T1: 15, T2: 15, C1: 15)  **Mean age, years (SD):** T1: 45.2 (11.2), T2: 50.1 (12), C1: 41.9 (7.7)  **Age stratification (60+ years):** no  **Gender (female)**^†^**:** T1: 60%, T2: 73%, C1: 92%  **Chronic LBP type:** NR (assumed nonspecific primary)  **Mean chronic LBP duration, months (SD):** T1: 16.5 (9.8), T2: 20.5 (14.4), C1: 16.8 (8.7)  **Leg pain:** NR  **Population source:** healthcare |
| Interventions | **Intervention:** conventional TENS (T1)  **Rationale:** invokes both spinal (i.e., gate-control, frequency-dependent blockade) and supraspinal theories (i.e., release of endogenous neuromediators)  **Materials:** TENS unit Trio 300 units (ITO Corp. Japan)  **Procedures:**  **Pulse intensity:** increased to patient's perception of parasthesia  **Pulse frequency:** 80Hz  **Pulse duration:** 100µs  **Pulse type (burst/continuous):** none  **Electrode placement:** bilaterally in a standard dermatomal pattern over the most painful lumbar region  **Format:** in-person  **Duration:** 20 min, 10 sessions, 2 weeks  **Location:** NR  **Provider:** NR  **Mode of delivery:** independent  **Tailoring:** intensity varied by participant  **Modifications:** NR  **Adherence:** NR  **Intervention:** low-frequency TENS (T2)  **Rationale:** stimulate the endogenous opioid system  **Materials:** TENS unit Trio 300 units (ITO Corp. Japan)  **Procedures:**  **Pulse intensity:** increased up to maximum tolerated amplitude without muscle contractions  **Pulse frequency:** 4 Hz  **Pulse duration:** 100 µs  **Pulse type (burst/continuous):** none  **Electrode placement:** bilaterally in a standard dermatomal pattern over the most painful lumbar region  **Format:** in-person  **Duration:** 20 min, 10 sessions, 2 weeks  **Location:** NR  **Provider:** NR  **Mode of delivery:** independent  **Tailoring:** intensity varied by participant  **Modifications:** NR  **Adherence:** NR |
| Comparisons | **Comparisons assessed:**   1. Conventional TENS (T1) vs. sham TENS (C1) 2. Low-frequency TENS (T2) vs. sham TENS (C1)   **Intervention:** sham TENS (C1)  **Materials:** TENS unit Trio 300 units (ITO Corp. Japan)  **Procedures:** same program (without electrical stimulation) and electrode placement as T1 and T2  **Format:** in-person  **Duration:** 20 min, 10 sessions, 2 weeks  **Location:** NR  **Provider:** NR  **Mode of delivery:** independent  **Tailoring:** none  **Modifications:** NR |
| Outcomes | Pain (VAS, 0-10), function (ODI, 0-100), health-related quality of life (SF-36, 0-100)  **Follow-up:** immediate term (closest to 2 weeks) |
| Risk of bias | High (refer to Appendix 2 for details) |
| **Yaksi 2021[16]** | |
| Methods | **Trial design:** RCT  **Setting:** Turkey  **TENS groups:** 2  **Comparison groups:** 1 |
| Participants | **Number of participants:** 74 (T1: 25, T2: 25, C1: 24)  **Mean age, years (SD):** 43.3 (11.3) (not individually reported)  **Age stratification (60+ years):** no  **Gender (female)** ^†^**:** T1: 64%, T2: 52%, C1: 78%  **Chronic LBP type:** mixed degenerative disc disease, disc herniation without radiculopathy  **Mean chronic LBP duration, years (SD):** NR  **Leg pain:** none  **Population source:** healthcare |
| Interventions | **Intervention:** burst TENS (T1)  **Rationale:** the stimulation of large-diameter A-β sensory afferents activates inhibitor interneurons in the substantia gelatinose in the posterior horn of the spinal cord, thus weakening the transmission of nociceptive signals transmitted by small-diameter A-δ and C fibers  **Materials:** TENS Intellect® Advanced (Chattanooga, Mouguerre, France) device  **Procedures:**  **Pulse intensity:** moderate (according to tolerance)  **Pulse frequency:** baseline low frequency (1-4 Hz) combined with high frequency trains (50-100 Hz)  **Pulse duration:** NR  **Pulse type (burst/continuous):** none  **Electrode placement:** lumbar paravertebral  **Format:** in-person  **Duration:** 30 min, 15 sessions, 3 weeks  **Location:** physical medicine and rehabilitation clinic  **Provider:** NR  **Mode of delivery:** independent  **Tailoring:** intensity varied by participant  **Modifications:** NR  **Adherence:** 100%  **Intervention:** conventional TENS (T2)  **Rationale:** the stimulation of large-diameter A-β sensory afferents activates inhibitor interneurons in the substantia gelatinose in the posterior horn of the spinal cord, thus weakening the transmission of nociceptive signals transmitted by small-diameter A-δ and C fibers  **Materials:** TENS Intellect® Advanced (Chattanooga, Mouguerre, France) device  **Procedures:** high-frequency, low-intensity current  **Pulse intensity:** 10-30 mA, moderate (according to tolerance)  **Pulse frequency:** 60-80 Hz  **Pulse duration:** 50-80 μs  **Pulse type (burst/continuous):** none  **Electrode placement:** lumbar paravertebral  **Format:** in-person  **Duration:** 30 min, 15 sessions, 3 weeks  **Location:** physical medicine and rehabilitation clinic  **Provider:** NR  **Mode of delivery:** independent  **Tailoring:** intensity varied by participant  **Modifications:** NR  **Adherence:** 100% |
| Comparisons | **Comparisons assessed:**   1. Burst TENS (T1) vs. sham TENS (C1) 2. Conventional TENS (T2) vs. sham TENS (C1)   **Intervention:** sham TENS (C1)  **Materials:** TENS Intellect® Advanced (Chattanooga, Mouguerre, France) device  **Procedures:** TENS therapy (no current)  **Format:** in-person  **Duration:** 30 min, 15 sessions, 3 weeks  **Location:** physical medicine and rehabilitation clinic  **Provider:** NR  **Mode of delivery:** independent  **Tailoring:** none  **Modifications:** NR |
| Outcomes | Pain (VAS, 0-10), function (modified ODI, 0-50), depression (BDI, 0-63)  **Follow-up:** immediate term (closest to 2 weeks) and short term (closest to 3 months) |
| Risk of bias | Moderate (refer to Appendix 2 for details) |
| Notes | Pain was measured only in the immediate term (i.e., after intervention) |

^†^We used the terms ‘female or male’ to describe gender because these were the terms used by trial authors; however, we recognize that gender is a social construct and sex is a biological construct

**BDI:** Beck Depression Index, **BPI:** Brief Pain Inventory, **HADS:** Hospital Anxiety and Depression Scale, **IFT:** interferential therapy, **LBP:** low back pain, **MPQ:** McGill Pain Questionnaire, **NRS:** numeric rating scale, **NSAIDS:** non-steroidal anti-inflammatory drugs, **ODI:** Oswestry Disability Index, **PCS:** Pain Catastrophizing Scale, **PDI:** Pain Disability Index, **PNF:** proprioceptive neuromuscular facilitation, **RCT:** randomized controlled trial, **RMDQ:** Roland-Morris Disability Questionnaire, **SD:** standard deviation, **SF-36:** 36-item Short Form Health Survey, **TENS:** transcutaneous electrical nerve stimulation, **T1:** TENS treatment group 1, **T2:** TENS treatment group 2, etc. **C1:** comparison treatment group 1, **C2:** comparison treatment group 2, etc. **VAS:** visual analogue scale.

**Online Resource 3.** List of excluded trials with reasons for exclusion (n=61)

| Reason | Trial |
| --- | --- |
| Ineligible publication type  n = 13 | 1. Akhmadeeva L, Rayanova G, Blinova N, Veytsman B. The effects of transcutaneous electrical nerve stimulation (TENS) for patients with low back pain: first two randomized contolled trials in Russia with dynamic TENS devices. Journal of Neurology 2014 (conference paper)[17] 2. Akhmadeeva L, Setchenkova N, Magzhanov R. Effectiveness of transcutaneous dynamic electrical nerve stimulation in low back pain: a pilot randomized controlled trial. Journal of Neurological Sciences 2009; Conference S320[18] 3. Cheing G, Hui-Chan CWY. Repeated applications of transcutaneous electrical nerve stimulation (TENS) produce cumulative effects on chronic clinical pain but not acute experimental pain in chronic low back pain patients. Abstracts of the 8^th^ world congress on the international association for the trial of pain 1996.[19] 4. Efanov OI, [Clinical use of random currents]. 1984[20] 5. Elvir OL, Guevara U, Covarrubias A, White PF. Analgesic efficacy of TENS in chronic low back pain: effect of fixed vs variable-rate stimulation. Anesth Analg 2010; 110:S-366[21] 6. Evanoff A, Newton WP. An alternative treatment for low back pain. J Fam Pract 1999; 48(6):416-7[22] 7. Hazime FA, de Freitas DG, Monteiro RL, Maretto RL, de Almeida Carvalho NA, Hasue RH, Amado João SM. Analgesic efficacy of cerebral and peripheral electrical stimulation in chronic nonspecific low back pain: a randomized, double-blind, factorial clinical trial. BMC Musculoskeletal Disorders 2015;16:7[23] 8. Hush J. TENS of unknown value in the treatment of chronic low back pain. Commentary. 2006[24] 9. Jamison R, Wan L, Ross E, Edwards R. Efficacy of high-frequency transcutaneous electrical nerve stimulation for chronic low back pain: does hypersensitivity matter? 2018[25] 10. Ketenci A, Yahsi E. Evaluation of the pain, neuropathic pain and sympathetic skin response to transcutaneous electrical nerve stimulation treatment in chronic mechanical low back pain patients. Osteoporosis Int 2017; 28(Suppl 1):S127-S636[26] 11. Magbagbeola J, Khawaki YN, Al-Nandi M, Farrag H. Transcutaneous electrical nerve stimulation (TENS) for relieving low-back pain in Saudi Arabia, Eastern Province. 1987 (Poster 707)[27] 12. Sodipo JOA. Transcutaneous electrical nerve stimulation (TENS) and acupuncture: comparison of therapy for low-back pain. Pain 1981; Supp1:S277[28] 13. Thiese MS, Hughes M, Biggs J. Electrical stimulation for chronic non-specific low back pain in a working-age population: a 12-week double blinded randomized controlled trial. BMC Musculoskelet Disord 2013; 14:117[29] |
| Ineligible trial design  n = 6 | 1. Itoh K, Itoh S, Katsumi Y, Kitakoji H. A pilot trial on using acupuncture and transcutaneous electrical nerve stimulation to treat chronic non-specific low back pain. Complement Ther Clin Pract 2009;15(1):22-25[30] 2. Kaur G. An Experimental Trial to See the Efficacy of Manual Therapy and Conventional Therapy in Low Back Pain. Indian Journal of Physiotherapy and Occupational Therapy 2015; 9(4):23-27[31] 3. Marchand S, Charest J, Li J, Chenard J-R, Lavignolle B, Laurencelle L. Is TENS purely a placebo effect? A controlled trial on chronic low back pain. Pain 1993; 54(1):99-106[32] 4. Poitras S, Brosseau L. Evidence-informed management of chronic low back pain with transcutaneous electrical nerve stimulation, interferential current, electrical muscle stimulation, ultrasound, and thermotherapy. Spine J 2008; 8(1):226-33[33] 5. Popovic DB, Bijelic G, Miler V, Dosen S, Popovic MB, Schwirtlich L. Lumbar stimulation belt for therapy of low-back pain. Artif Organs 2009; 33(1):54-60[34] 6. Rutkowski B, Niedzialkowska T, Otto J. Electrical stimulation in chronic low-back pain. Br J Anaesth 1977; 49(6):629-32[35] |
| Ineligible population  n = 19 | 1. Akhmadeeva LR, Setchenkova NM, Magzhanov RV, Abdrashitova EV, Bulgakova AZ. (Randomized blind placebo-controlled trial of the effectiveness of transcutaneous adaptive electrostimulation in the treatment of nonspecific low back pain) [Russian]. Zh Nevrol Psikhiatr Im S S Korsakova 2010; 110(4):57-62[36] 2. Aguilar Ferrandiz ME, Nijs J, Gidron Y, Roussel N, Vanderstraeten R, Van Dyck D, Huysmans E, De Kooning M. Auto-Targeted Neurostimulation Is Not Superior to Placebo in Chronic Low Back Pain: A Fourfold Blind Randomized Clinical Trial. Pain Physician 2016; 19:E707-E719[37] 3. Bergeron-Vézina, Kayla, Filion, Camille, Couture, Chantal, Vallée, Élisabeth, Laroche, Sarah, Léonard, Guillaume. Adjusting Pulse Amplitude During Transcutaneous Electrical Nerve Stimulation Does Not Provide Greater Hypoalgesia. J Altern Complement Med 2018; 24(3):262-267[38] 4. Buchmuller A, Navez M, Milletre-Bernardin M, Pouplin S, Presles E, Lanteri-Minet M, Tardy B, Laurent B, Camdessanche JP, on behalf of the Lombotens Trial Group. Value of TENS for relief of chronic low back pain with or without radicular pain. Eur J Pain 2012; 16(5):656-65[39] 5. Deyo RA, Walsh NE, Martin DC, Schoenfeld LS, Ramamurthy S. A controlled trial of transcutaneous electrical nerve stimulation (TENS) and exercise for chronic low back pain. N Engl J Med 1990; 322(23):1627-34[40] 6. Hawamdeh M, Al Malty AAM, Tariah HA, Omar MH, Shtayyat WR. Comparison between the Effectiveness of using Transcutaneous Electrical Nerve Stimulation (TENS) with Back Strengthening Exercises and the use of Back Strengthening Exercises only on Patients with Chronic Low Back Pain: a Pilot Crossover Double Blind Trial. Indian Journal of Physiotherapy and Occupational Therapy 2015;9(2):210-214[41] 7. Facci LM, Nowotny JP, Tormem F, Trevisani VFM. Effects of transcutaneous electrical nerve stimulation (TENS) and interferential currents (IFC) in patients with nonspecific chronic low back pain: randomized clinical trial. Sao Paulo Med J 2011; 129(4):206-16[42] 8. Hsieh RL, Lee WC. One-shot percutaneous electrical nerve stimulation vs. transcutaneous electrical nerve stimulation for low back pain: comparison of therapeutic effects. Am J Phys Med Rehabil 2002; 81(11):838-43[43] 9. Hsieh CY, Phillips RB, Adams AH, Pope MH. Functional outcomes of low back pain: comparison of four treatment groups in a randomized controlled trial. J Manipulative Physiol Ther 1992: 15(1):4-9[44] 10. Jarzem PF, Harvey EJ, Arcaro N, Kaczorowski J. Transcutaneous electrical nerve stimulation [TENS] for chronic low back pain. Journal of Musculoskeletal Pain 2005; 13(2):3-9[45] 11. Jarzem PF, Harvey EJ, Arcaro N, Kaczorowski J. Transcutaneous electrical nerve stimulation for short-term treatment of low back pain - Randomized double blind crossover trial of sham versus conventional TENS. Journal of Musculoskeletal Pain 2005; 13(2):11-17[46] 12. Kumar S, Sharma VP, Tripathi HK, Negi MPS, Vendhan GV. Efficacy of dynamic muscular stabilization techniques (DMST) over conventional techniques in patients with chronic low back pain. Indian Journal of Physiotherapy and Occupational Therapy 2009;3(2):47-53[47] 13. Leemans L, Elma Ö, Nijs J, Wideman TH, Siffain C, den Bandt H, Van Laere S, Beckwée D. Transcutaneous electrical nerve stimulation and heat to reduce pain in a chronic low back pain population: a randomized controlled clinical trial. Braz J Phys Ther 2021; 25(1):86-96[48] 14. Lehmann TR, Russell DW, Spratt KF. The impact of patients with nonorganic physical findings on a controlled trial of transcutaneous electrical nerve stimulation and electroacupuncture. Spine 1983; 8(6):625-34[49] 15. Lehmann TR, Russell DW, Spratt KF, Colby H, Liu KY, Fairchild ML, Christensen S. Efficacy of electroacupuncture and TENS in the rehabilitation of chronic low back pain patients. Pain 1986; 26(3):277-290[50] 16. Moore SR, Shurman J. Combined neuromuscular electrical stimulation and transcutaneous electrical nerve stimulation for treatment of chronic back pain: a double-blind, repeated measures comparison. Arch Phys Med Rehabil 1997; 78(1):55-60[51] 17. Sakai T, Tsutani K, Tsukayama H, Nakamura T, Ikeuchi T, Kawamoto M, Kasuya D. Multi-center randomized controlled trial of acupuncuture with electric stimulation and acupuncture-like transcutaneous electrical nerve stimulation for lumbago (in Japanese). Journal of the Japan Society of Acupuncture Moxibustion 2001; 175-184[52] 18. Wang L, Fan W, Yu C, Lang M, Sun G. Clinical effects of electrical stimulation therapy on lumbar disc herniation-induced sciatica and its influence on peripheral ROS level. Journal of Musculoskeletal and Neuronal Interactions 2018; 18(3):393-398[53] 19. Warke K, Al-Smadi J, Baxter D, Walsh DM, Lowe-Strong AS. Efficacy of transcutaneous electrical nerve stimulation (TENS) for chronic low-back pain in a multiple sclerosis population: a randomized, placebo-controlled clinical trial. Clin J Pain 2006; 22(9):812-9[54] |
| Ineligible intervention  n = 13 | 1. Alrwaily M, Schneider M, Sowa G, Timko M, Whitney SL, Delitto A. Stabilization exercises combined with neuromuscular electrical stimulation for patients with chronic low back pain: a randomized controlled trial. Brazilian Journal of Physical Therapy 2019;23(6):506-515[55] 2. Bordiak FC, da Silva EB. Electrical stimulation and core training on pain and range of motion in low back pain. Fisioter Mov 2012; 25(4):759-66[56] 3. Durmus D, Akyol Y, Alayli G, Tander B, Zahiroglu Y, Canturk F. Effects of electrical stimulation program on trunk muscle strength, functional capacity, quality of life, and depression in the patients with low back pain: a randomized controlled trial. Rheumatol Int 2009; 29(8):947-54[57] 4. Durmus D, Durmaz Y, Canturk F. Effects of therapeutic ultrasound and electrical stimulation program on pain, trunk muscle strength, disability, walking performance, quality of life, and depression in patients with low back pain: a randomized-controlled trial. Rheumatol Int 2010; 30(7):901-10[58] 5. Kim YK, Cho SY, Lee KH. Effects of transcutaneous electrical nerve stimulation and instrument-assisted soft tissue mobilization combined treatment on chronic low back pain: a randomized controlled trial. J Back Musculoskelet Rehabil 2021; 34(5):895-902[59] 6. Kim TH, Kim EH, Cho HY. The effects of the CORE programme on pain at rest, movement-induced and secondary pain, active range of motion, and proprioception in female office workers with chronic low back pain: a randomized controlled trial.Clin Rehabil 2015;29(7):653-62[60] 7. Neuwersch-Sommeregger S, Kostenberger M, Pipam W, Breschan C, Stettner H, Demschar S, Trummer B, Likar R. [Electrical muscle stimulation in combination with heat for patients with chronic, nonspecific low back pain : A randomized, double-blind, stratified, placebo-controlled clinical trial]. The Pain 2020;34:65-73[61] 8. Ozkaraoglu DK, Tarakci D, Algun ZC. Comparison of two different electrotherapy methods in low back pain treatment. J Back Musculoskelet Rehabil 2020; 33(2):193-199[62] 9. Pelegrini ACA, Gasoto E, Bussolaro JM, Segatti G, de Albuquerque CE, Bertolini GRF. The analgesic action of Aussie current in women with non-specific chronic lumbar pain. International Journal of Therapy and Rehabilitation 2019[63] 10. Potturi G, Sundaresan AN, Mahendran J, Karthikeyan PD, Reddy VK. Effect of Dexamethasone Iontophoresis Combined with Strong Surged Faradic Current on Piriformis Syndrome - A Simple Randomized Control Clinical Trial. Indian Journal of Physiotherapy and Occupational Therapy 2014; 8(4):265-271[64] 11. Schabrun SM, Jones E, Elgueta Cancino EL, Hodges PW. Targeting chronic recurrent low back pain from the top-down and the bottom-up: a combined transcranial direct current stimulation and peripheral electrical stimulation intervention. Brain Stimul 2014; 7(3):451-9[65] 12. Starkweather AR, Coyne P, Lyon DE, Elswick RK, An K, Sturgill J. Decreased low back pain intensity and differential gene expression following Calmare®: results from a double-blinded randomized sham-controlled trial. Res Nurs Health 2015; 38(1):29-38[66] 13. Thompson JW, Bower S, Tyrer SP. A double blind randomised controlled clinical trial on the effect of transcutaneous spinal electroanalgesia (TSE) on low back pain. Eur J Pain 2008; 12(3):371-7[67] |
| Ineligible comparison  n = 8 | 1. Ebadi S, Ansari NN, Ahadi T, Fallah E, Forogh B. No immediate analgesic effect of diadynamic current in patients with nonspecific low back pain in comparison to TENS. J Bodyw Mov Ther 2018; 22(3):693-99[68] 2. Ghoname EA, Craig WF, White PF, Ahmed HE, Hamza MA, Henderson BN, Gajraj NM, Huber PJ, Gatchel RJ. Percutaneous electrical nerve stimulation for low back pain: a randomized crossover trial. JAMA 1999; 281(9):818-23[69] 3. Ghoname EA, White PF, Ahmed HE, Hamza MA, Craig WF, Noe CE. Percutaneous electrical nerve stimulation: an alternative to TENS in the management of sciatica. Pain 1999; 83(2):193-9[70] 4. Grabianska E, Lesniewicz J, Pieszynski I, Kostka J. Porownanie dzialania przeciwbolowego pradow interferencyjnych i TENS u pacjentow z dolegliwosciami bolowymi w czesci ledzwiowo-krzyzowej kregoslupa (Comparison of the analgesic effect of interferential current (IFC) and TENS in patients with low back pain) [Polish]. Wiad Lek 2015; 68(1):13-9[71] 5. Rajfur J, Pasternok M, Rajfur K, Walewicz K, Fras B, Bolach B, Dymarek R, Rosinczuk J, Halski T, Taradaj J. Efficacy of Selected Electrical Therapies on Chronic Low Back Pain: a Comparative Clinical Pilot Trial. Med Sci Monit 2017; 23:85-100[72] 6. Ratajczak B, Hawrylak A, Demida A, Kuciel-Lewandowska J, Boerner E. Effectiveness of diadynamic currents and transcutaneous electrical nerve stimulation in disc disease lumbar part of spine. J Back Musculoskelet Rehabil 2011; 24(3):155-9.[73] 7. Tousignant-Laflamme Y, Laroche C, Beaulieu C, Bouchard A-J, Boucher S, Michaud-Létourneau M. A randomized trial to determine the duration of analgesia following a 15- and a 30-minute application of acupuncture-like TENS on patients with chronic low back pain. Physiotherapy Theory and Practice 2017; 33(5):361-369[74] 8. Weizemann C, Flores Camargo N, Vieira Barboza T, Rodrigo de Carvalho A, Flor Bertolini GR. Effect of transcutaneous electrical nerve stimulation and hypnosis on chronic low back pain. BrJP 2021; 4(1):26-30[75] |
| Ineligible outcomes  n = 2 | 1. Ariel E. Levkovitz Y. Goor-Aryeh I. Motti R. The effects of TENS, interferential stimulation, and combined interferential stimulation and pulsed ultrasound on patients with disc herniation-induced radicular pain. J Back Musculoskelet Rehabil 2022; 35(2):363-371[76] 2. Rojhani-Shirazi Z, Rezaeian T. The effects of Transcutaneous Electrical Nerve Stimulation on postural control in patients with chronic low back pain. Journal of Medicine and Life 2015; 8(Spec Iss 2):19-27[77] |

**Online Resource 4.** Risk of bias assessment for the included RCTs (n=17) (16 reports)

| Trial | Random allocation | Concealed allocation | Participant blinding | Provider blinding | Assessor blinding | Drop-outs | Intention-to-treat analysis | Selective reporting | Similar groups (baseline) | Co-interventions | Compliance | Timing of outcomes | Other biases | Overall ROB Rating |
| --- | --- | --- | --- | --- | --- | --- | --- | --- | --- | --- | --- | --- | --- | --- |
|  | Selection bias | | Performance bias | | Detection bias | Attrition bias | | Reporting bias | Selection bias | Other biases | | | |  |
| Alizadeh 2009[1] | 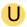 | 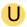 | 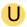 | 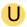 | 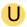 | 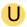 | 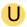 | 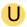 | 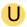 | 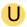 | 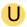 | 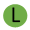 | 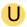 | High |
| Bloodworth 2004[2] | 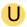 | 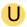 | 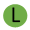 | 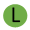 | 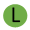 | 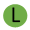 | 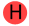 | 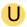 | 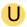 | 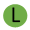 | 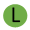 | 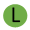 | 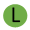 | High |
| Cheing 1999[3] | 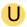 | 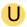 | 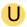 | 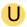 | 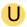 | 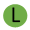 | 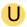 | 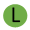 | 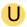 | 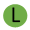 | 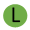 | 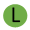 | 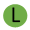 | High |
| De Alencar Caldas 2021[4] | 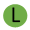 | 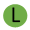 | 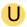 | 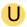 | 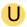 | 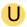 | 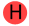 | 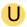 | 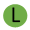 | 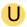 | 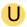 | 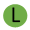 | 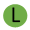 | High |
| Depaoli Lemos 2021[5] | 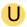 | 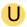 | 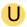 | 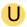 | 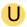 | 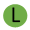 | 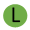 | 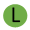 | 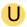 | 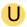 | 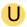 | 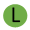 | 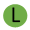 | High |
| Dias 2021[6] | 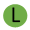 | 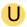 | 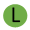 | 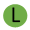 | 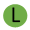 | 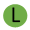 | 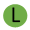 | 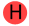 | 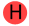 | 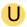 | 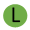 | 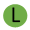 | 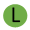 | High |
| Elserty 2016[7] | 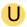 | 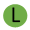 | 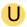 | 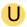 | 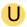 | 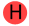 | 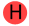 | 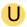 | 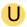 | 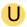 | 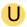 | 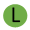 | 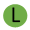 | High |
| Ezema 2022[8] | 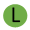 | 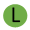 | 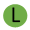 | 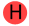 | 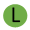 | 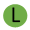 | 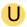 | 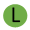 | 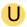 |  |  |  |  | Unclear |
| Jamison 2019[9] |  |  |  |  |  |  |  |  |  |  |  |  |  | Unclear |
| Kibar 2020[10] |  |  |  |  |  |  |  |  |  |  |  |  |  | High |
| Kofotolis 2008[11] |  |  |  |  |  |  |  |  |  |  |  |  |  | High |
| Petrofsky 2020[12] |  |  |  |  |  |  |  |  |  |  |  |  |  | High |
| Shimoji 2007[13] (11253) |  |  |  |  |  |  |  |  |  |  |  |  |  | High |
| Shimoji 2007[13] (16000) |  |  |  |  |  |  |  |  |  |  |  |  |  | High |
| Tella 2021[14] |  |  |  |  |  |  |  |  |  |  |  |  |  | High |
| Topuz 2004[15] |  |  |  |  |  |  |  |  |  |  |  |  |  | High |
| Yaksi 2021[16] |  |  |  |  |  |  |  |  |  |  |  |  |  | Unclear |

Risk of bias assessment conducted using ROB1 criteria. H=high risk of bias, L=low risk of bias, ROB=risk of bias, U=Unclear risk of bias

Risk of bias graph: review authors’ judgements about each risk of bias item presented as percentages across all included RCTs

**Online Resource 5.** GRADE Evidence Profile tables

GRADE Evidence Profile table 1: ***What are the benefits and harms of TENS in the management of community-dwelling adults (including older adults aged ≥60 years) with chronic primary low back pain (with or without leg pain) compared with sham?***

| **Certainty assessment** | | | | | | | **№ of patients** | | **Effect** | | **Certainty** | **Importance** |
| --- | --- | --- | --- | --- | --- | --- | --- | --- | --- | --- | --- | --- |
| **№ of trials** | **Trial design** | **Risk of bias** | **Inconsistency** | **Indirectness** | **Imprecision** | **Other considerations** | **TENS** | **Sham** | **Relative (95% CI)** | **Absolute (95% CI)** |  |  |
| **ALL ADULTS** | | | | | | | | | | | | |
| **Pain (follow-up: closest to 2 weeks; assessed with: VAS, NRS, Borg scale; benefit indicated by lower values; Scale from: 0 to 10)** | | | | | | | | | | | | |
| 9^a^ | randomised trials | very serious^1,2,3,4,5,6,7,8,b^ | serious^c^ | not serious^d^ | serious^e^ | none | 280 | 187 | - | MD **0.9 lower** (1.54 lower to 0.26 lower) | ⨁◯◯◯ Very low | CRITICAL |
|  | | | | | | | | | | | | |
| **Pain in females (follow-up: closest to 2 weeks; assessed with: Borg scale; benefit indicated by lower values; Scale from: 0 to 10)** | | | | | | | | | | | | |
| 1 | randomised trials | very serious^5,b^ | not serious^g^ | serious^h^ | serious^j^ | none | 23 | 21 | - | MD **0.1 higher** (0.2 lower to 0.4 higher) | ⨁◯◯◯ Very low | CRITICAL |
| **Pain in females and males (follow-up: closest to 2 weeks; assessed with: VAS, NRS, Borg scale; benefit indicated by lower values; Scale from: 0 to 10)** | | | | | | | | | | | | |
| 8 | randomised trials | very serious^b^ | serious^k^ | not serious^d^ | serious^l^ | none | 257 | 187 | - | MD **1.03 lower** (1.69 lower to 0.36 lower) | ⨁◯◯◯ Very low | CRITICAL |
| **Pain in people without leg pain (follow-up: closest to 2 weeks weeks; assessed with: VAS, NRS, Borg scale; benefit indicated by lower values; Scale from: 0 to 10)** | | | | | | | | | | | | |
| 5 | randomised trials | very serious^1,2,4,5,8,b^ | serious^m^ | not serious^d^ | serious^n^ | none | 129 | 102 | - | MD **0.64 lower** (1.83 lower to 0.54 higher) | ⨁◯◯◯ Very low | CRITICAL |
| **Pain in people with unclassified presence of leg pain (follow-up: closest to 2 weeks; assessed with: VAS, NRS; benefit indicated by lower values; Scale from: 0 to 10)** | | | | | | | | | | | | |
| 2^o^ | randomised trials | very serious^3,7,b^ | not serious^p^ | not serious^q^ | serious^l^ | none | 100 | 47 | - | MD **1.34 lower** (2.44 lower to 0.25 lower) | ⨁◯◯◯ Very low | CRITICAL |
| **Pain in people with mixed radicular and non-radicular leg pain (follow-up: closest to 2 weeks; assessed with: NRS; benefit indicated by lower values; Scale from: 0 to 10)** | | | | | | | | | | | | |
| 2^r^ | randomised trials | very serious^6,10,b^ | very serious^s^ | not serious^q^ | very serious^t^ | none | 51 | 38 | - | MD **0.96 lower** (4.59 lower to 2.67 higher) | ⨁◯◯◯ Very low | CRITICAL |
|  | | | | | | | | | | | | |
| **Pain in trials undertaken in high to upper-middle income countries (follow-up: closest to 2 weeks; assessed with: VAS, NRS, Borg scale; benefit indicated by lower values; Scale from: 0 to 10)** | | | | | | | | | | | | |
| 8^u^ | randomised trials | very serious^1,2,3,5,6,7,8,10,b^ | serious^v^ | not serious^d^ | serious^l^ | none | 219 | 125 | - | MD **1.01 lower** (1.69 lower to 0.34 lower) | ⨁◯◯◯ Very low | CRITICAL |
| **Pain in trials undertaken in low to lower-middle income countries (follow-up: closest to 2 weeks; assessed with: NRS; benefit indicated by lower values; Scale from: 0 to 10)** | | | | | | | | | | | | |
| 1 | randomised trials | serious^4,w^ | not serious^g^ | serious^x^ | serious^j^ | none | 30 | 32 | - | MD **0**  (0.4 lower to 0.4 higher) | ⨁◯◯◯ Very low | CRITICAL |
| **Pain in trials using a single TENS treatment session (follow-up: closest to 2 weeks; assessed with: VAS, NRS; benefit indicated by lower values; Scale from: 0 to 10)** | | | | | | | | | | | | |
| 4^y^ | randomised trials | very serious^1,3,4,6,b^ | very serious^z^ | not serious^d^ | serious^n^ | none | 135 | 90 | - | MD **0.68 lower** (2 lower to 0.65 higher) | ⨁◯◯◯ Very low | CRITICAL |
| **Pain in trials using 10-20 TENS treatment sessions (follow-up: closest to 2 weeks; assessed with: VAS, Borg scale; benefit indicated by lower values; Scale from: 0 to 10)** | | | | | | | | | | | | |
| 5^aa^ | randomised trials | very serious^2,5,7,8,10,b^ | serious^ab^ | not serious^q^ | serious^l^ | none | 145 | 97 | - | MD **1.06 lower** (1.94 lower to 0.18 lower) | ⨁◯◯◯ Very low | CRITICAL |
|  | | | | | | | | | | | | |
| **Pain (after removing high risk of bias trials) (follow-up: closest to 2 weeks; assessed with: VAS, NRS; benefit indicated by lower values; Scale from: 0 to 10)** | | | | | | | | | | | | |
| 2 | randomised trials | serious^4,8,ac^ | serious^ad^ | not serious^d^ | very serious^t^ | none | 80 | 55 | - | MD **0.63 lower** (2.78 lower to 1.53 higher) | ⨁◯◯◯ Very low | CRITICAL |
| **Pain (follow-up: closest to 3 months; assessed with: VAS, Borg scale; benefit indicated by lower values; Scale from: 0 to 10)** | | | | | | | | | | | | |
| 2^ae^ | randomised trials | very serious^5,8,af^ | serious^ag^ | not serious^q^ | very serious^t^ | none | 73 | 44 | - | MD **0.4 lower** (2.21 lower to 1.41 higher) | ⨁◯◯◯ Very low | CRITICAL |
| **Pain in females (follow-up: closest to 3 months; assessed with: Borg scale; benefit indicated by lower values; Scale from: 0 to 10)** | | | | | | | | | | | | |
| 1 | randomised trials | very serious^5,af^ | not serious^g^ | serious^h^ | serious^j^ | none | 23 | 21 | - | MD **0.1 higher** (0.23 lower to 0.43 higher) | ⨁◯◯◯ Very low | CRITICAL |
| **Pain in females and males (follow-up: closest to 3 months; assessed with: VAS; benefit indicated by lower values; Scale from: 0 to 10)** | | | | | | | | | | | | |
| 1^ae^ | randomised trials | serious^8,w^ | not serious^g^ | serious^h^ | very serious^ah^ | none | 50 | 23 | - | MD **1.06 lower** (4.23 lower to 2.12 higher) | ⨁◯◯◯ Very low | CRITICAL |
|  | | | | | | | | | | | | |
| **Pain (after removing high risk of bias trials) (follow-up: closest to 3 months; assessed with: VAS; benefit indicated by lower values; Scale from: 0 to 10)** | | | | | | | | | | | | |
| 1^ae^ | randomised trials | serious^8,w^ | not serious^g^ | serious^h^ | very serious^ah^ | none | 50 | 23 | - | MD **1.06 lower** (4.23 lower to 2.12 higher) | ⨁◯◯◯ Very low | CRITICAL |
| **Pain stratified by race/ethnicity** | | | | | | | | | | | | |
| 0 |  |  |  |  |  |  |  |  |  |  |  | CRITICAL |
| **Function (follow-up: closest to 2 weeks; assessed with: ODI, RMDQ; benefit indicated by lower values)** | | | | | | | | | | | | |
| 4^ai^ | randomised trials | very serious^2,5,7,10,b^ | very serious^aj^ | not serious^q^ | very serious^ak^ | none | 95 | 74 | - | SMD **0.96 SD lower** (3.2 lower to 1.28 higher) | ⨁◯◯◯ Very low | CRITICAL |
| **Function in females and males (follow-up: closest to 2 weeks; assessed with: ODI, RMDQ; benefit indicated by lower values)** | | | | | | | | | | | | |
| 3 | randomised trials | very serious^2,7,10,b^ | very serious^aj^ | not serious^q^ | very serious^ak^ | none | 72 | 53 | - | SMD **1.3 lower** (4.38 lower to 1.78 higher) | ⨁◯◯◯ Very low | CRITICAL |
|  | | | | | | | | | | | | |
|  | | | | | | | | | | | | |
|  | | | | | | | | | | | | |
| **Function in females (follow-up: closest to 2 weeks; assessed with: ODI; benefit indicated by lower values)** | | | | | | | | | | | | |
| 1 | randomised trials | very serious^5,af^ | not serious^g^ | serious^h^ | very serious^ah^ | none | 23 | 21 | - | SMD **0.27 higher** (0.33 lower to 0.86 higher) | ⨁◯◯◯ Very low | CRITICAL |
| **Function in people with no leg pain (follow-up: closest to 2 weeks; assessed with: ODI, RMDQ; benefit indicated by lower values)** | | | | | | | | | | | | |
| 2 | randomised trials | very serious^2,5,b^ | not serious^p^ | not serious^q^ | very serious^al^ | none | 34 | 32 | - | SMD **0.16 higher** (1.19 lower to 1.51 higher) | ⨁◯◯◯ Very low | CRITICAL |
| **Function in people either with or without radicular leg pain (follow-up: closest to 2 weeks; assessed with: RMDQ; benefit indicated by lower values)** | | | | | | | | | | | | |
| 1 | randomised trials | very serious^10,b^ | not serious^g^ | serious^h^ | serious^am^ | none | 31 | 30 | - | SMD **1.97 lower** (2.59 lower to 1.36 lower) | ⨁◯◯◯ Very low | CRITICAL |
| **Function in people with unclassified presence of leg pain (follow-up: closest to 2 weeks; assessed with: ODI; benefit indicated by lower values)** | | | | | | | | | | | | |
| 1^ai^ | randomised trials | very serious^7,b^ | not serious^g^ | serious^h^ | very serious^al^ | none | 30 | 12 | - | SMD **1.67 higher** (28.66 lower to 25.33 higher) | ⨁◯◯◯ Very low | CRITICAL |
|  | | | | | | | | | | | | |
| **Function (follow-up: closest to 3 months; assessed with: ODI; benefit indicated by lower values; Scale from: 0 to 50)** | | | | | | | | | | | | |
| 2^ae^ | randomised trials | very serious^5,8,af^ | serious^an^ | not serious^q^ | serious^ao^ | none | 73 | 44 | - | MD **0.24 lower** (4.3 lower to 3.81 higher) | ⨁◯◯◯ Very low | CRITICAL |
| **Function in females (follow-up: closest to 3 months; assessed with: ODI; benefit indicated by lower values; Scale from: 0 to 50)** | | | | | | | | | | | | |
| 1 | randomised trials | very serious^5,b^ | not serious^g^ | serious^h^ | serious^ao^ | none | 23 | 21 | - | MD **0.5 higher** (1.22 lower to 2.22 higher) | ⨁◯◯◯ Very low | CRITICAL |
| **Function in females and males (follow-up: closest to 3 months; assessed with: ODI; benefit indicated by lower values; Scale from: 0 to 50)** | | | | | | | | | | | | |
| 1^ae^ | randomised trials | serious^8,w^ | not serious^g^ | serious^h^ | serious^ap^ | none | 50 | 23 | - | MD **2.61 lower** (6.42 lower to 1.2 higher) | ⨁◯◯◯ Very low | CRITICAL |
| **Function (after removing high risk of bias trials) (follow-up: closest to 3 months; assessed with: ODI; Scale from: 0 to 50)** | | | | | | | | | | | | |
| 1^ae^ | randomised trials | serious^8,w^ | not serious^g^ | serious^h^ | serious^ao^ | none | 50 | 23 | - | MD **2.61 lower** (6.42 lower to 1.2 higher) | ⨁◯◯◯ Very low | CRITICAL |
|  | | | | | | | | | | | | |
| **Function stratified by race/ethnicity** | | | | | | | | | | | | |
| 0 |  |  |  |  |  |  |  |  |  |  |  | CRITICAL |
| **Function in trials undertaken in low to lower-middle income countries** | | | | | | | | | | | | |
| 0 |  |  |  |  |  |  |  |  |  |  |  | CRITICAL |
| **Function by number of treatment sessions** | | | | | | | | | | | | |
| 0 |  |  |  |  |  |  |  |  |  |  |  | CRITICAL |
| **Health-related quality of life (follow-up: closest to 2 weeks; assessed with: SF-36 (PCS); benefit indicated by higher values; Scale from: 0 to 100)** | | | | | | | | | | | | |
| 2^ai^ | randomised trials | very serious^2,7,b^ | serious^an^ | not serious^q^ | very serious^aq^ | none | 41 | 23 | - | MD **3.21 higher** (21.17 lower to 27.59 higher) | ⨁◯◯◯ Very low | CRITICAL |
| **Health-related quality of life in people with no radicular leg pain (follow-up: closest to 2 weeks; assessed with: SF-36 (PCS); benefit indicated by higher values; Scale from: 0 to 100)** | | | | | | | | | | | | |
| 1 | randomised trials | very serious^2,b^ | not serious^g^ | serious^h^ | very serious^ar^ | none | 11 | 11 | - | MD **20.45 lower** (56.67 lower to 15.77 higher) | ⨁◯◯◯ Very low | CRITICAL |
|  | | | | | | | | | | | | |
|  | | | | | | | | | | | | |
| **Health-related quality of life in people with unclassified presence of leg pain (follow-up: closest to 2 weeks; assessed with: SF-36 (PCS); benefit indicated by higher values; Scale from: 0 to 100)** | | | | | | | | | | | | |
| 1^ai^ | randomised trials | very serious^7,b^ | not serious^g^ | serious^h^ | serious^as^ | none | 30 | 12 | - | MD **5.91 higher** (0.44 lower to 12.26 higher) | ⨁◯◯◯ Very low | CRITICAL |
| **Health-related quality of life (follow-up: closest to 2 weeks; assessed with: SF-36 (MCS); benefit indicated by higher values; Scale from: 0 to 100)** | | | | | | | | | | | | |
| 2^ai^ | randomised trials | very serious^2,7,b^ | very serious^at^ | serious^h^ | serious^as^ | none | 41 | 23 | - | MD **3.57 higher** (30.06 lower to 37.2 higher) | ⨁◯◯◯ Very low | CRITICAL |
| **Health-related quality of life in people with no radicular leg pain (follow-up: closest to 2 weeks; assessed with: SF-36 (MCS); benefit indicated by higher values; Scale from: 0 to 100)** | | | | | | | | | | | | |
| 1 | randomised trials | very serious^2,b^ | not serious^g^ | serious^h^ | serious^au^ | none | 11 | 11 | - | MD **11.63 lower** (20.59 lower to 2.67 lower) | ⨁◯◯◯ Very low | CRITICAL |
| **Health-related quality of life in people with unclassified presence of leg pain (follow-up: closest to 2 weeks; assessed with: SF-36 (MCS); benefit indicated by higher values; Scale from: 0 to 100)** | | | | | | | | | | | | |
| 1^ai^ | randomised trials | very serious^7,b^ | not serious^g^ | serious^h^ | serious^l^ | none | 30 | 12 | - | MD **11.63 higher** (9.96 higher to 13.31 higher) | ⨁◯◯◯ Very low | CRITICAL |
| **Health-related quality of life stratified by gender** | | | | | | | | | | | | |
| 0 |  |  |  |  |  |  |  |  |  |  |  | CRITICAL |
| **Health-related quality of life stratified by race/ethnicity** | | | | | | | | | | | | |
| 0 |  |  |  |  |  |  |  |  |  |  |  | CRITICAL |
| **Health-related quality of life in trials undertaken in low to lower-middle income countries** | | | | | | | | | | | | |
| 0 |  |  |  |  |  |  |  |  |  |  |  | CRITICAL |
| **Health-related quality of life stratified by number of treatment sessions** | | | | | | | | | | | | |
| 0 |  |  |  |  |  |  |  |  |  |  |  | CRITICAL |
| **Depression (follow-up: closest to 3 months; assessed with: BDI; benefit indicated by lower values; Scale from: 0 to 63)** | | | | | | | | | | | | |
| 1^ae^ | randomised trials | serious^8,w^ | not serious^g^ | serious^h^ | very serious^av^ | none | 50 | 23 | - | MD **3.04 higher** (19.15 lower to 25.22 higher) | ⨁◯◯◯ Very low | CRITICAL |
| **Depression stratified by gender** | | | | | | | | | | | | |
| 0 |  |  |  |  |  |  |  |  |  |  |  | CRITICAL |
| **Depression stratified by presence of leg pain** | | | | | | | | | | | | |
| 0 |  |  |  |  |  |  |  |  |  |  |  | CRITICAL |
| **Depression stratified by national economic development** | | | | | | | | | | | | |
| 0 |  |  |  |  |  |  |  |  |  |  |  | CRITICAL |
| **Depression stratified by race/ethnicity** | | | | | | | | | | | | |
| 0 |  |  |  |  |  |  |  |  |  |  |  | CRITICAL |
| **Depression stratified by number of treatment sessions** | | | | | | | | | | | | |
| 0 |  |  |  |  |  |  |  |  |  |  |  | CRITICAL |
| **Fear avoidance** | | | | | | | | | | | | |
| 0 |  |  |  |  |  |  |  |  |  |  |  | CRITICAL |
| **Catastrophizing** | | | | | | | | | | | | |
| 0 |  |  |  |  |  |  |  |  |  |  |  | CRITICAL |
| **Anxiety** | | | | | | | | | | | | |
| 0 |  |  |  |  |  |  |  |  |  |  |  | CRITICAL |
| **Self-efficacy** | | | | | | | | | | | | |
| 0 |  |  |  |  |  |  |  |  |  |  |  | CRITICAL |
|  | | | | | | | | | | | | |
|  | | | | | | | | | | | | |
| **Adverse events/harms (high-income country, no leg pain)** | | | | | | | | | | | | |
| 1 | randomised trials | serious^8,w^ | not serious^g^ | serious^h^ | serious^aw^ | none | Authors reported that no TENS-associated adverse events developed in any participants. | | | | ⨁◯◯◯ Very low | CRITICAL |
| **Adverse events/harms stratified by gender** | | | | | | | | | | | | |
| 0 |  |  |  |  |  |  |  |  |  |  |  |  |
| **Adverse events/harms stratified by presence of leg pain** | | | | | | | | | | | | |
| 0 |  |  |  |  |  |  |  |  |  |  |  |  |
| **Adverse events/harms in people in low to lower-middle income countries** | | | | | | | | | | | | |
| 0 |  |  |  |  |  |  |  |  |  |  |  |  |
| **Adverse events/harms stratified by race/ethnicity** | | | | | | | | | | | | |
| 0 |  |  |  |  |  |  |  |  |  |  |  |  |
| **Adverse events/harms stratified by number of treatment sessions** | | | | | | | | | | | | |
| 0 |  |  |  |  |  |  |  |  |  |  |  |  |
| **Social participation** | | | | | | | | | | | | |
| 0 |  |  |  |  |  |  |  |  |  |  |  | CRITICAL |
|  | | | | | | | | | | | | |
| **OLDER ADULTS (aged ≥60 years)** | | | | | | | | | | | | |
| **Pain (follow-up: closest to 2 weeks; assessed with: NRS; benefit indicated by lower values; Scale from: 0 to 10)** | | | | | | | | | | | | |
| 1^r^ | randomised trials | very serious^6,b^ | not serious^g^ | serious^h^ | very serious^t^ | none | 20 | 8 | - | MD **0.13 higher** (9.8 lower to 10.06 higher) | ⨁◯◯◯ Very low | CRITICAL |
| **Pain stratified by gender** | | | | | | | | | | | | |
| 0 |  |  |  |  |  |  |  |  |  |  |  | CRITICAL |
| **Pain stratified by presence of leg pain** | | | | | | | | | | | | |
| 0 |  |  |  |  |  |  |  |  |  |  |  | CRITICAL |
| **Pain stratified by race/ethnicity** | | | | | | | | | | | | |
| 0 |  |  |  |  |  |  |  |  |  |  |  | CRITICAL |
| **Pain stratified by national economic development** | | | | | | | | | | | | |
| 0 |  |  |  |  |  |  |  |  |  |  |  | CRITICAL |
| **Pain stratified by number of treatment sessions** | | | | | | | | | | | | |
| 0 |  |  |  |  |  |  |  |  |  |  |  | CRITICAL |
|  | | | | | | | | | | | | |
| **Function** | | | | | | | | | | | | |
| 0 |  |  |  |  |  |  |  |  |  |  |  | CRITICAL |
| **Health-related quality of life** | | | | | | | | | | | | |
| 0 |  |  |  |  |  |  |  |  |  |  |  | CRITICAL |
| **Depression** | | | | | | | | | | | | |
| 0 |  |  |  |  |  |  |  |  |  |  |  | CRITICAL |
| **Fear avoidance** | | | | | | | | | | | | |
| 0 |  |  |  |  |  |  |  |  |  |  |  | CRITICAL |
| **Catastrophizing** | | | | | | | | | | | | |
| 0 |  |  |  |  |  |  |  |  |  |  |  | CRITICAL |
| **Anxiety** | | | | | | | | | | | | |
| 0 |  |  |  |  |  |  |  |  |  |  |  | CRITICAL |
| **Self-efficacy** | | | | | | | | | | | | |
| 0 |  |  |  |  |  |  |  |  |  |  |  | CRITICAL |
| **Social participation** | | | | | | | | | | | | |
| 0 |  |  |  |  |  |  |  |  |  |  |  | CRITICAL |
| **Change in use of medications** | | | | | | | | | | | | |
| 0 |  |  |  |  |  |  |  |  |  |  |  | CRITICAL |
| **Falls** | | | | | | | | | | | | |
| 0 |  |  |  |  |  |  |  |  |  |  |  | CRITICAL |
| **Adverse events/harms** | | | | | | | | | | | | |
| 0 |  |  |  |  |  |  |  |  |  |  |  | CRITICAL |

**BDI:** Beck Disabiltiy Index; **CI:** confidence interval; **MCS:** Mental Component Summary; **MD:** mean difference; **MPQ:** McGill Pain Questionnaire; **NRS:** numeric rating scale; **ODI:** Oswestry Disability Index; **OIS:** Optimal Information Size; **PCS:** Physical Component Summary; **RMDQ:** Roland-Morris Disability Questionnaire; **SMD:** standardised mean difference; **VAS:** visual analogue scale

The following was used to guide the ratings:

**Risk of bias:** *Not serious:* all or most of the weight (>50%) comes from overall low risk of bias trial(s). *Serious:* some of the weight (<50%) comes from overall low risk of bias trial(s). *Very serious:* all or most of the weight (>50%) comes from overall high or unclear risk of bias trial(s).

**Inconsistency:** *Not serious:* high extent of similarity of point estimates and overlap of confidence intervals; statistical heterogeneity (I^2^) is between 0% and 40%, which might not be important. *Serious:* some extent of similarity of point estimates and overlap of confidence intervals; statistical heterogeneity (I^2^) is between 30% and 60%, which could not be explained due to small subgroups and may represent moderate heterogeneity. *Very serious:* little or no similarity of point estimates and overlap of confidence intervals; statistical heterogeneity (I^2^) is between 50% and 90% or 75% and 100%, which could not be explained due to small subgroups and may represent substantial or considerable heterogeneity, respectively.

**Indirectness:** *Not serious:* trial(s) were conducted in different countries or settings. *Serious:* trial(s) were conducted from a single country/setting. *Very serious:* evidence is not directly related to PICO question.

**Imprecision:** *Not serious:* Optimal Information Size (OIS) was reached (i.e., sample sizes with at least 200 participants per group may provide prognostic balance); and the entire confidence interval lies on one side of the threshold that may be considered clinically important (≥10% scale range or SMD ≥0.2 for continuous variables, ≥10% for binary variables), such that the clinical course of action would not differ if the upper versus the lower boundary of the confidence interval represented the truth. *Serious:* OIS would not have been reached (sample sizes with less than 200 participants per group); if the OIS was reached, the clinical course of action might differ if the upper versus the lower boundary of the confidence interval represented the truth. *Very serious:* similar to ‘serious’ but to a greater extent (e.g., very small sample sizes and confidence intervals crossing appreciable benefit and harm).

**Other considerations:** *Not serious:* Publication bias is undetected. *Serious/very serious:* Publication bias is strongly suspected.

#### Explanations

a. Four trials had 2 arms: Dias 2021 (TENS (GT100Hz) vs. sham, TENS (GT2Hz) vs. sham), Topuz 2004 (conventional TENS vs. sham, low-frequency TENS vs. sham), Yaksi 2021 (burst TENS vs. sham, conventional TENS vs. sham) and Shimoji 2007 (TENS bidirectional modulated sine wave vs. sham, TENS conventional bidirectional pulsed wave vs. sham). For each of these 4 trials we included both arms in meta-analysis and split the comparison groups in half. One trial reporting only p-values was not included in meta-analysis (Bloodworth 2004); results were reported narratively and graded. In this cross-over design, 11 participants with radiculopathy received 4 different TENS interventions and 2 placebo TENS interventions in random order in a single day. Only p-values were provided. Trial authors reported no significant differences between groups (stochastic resonance TENS on back/leg vs. sham, p=0.096; conventional TENS on back/leg vs. sham, p=0.519).

b. Risk of bias: We downgraded twice. Most or all of the trials were rated as overall high risk of bias.

c. Inconsistency: We downgraded once. There is similarity in some of the point estimates with overlapping confidence intervals. Statistical heterogeneity is between 50% and 90% (i.e., I2 = 77%); this could not be explained due to small subgroups and may represent substantial heterogeneity.

d. Indirectness: We did not downgrade. Multiple trials are included from different countries both high- and lower-middle income.

e. Imprecision: We downgraded once due to small sample size (OIS would have not been achieved). The point estimate did not reach the pre-specified threshold for what may be considered appreciable benefit (MD = -1); the confidence interval does not cross the null, but the lower boundary crosses the threshold for what may be considered appreciable benefit (MD = -1).

f. Risk of bias: We downgraded twice due to unclear items related to selection and reporting bias.

g. Inconsistency: We did not downgrade; however, there are no additional trials with which to compare these findings.

h. Indirectness: We downgraded once. This is a single trial from a single centre (high or upper-middle income country).

i. Imprecision: We downgraded twice due to low sample size (the OIS would not have been reached).

j. Imprecision: We downgraded once. The sample size is small (OIS would not have been achieved). The point estimate did not reach the pre-specified threshold for what may be considered appreciable benefit (MD = -1); the confidence interval crossed the null but not the thresholds for what may be considered appreciable benefit (-1) or harm (+1).

k. Inconsistency: We downgraded once. There is similarity in some of the point estimates with overlapping confidence intervals. Statistical heterogeneity is between 50% and 90% (i.e., I2 = 73%); this could not be explained due to small subgroups and may represent substantial heterogeneity.

l. Imprecision: We downgraded once due to small sample size (the OIS would not have been reached). The point estimate reached the pre-specified threshold for what may be considered appreciable benefit (MD = -1). The confidence interval did not cross the null.

m. Inconsistency: We downgraded once. There is similarity in the majority of the point estimates with overlapping confidence intervals. Statistical heterogeneity is between 50% and 90% (i.e., I2 = 74%); this could not be explained due to small subgroups and may represent substantial heterogeneity.

n. Imprecision: We downgraded once. The sample size is small (OIS would not have been achieved). The point estimate did not reach the pre-specified threshold for what may be considered appreciable benefit (MD = -1). The confidence interval crossed the null and the lower boundary crossed the threshold for what may be considered appreciable benefit (MD = -1).

o. These trials had 2 arms each: Dias 2021 (TENS (GT100Hz) vs. sham, TENS (GT2Hz) vs. sham), Topuz 2004 (conventional TENS vs. sham, low-frequency TENS vs. sham).

p. Inconsistency: We did not downgrade. The point estimates are similar with overlapping confidence intervals; statistical heterogeneity is between 0% and 40%, which might not be important (i.e., I2 = 0%).

q. Indirectness: We did not downgrade because the trials were conducted in different countries (high or upper-middle income).

r. Shimoji 2007 conducted 2 trials (TENS bidirectional modulated sine wave vs. sham, TENS conventional bidirectional pulsed wave vs. sham). Both were included in meta-analysis and the comparison group was split in half.

s. Inconsistency: We downgraded twice. The point estimates differ with some overlap in confidence intervals. Statistical heterogeneity is between 50% and 90% (i.e., I2 = 72%); this could not be explained due to small subgroups and may represent substantial heterogeneity.

t. Imprecision: We downgraded twice. The sample size is small (OIS would have not been achieved). The point estimate did not reach the pre-specified threshold for what may be considered clinically important (MD = -1); the confidence interval crosses the null with the lower and upper boundaries crossing the pre-specified thresholds of appreciable benefit (MD = -1) and harm (MD = +1).

u. Four trials had 2 arms: Dias 2021 (TENS (GT100Hz) vs. sham, TENS (GT2Hz) vs. sham), Topuz 2004 (conventional TENS vs. sham, low-frequency TENS vs. sham), Yaksi 2021 (burst TENS vs. sham, conventional TENS vs. sham), and Shimoji 2007. For each of these 4 trials we included both arms in meta-analysis and split the comparison groups in half.

v. Inconsistency: We downgraded once. There is similarity in some of the point estimates with overlapping confidence intervals. Statistical heterogeneity is between 50% and 90% (i.e., I2 = 78%); this could not be explained due to small subgroups and may represent substantial heterogeneity.

w. Risk of bias: We downgraded once due to the potential for selection and performance bias.

x. Indirectness: We downgraded once. This is a single trial from a single centre (low or lower-middle income country).

y. Two trials included 2 arms (Dias 2021: (TENS (GT100Hz) vs. sham, TENS (GT2Hz) vs. sham); and Shimoji 2007. All arms were included in the meta-analyses by splitting the comparison groups in half.

z. Inconsistency: We downgraded twice. Some estimates differ in direction. Statistical heterogeneity is between 50% and 90% (i.e., I2 = 64%); this could not be explained due to small subgroups and may represent substantial heterogeneity.

aa. Two trials had 2 arms each (Topuz 2004: conventional TENS vs. sham, low-frequency TENS vs. sham; Yaksi 2021: burst TENS vs. sham, conventional TENS vs. sham). For each of these 2 trials we included both arms in meta-analysis and split the comparison groups in half.

ab. Inconsistency: We downgraded once. There is similarity in some of the point estimates with overlapping confidence intervals. Statistical heterogeneity is between 50% and 90% (i.e., I2 = 84%); this could not be explained due to small subgroups and may represent substantial heterogeneity.

ac. Risk of bias: We downgraded once. Items were rated as unclear in the selection, performance and reporting domains.

ad. Inconsistency: We downgraded once. There is similarity in some of the point estimates with overlapping confidence intervals. Statistical heterogeneity is between 50% and 90% (i.e., I2 = 70%); this could not be explained due to small subgroups and may represent substantial heterogeneity.

ae. Yaksi 2021 had 2 arms (burst TENS vs. sham, conventional TENS vs. sham); both arms were included in the meta-analysis with the comparison group split in half.

af. Risk of bias: We downgraded twice due to the potential for selection, performance and reporting biases.

ag. Inconsistency: We downgraded once. There is similarity in the point estimates with overlapping confidence intervals. Statistical heterogeneity is between 30% and 60% (i.e., I2 = 50%); this could not be explained due to small subgroups and may represent moderate heterogeneity.

ah. Imprecision: We downgraded twice. The sample size is small (OIS would have not been achieved). The point estimate reached the pre-specified threshold for what may be considered clinically important (MD = -1); the confidence interval crosses the null.

ai. Topuz 2004 had 2 arms (conventional TENS vs. sham, low-frequency TENS vs. sham); both were included in the meta-analysis and the comparison group was split in half.

aj. Inconsistency: We downgraded twice. The results are in different directions with some non-overlapping confidence intervals. Statistical heterogeneity is between 75% and 100% (i.e., I2 = 92%); this could not be explained due to small subgroups and may represent considerable heterogeneity.

ak. Imprecision: We downgraded twice. The sample size is small (OIS would have not been achieved). The point estimate reached the pre-specified threshold for what may be considered clinically important (MD = -0.2); the confidence interval crosses the null.

al. Imprecision: We downgraded twice. The sample size is small (OIS would have not been achieved). The point estimate did not reach the pre-specified threshold for what may be considered clinically important (SMD = -0.2); the confidence interval crosses the null with the lower and upper boundaries crossing the pre-specified thresholds of appreciable benefit (MD = -0.2) and harm (MD = +0.2).

am. Imprecision: We downgraded once due to low sample size (the OIS would not have been reached).

an. Inconsistency: We downgraded once. The point estimates are in different directions with overlapping confidence intervals; statistical heterogeneity is between 0% and 40%, which might not be important (i.e., I2 = 0%).

ao. Imprecision: We downgraded once. The sample size is small (OIS would not have been achieved). The point estimate did not reach the pre-specified threshold for what may be considered appreciable benefit (MD = -5); the confidence interval crossed the null but not the thresholds for what may be considered appreciable benefit (-5) or harm (+5).

ap. Imprecision: We downgraded once. The sample size is small (OIS would not have been achieved). The point estimate did not reach the pre-specified threshold for what may be considered appreciable benefit (MD = -5). The confidence interval crossed the null; the lower boundary crossed the threshold for what may be considered appreciable benefit (-5).

aq. Imprecision: We downgraded twice. The sample size is small (OIS would have not been achieved). The point estimate did not reach the pre-specified threshold for what may be considered clinically important (MD = +10); the confidence interval crosses the null with the lower and upper boundaries crossing the pre-specified thresholds of appreciable benefit (MD = +10) and harm (MD = -10).

ar. Imprecision: We downgraded twice. The sample size is small (OIS would have not been achieved). The point estimate reached the pre-specified threshold for what may be considered clinically important favouring the comparison (MD = -10); the confidence interval crossed the null.

as. Imprecision: We downgraded once. The sample size is small (OIS would not have been achieved). The point estimate did not reach the pre-specified threshold for what may be considered appreciable benefit (MD = +10); the confidence interval crossed the null.

at. Inconsistency: We downgraded twice. The point estimates differ in direction and the confidence intervals do not overlap. Statistical heterogeneity is between 50% and 90% (i.e., I2 = 87%); this could not be explained due to small subgroups and may represent substantial heterogeneity.

au. Imprecision: We downgraded once. The sample size was small (OIS would not have been reached). The pointe estimate reached the threshold for what may be considered clinically important favouring the comparison (MD = -10); the confidence interval did not cross the null.

av. Imprecision: We downgraded twice. The sample size is small (OIS would have not been achieved). The point estimate did not reach the pre-specified threshold for what may be considered clinically important (MD = -6.3). The confidence interval crosses the null with the lower and upper boundaries crossing the pre-specified thresholds of appreciable benefit (MD = -6.3) and harm (MD = +6.3).

aw. Imprecision: We downgraded once due to low sample size (the OIS would not have been reached).

#### References

1.Cheing GL, Hui-Chan CW. Transcutaneous electrical nerve stimulation: nonparallel antinociceptive effects on chronic clinical pain and acute experimental pain. Arch Phys Med Rehabil; 1999.

2.de Alencar Caldas VV, Maciel DG, Cerqueira MS, et al. Effect of Pain Education, Cryotherapy, and Transcutaneous Electrical Nerve Stimulation on the Pain, Functional Capacity, and Quality of Life in Patients With Nonspecific Chronic Low Back Pain: A Single-Blind Randomized Controlled Trial. Am J Phys Med Rehabil; 2021.

3.Dias LV, Cordeiro MA, Schmidt de Sales R, et al. Immediate analgesic effect of transcutaneous electrical nerve stimulation (TENS) and interferential current (IFC) on chronic low back pain: Randomised placebo-controlled trial. Journal of Bodywork & Movement Therapies; 2021.

4.Ezema CI, Onyeso OK,Nna EO,et al. Transcutaneous electrical nerve stimulation effects on pain-intensity and endogenous opioids levels among chronic low-back pain patients: A randomised controlled trial. J Back Musculoskelet Rehabil; 2022.

5.Kofotolis ND, Vlachopoulos SP,Kellis E. Sequentially allocated clinical trial of rhythmic stabilization exercises and TENS in women with chronic low back pain. Clin Rehabil; 2008.

6.Shimoji K, Takahashi N,Nishio Y,Koyanagi M,Aida S. Pain relief by transcutaneous electric nerve stimulation with bidirectional modulated sine waves in patients with chronic back pain: a randomized, double-blind, sham-controlled trial. Neuromodulation; 2007.

7.Topuz O, Ozfidan E,Ozgen M,Ardic F. Efficacy of transcutaneous electrical nerve stimulation and percutaneous neuromodulation therapy in chronic low back pain. Journal of Back and Musculoskeletal Rehabilitation; 2004.

8.Yaksi E, Ketenci A, Baslo MB, Orhan EK. Does transcutaneous electrical nerve stimulation affect pain, neuropathic pain, and sympathetic skin responses in the treatment of chronic low back pain? A randomized, placebo-controlled trial. Korean J Pain; 2021.

9.Bloodworth DM, Nguyen BN,Garver W,et al. Comparison of stochastic vs. conventional transcutaneous electrical stimulation for pain modulation in patients with electromyographically documented radiculopathy. Am J Phys Med Rehabil; 2004.

10.Kibar S, Konak HE,Ay S,Doganay Erdogan B,Evcik D. The effectiveness of combined transcutaneous electrical nerve stimulation and interferential current therapy on chronic low back pain: a randomized, double-blind, sham-controlled trial. Fiziksel Tip ve Rehabilitasyon Bilimleri Dergisi [Journal of Physical Medicine and Rehabilitation Sciences]; 2020.

GRADE Evidence Profile table 2: ***What are the benefits and harms of TENS in the management of community-dwelling adults (including older adults aged 60 years and over) with chronic primary low back pain (with or without leg pain) compared to no treatment or treatments where the effect of TENS could be isolated?***

| **Certainty assessment** | | | | | | | **№ of patients** | | **Effect** | | **Certainty** | **Importance** |
| --- | --- | --- | --- | --- | --- | --- | --- | --- | --- | --- | --- | --- |
| **№ of trials** | **Trial design** | **Risk of bias** | **Inconsistency** | **Indirectness** | **Imprecision** | **Other considerations** | **TENS** | **Nno treatment** | **Relative (95% CI)** | **Absolute (95% CI)** |  |  |
| **ALL ADULTS** | | | | | | | | | | | | |
| **Pain (follow-up: closest to 2 weeks; assessed with: VAS, NRS, Borg scale; benefit indicated by lower values; Scale from: 0 to 10)** | | | | | | | | | | | | |
| 8 | randomised trials | very serious^1,2,3,4,5,6,7,8,a,b^ | not serious^c^ | not serious^d^ | serious^e^ | none | 192 | 146 | - | MD **0.19 lower** (0.51 lower to 0.14 higher) | ⨁◯◯◯ Very low | CRITICAL |
| **Pain in females and males (follow-up: closest to 2 weeks; assessed with: VAS, NRS, Borg scale; benefit indicated by lower values; Scale from: 0 to 10)** | | | | | | | | | | | | |
| 7 | randomised trials | very serious^1,2,3,4,5,7,8,b^ | not serious^c^ | not serious^d^ | serious^f^ | none | 171 | 123 | - | MD **0.35 lower** (0.66 lower to 0.03 lower) | ⨁◯◯◯ Very low | CRITICAL |
| **Pain in females (follow-up: closest to 2 weeks; assessed with: Borg scale; benefit indicated by lower values; Scale from: 0 to 10)** | | | | | | | | | | | | |
| 1 | randomised trials | very serious^6,b^ | not serious^g^ | serious^h^ | serious^e^ | none | 21 | 23 | - | MD **0.2 higher** (0.07 lower to 0.47 higher) | ⨁◯◯◯ Very low | CRITICAL |
|  | | | | | | | | | | | | |
|  | | | | | | | | | | | | |
| **Pain in people without leg pain (follow-up: closest to 2 weeks; assessed with: VAS, Borg scale; benefit indicated by lower values; Scale from: 0 to 10)** | | | | | | | | | | | | |
| 4 | randomised trials | very serious^2,6,7,8,a,b^ | not serious^i^ | not serious^d^ | serious^e^ | none | 122 | 79 | - | MD **0**  (0.42 lower to 0.41 higher) | ⨁◯◯◯ Very low | CRITICAL |
| **Pain in people with unclassified presence of leg pain (follow-up: closest to 2 weeks; assessed with: VAS, NRS, Borg scale; benefit indicated by lower values; Scale from: 0 to 10)** | | | | | | | | | | | | |
| 2 | randomised trials | very serious^1,3,b^ | not serious^i^ | not serious^d^ | serious^j^ | none | 27 | 27 | - | MD **0.18 higher** (0.12 higher to 0.24 higher) | ⨁◯◯◯ Very low | CRITICAL |
| **Pain in people with and without leg pain (radicular or non-radicular) (follow-up: closest to 2 weeks; assessed with: VAS, NRS; benefit indicated by lower values; Scale from: 0 to 10)** | | | | | | | | | | | | |
| 2 | randomised trials | very serious^4,5,b^ | serious^k^ | not serious^l^ | very serious^m^ | none | 43 | 40 | - | MD **0.48 lower** (5.31 lower to 4.35 higher) | ⨁◯◯◯ Very low | CRITICAL |
| **Pain in trials undertaken in high to upper-middle income countries (follow-up: closest to 2 weeks; assessed with: VAS, NRS, Borg scale; benefit indicated by lower values; Scale from: 0 to 10)** | | | | | | | | | | | | |
| 6 | randomised trials | very serious^1,4,5,6,7,8,b^ | not serious^n^ | not serious^l^ | serious^e^ | none | 151 | 120 | - | MD **0.15 lower** (0.49 lower to 0.19 higher) | ⨁◯◯◯ Very low | CRITICAL |
|  | | | | | | | | | | | | |
| **Pain in trials undertaken in low to lower-middle income countries (follow-up: closest to 2 weeks; assessed with: VAS, NRS; benefit indicated by lower values; Scale from: 0 to 10)** | | | | | | | | | | | | |
| 2 | randomised trials | very serious^2,3,b,o^ | not serious^p^ | not serious^q^ | very serious^m^ | none | 41 | 26 | - | MD **0.53 lower** (3 lower to 1.95 higher) | ⨁◯◯◯ Very low | CRITICAL |
| **Pain in trials using 10-20 TENS treatment sessions (follow-up: closest to 2 weeks; assessed with: VAS, NRS, Borg scale; benefit indicated by lower values; Scale from: 0 to 10)** | | | | | | | | | | | | |
| 6 | randomised trials | very serious^2,3,4,5,6,7,b,o^ | not serious^r^ | not serious^d^ | serious^e^ | none | 116 | 100 | - | MD **0.21 lower** (0.72 lower to 0.29 higher) | ⨁◯◯◯ Very low | CRITICAL |
| **Pain in trials using <10 TENS treatment sessions (follow-up: closest to 2 weeks; assessed with: VAS; benefit indicated by lower values; Scale from: 0 to 10)** | | | | | | | | | | | | |
| 2^s^ | randomised trials | very serious^1,8,b^ | not serious^i^ | not serious^l^ | serious^e^ | none | 76 | 46 | - | MD **0.04 higher** (0.3 lower to 0.38 higher) | ⨁◯◯◯ Very low | CRITICAL |
| **Pain (high-income country) (follow-up: closest to 3 months; assessed with: Brief Pain Inventory, Borg scale; benefit indicated by lower values; Scale from: 0 to 10)** | | | | | | | | | | | | |
| 2 | randomised trials | very serious^6,9,t,u^ | very serious^v^ | not serious^l^ | very serious^m^ | none | 50 | 54 | - | MD **0.98 lower** (16.83 lower to 14.88 higher) | ⨁◯◯◯ Very low | CRITICAL |
|  | | | | | | | | | | | | |
| **Pain (females and males, either with or without radicular or non-radicular leg pain) (follow-up: closest to 3 months; assessed with: Brief Pain Inventory; benefit indicated by lower values; Scale from: 0 to 10)** | | | | | | | | | | | | |
| 1 | randomised trials | serious^9,t^ | not serious^g^ | serious^h^ | serious^w^ | none | 29 | 31 | - | MD **2.3 SD lower** (3.51 lower to 1.09 lower) | ⨁◯◯◯ Very low | CRITICAL |
| **Pain (females, no leg pain) (follow-up: closest to 3 months; assessed with: Borg Scale; benefit indicated by lower values; Scale from: 0 to 10)** | | | | | | | | | | | | |
| 1 | randomised trials | very serious^6,b^ | not serious^g^ | serious^h^ | serious^f^ | none | 21 | 23 | - | MD **0.2 higher** (0.01 lower to 0.41 higher) | ⨁◯◯◯ Very low | CRITICAL |
| **Pain in people in low to lower-middle income countries (follow-up: closest to 3 months)** | | | | | | | | | | | | |
| 0 |  |  |  |  |  |  |  |  |  |  |  | CRITICAL |
| **Pain stratified by race/ethnicity** | | | | | | | | | | | | |
| 0 |  |  |  |  |  |  |  |  |  |  |  | CRITICAL |
| **Pain stratified by number of treatment sessions (follow-up: closest to 3 months)** | | | | | | | | | | | | |
| 0 |  |  |  |  |  |  |  |  |  |  |  | CRITICAL |
|  | | | | | | | | | | | | |
|  | | | | | | | | | | | | |
| **Function (follow-up: closest to 2 weeks; assessed with: ODI, RMDQ; benefit indicated by lower values)** | | | | | | | | | | | | |
| 6 | randomised trials | very serious^1,2,3,4,7,10,b,o^ | not serious^x^ | not serious^d^ | serious^y^ | none | 108 | 91 | - | SMD **0.32 lower** (0.71 lower to 0.07 higher) | ⨁◯◯◯ Very low | CRITICAL |
| **Function in females (follow-up: closest to 2 weeks; assessed with: modified ODI; benefit indicated by lower values)** | | | | | | | | | | | | |
| 1 | randomised trials | very serious^10,b^ | not serious^g^ | serious^z^ | very serious^aa^ | none | 8 | 8 | - | SMD **0.29 lower** (1.28 lower to 0.69 higher) | ⨁◯◯◯ Very low | CRITICAL |
| **Function in females and males (follow-up: closest to 2 weeks; assessed with: ODI, RMDQ; benefit indicated by lower values)** | | | | | | | | | | | | |
| 5 | randomised trials | very serious^1,2,3,4,7,b^ | not serious^ab^ | not serious^d^ | serious^y^ | none | 100 | 83 | - | SMD **0.32 lower** (0.78 lower to 0.15 higher) | ⨁◯◯◯ Very low | CRITICAL |
| **Function in people without leg pain (follow-up: closest to 2 weeks; assessed with: ODI, RMDQ; benefit indicated by lower values)** | | | | | | | | | | | | |
| 3 | randomised trials | very serious^2,7,10,b,o^ | not serious^i^ | not serious^d^ | serious^ac^ | none | 49 | 34 | - | SMD **0.15 lower** (0.37 lower to 0.08 higher) | ⨁◯◯◯ Very low | CRITICAL |
|  | | | | | | | | | | | | |
| **Function in people with unclassified presence of leg pain (follow-up: closest to2 weeks; assessed with: ODI, RMDQ; benefit indicated by lower values)** | | | | | | | | | | | | |
| 2 | randomised trials | very serious^b,o^ | not serious^i^ | not serious^d^ | very serious^ad^ | none | 27 | 27 | - | SMD **0.08 lower** (0.74 lower to 0.58 higher) | ⨁◯◯◯ Very low | CRITICAL |
| **Function in people either with or without radicular leg pain (follow-up: closest to 2 weeks; assessed with: RMDQ; benefit indicated by lower values)** | | | | | | | | | | | | |
| 1 | randomised trials | very serious^4,b^ | not serious^g^ | serious^h^ | serious^w^ | none | 32 | 30 | - | SMD **1.03 lower** (1.56 lower to 0.49 lower) | ⨁◯◯◯ Very low | CRITICAL |
| **Function in trials undertaken in low to lower-middle income countries (follow-up: closest to 2 weeks; assessed with: ODI, RMDQ; benefit indicated by lower values)** | | | | | | | | | | | | |
| 3 | randomised trials | very serious^2,3,10,b,o^ | not serious^i^ | not serious^q^ | serious^ae^ | none | 49 | 34 | - | SMD **0.16 lower** (0.36 lower to 0.03 higher) | ⨁◯◯◯ Very low | CRITICAL |
| **Function in trials undertaken in high to upper-middle income countries (follow-up: closest to 2 weeks; assessed with: RMDQ; benefit indicated by lower values)** | | | | | | | | | | | | |
| 3 | randomised trials | very serious^1,4,7,b^ | serious^af^ | not serious^ag^ | very serious^aa^ | none | 59 | 57 | - | SMD **0.47 lower** (1.94 lower to 1 higher) | ⨁◯◯◯ Very low | CRITICAL |
|  | | | | | | | | | | | | |
| **Function in trials using 10-20 TENS treatment sessions (follow-up: closest to 2 weeks; assessed with: ODI, RMDQ; benefit indicated by lower values)** | | | | | | | | | | | | |
| 5 | randomised trials | very serious^2,3,4,7,10,b,o^ | not serious^ah^ | not serious^d^ | serious^ai^ | none | 92 | 75 | - | SMD **0.35 lower** (0.82 lower to 0.12 higher) | ⨁◯◯◯ Very low | CRITICAL |
| **Function in trials using <10 treatment sessions (follow-up: closest to 2 weeks; assessed with: RMDQ; benefit indicated by lower values; Scale from: 0 to 24)** | | | | | | | | | | | | |
| 1 | randomised trials | very serious^1,aj,b^ | not serious^g^ | serious^h^ | very serious^ad^ | none | 16 | 16 | - | SMD **0.12 lower** (0.82 lower to 0.57 higher) | ⨁◯◯◯ Very low | CRITICAL |
| **Function (high-income country) (follow-up: closest to 3 months; assessed with: ODI, PDI; benefit indicated by lower values)** | | | | | | | | | | | | |
| 2 | randomised trials | very serious^6,9,b^ | very serious^ak^ | serious^h^ | very serious^aa^ | none | 50 | 54 | - | SMD **1.05 higher** (18.51 lower to 20.61 higher) | ⨁◯◯◯ Very low | CRITICAL |
| **Function (females, no leg pain) (follow-up: closest to 3 months; assessed with: ODI; benefit indicated by lower values)** | | | | | | | | | | | | |
| 1 | randomised trials | very serious^6,b^ | not serious^g^ | serious^h^ | serious^w^ | none | 21 | 23 | - | SMD **2.6 higher** (1.78 higher to 3.42 higher) | ⨁◯◯◯ Very low | CRITICAL |
| **Function (females and males, either with or without radicular or non-radicular leg pain) (follow-up: closest to 3 months; assessed with: PDI; benefit indicated by lower values)** | | | | | | | | | | | | |
| 1 | randomised trials | serious^9,t^ | not serious^g^ | serious^h^ | serious^y^ | none | 29 | 31 | - | SMD **0.48 lower** (0.99 lower to 0.04 higher) | ⨁◯◯◯ Very low | CRITICAL |
| **Function stratified by race/ethnicity** | | | | | | | | | | | | |
| 0 |  |  |  |  |  |  |  |  |  |  |  | CRITICAL |
| **Health-related quality of life (no leg pain, high-income country) (follow-up: closest to 2 weeks; assessed with: SF-36 (PCS); benefit indicated by higher values; Scale from: 0 to 100)** | | | | | | | | | | | | |
| 1 | randomised trials | very serious^7,b^ | not serious^g^ | serious^h^ | very serious^al^ | none | 11 | 11 | - | MD **6.82 lower** (27.06 lower to 13.42 higher) | ⨁◯◯◯ Very low | CRITICAL |
| **Health-related quality of life (no leg pain, high-income country) (follow-up: closest to 2 weeks; assessed with: SF-36 (MCS); benefit indicated by higher values; Scale from: 0 to 100)** | | | | | | | | | | | | |
| 1 | randomised trials | very serious^7,b^ | not serious^g^ | serious^h^ | serious^am^ | none | 11 | 11 | - | MD **2.91 lower** (10.25 lower to 4.43 higher) | ⨁◯◯◯ Very low | CRITICAL |
| **Health-related quality of life stratified by gender** | | | | | | | | | | | | |
| 0 |  |  |  |  |  |  |  |  |  |  |  | CRITICAL |
| **Health-related quality of life stratified by presence of leg pain** | | | | | | | | | | | | |
| 0 |  |  |  |  |  |  |  |  |  |  |  | CRITICAL |
| **Health-related quality of life stratified by race/ethnicity** | | | | | | | | | | | | |
| 0 |  |  |  |  |  |  |  |  |  |  |  | CRITICAL |
| **Health-related quality of life in trials undertaken in low to lower-middle income countries** | | | | | | | | | | | | |
| 0 |  |  |  |  |  |  |  |  |  |  |  | CRITICAL |
| **Depression (either with or without radicular or non-radicular leg pain, high-income country) (follow-up: closest to 3 months; assessed with: HADS; benefit indicated by lower values; Scale from: 0 to 21)** | | | | | | | | | | | | |
| 1 | randomised trials | serious^9,t^ | not serious^g^ | serious^h^ | very serious^an^ |  | 29 | 31 | - | MD **1.4 lower** (5.57 lower to 2.77 higher) | - | CRITICAL |
| **Depression stratified by gender** | | | | | | | | | | | | |
| 0 |  |  |  |  |  |  |  |  |  |  |  | CRITICAL |
| **Depression stratified by presence of leg pain** | | | | | | | | | | | | |
| 0 |  |  |  |  |  |  |  |  |  |  |  | CRITICAL |
| **Depression stratified by race/ethnicity** | | | | | | | | | | | | |
| 0 |  |  |  |  |  |  |  |  |  |  |  | CRITICAL |
| **Depression in people in low to lower-middle income countries** | | | | | | | | | | | | |
| 0 |  |  |  |  |  |  |  |  |  |  |  | CRITICAL |
| **Catastrophizing (either with or without radicular or non-radicular leg pain, high-income country) (follow-up: closest to 3 months; assessed with: Pain Catastrophizing Scale; benefit indicated by lower values; Scale from: 0 to 52)** | | | | | | | | | | | | |
| 1 | randomised trials | serious^9,t^ | not serious^g^ | serious^h^ | serious^w^ | none | 29 | 31 | - | MD **11.2 lower** (17.88 lower to 4.52 lower) | ⨁◯◯◯ Very low | CRITICAL |
| **Catastrophizing stratified by gender** | | | | | | | | | | | | |
| 0 |  |  |  |  |  |  |  |  |  |  |  | CRITICAL |
| **Catastrophizing stratified by presence of leg pain** | | | | | | | | | | | | |
| 0 |  |  |  |  |  |  |  |  |  |  |  | CRITICAL |
| **Catastrophizing stratified by race/ethnicity** | | | | | | | | | | | | |
| 0 |  |  |  |  |  |  |  |  |  |  |  | CRITICAL |
| **Catastrophizing in people in low to lower-middle income countries** | | | | | | | | | | | | |
| 0 |  |  |  |  |  |  |  |  |  |  |  | CRITICAL |
| **Fear avoidance** | | | | | | | | | | | | |
| 0 |  |  |  |  |  |  |  |  |  |  |  | CRITICAL |
| **Anxiety** | | | | | | | | | | | | |
| 0 |  |  |  |  |  |  |  |  |  |  |  | CRITICAL |
| **Self-efficacy** | | | | | | | | | | | | |
| 0 |  |  |  |  |  |  |  |  |  |  |  | CRITICAL |
| **Social participation** | | | | | | | | | | | | |
| 0 |  |  |  |  |  |  |  |  |  |  |  | CRITICAL |
| **Adverse events/harms (high-income country, either with or without leg pain (radicular or non-radicular)** | | | | | | | | | | | | |
| 1 | randomised trials | serious^t^ | not serious^g^ | serious^h^ | serious^w^ | none | Authors reported that none of the participants reported experiencing any long-term adverse events from using high-frequency TENS. | | | | ⨁◯◯◯ Very low | CRITICAL |
| **Adverse events/harms stratified by gender** | | | | | | | | | | | | |
| 0 |  |  |  |  |  |  |  |  |  |  |  |  |
| **Adverse events/harms in people in low to lower-middle income countries** | | | | | | | | | | | | |
| 0 |  |  |  |  |  |  |  |  |  |  |  |  |
| **Adverse events/harms stratified by race/ethnicity** | | | | | | | | | | | | |
| 0 |  |  |  |  |  |  |  |  | - | **0**  (0 to 0 ) | - |  |
| **OLDER ADULTS (aged ≥60 years)** | | | | | | | | | | | | |
| **Pain** | | | | | | | | | | | | |
| 0 |  |  |  |  |  |  |  |  |  |  |  | CRITICAL |
| **Function** | | | | | | | | | | | | |
| 0 |  |  |  |  |  |  |  |  |  |  |  | CRITICAL |
| **Health-related quality of life** | | | | | | | | | | | | |
|  |  |  |  |  |  |  |  |  |  |  |  | CRITICAL |
| **Depression** | | | | | | | | | | | | |
| 0 |  |  |  |  |  |  |  |  |  |  |  | CRITICAL |
| **Fear avoidance** | | | | | | | | | | | | |
| 0 |  |  |  |  |  |  |  |  |  |  |  | CRITICAL |
| **Catastrophizing** | | | | | | | | | | | | |
| 0 |  |  |  |  |  |  |  |  |  |  |  | CRITICAL |
| **Anxiety** | | | | | | | | | | | | |
| 0 |  |  |  |  |  |  |  |  |  |  |  | CRITICAL |
|  | | | | | | | | | | | | |
| **Self-efficacy** | | | | | | | | | | | | |
| 0 |  |  |  |  |  |  |  |  |  |  |  | CRITICAL |
| **Social participation** | | | | | | | | | | | | |
| 0 |  |  |  |  |  |  |  |  |  |  |  | CRITICAL |
| **Adverse events** | | | | | | | | | | | | |
| 0 |  |  |  |  |  |  |  |  |  |  |  | CRITICAL |
| **Change in use of medications** | | | | | | | | | | | | |
| 0 |  |  |  |  |  |  |  |  |  |  |  | CRITICAL |
| **Falls** | | | | | | | | | | | | |
| 0 |  |  |  |  |  |  |  |  |  |  |  | CRITICAL |

#### BDI: Beck Disabiltiy Index; CI: confidence interval; MCS: Mental Component Summary; MD: mean difference; MPQ: McGill Pain Questionnaire; NRS: numeric rating scale; ODI: Oswestry Disability Index; OIS: Optimal Information Size; PCS: Physical Component Summary; PDI: Pain Disability Index; RMDQ: Roland-Morris Disability Questionnaire; SMD: standardised mean difference; VAS: visual analogue scale

The following was used to guide the ratings:

**Risk of bias:** *Not serious:* all or most of the weight (>50%) comes from overall low risk of bias trial(s). *Serious:* some of the weight (<50%) comes from overall low risk of bias trial(s). *Very serious:* all or most of the weight (>50%) comes from overall high or unclear risk of bias trial(s).

**Inconsistency:** *Not serious:* high extent of similarity of point estimates and overlap of confidence intervals; statistical heterogeneity (I^2^) is between 0% and 40%, which might not be important. *Serious:* some extent of similarity of point estimates and overlap of confidence intervals; statistical heterogeneity (I^2^) is between 30% and 60%, which could not be explained due to small subgroups and may represent moderate heterogeneity. *Very serious:* little or no similarity of point estimates and overlap of confidence intervals; statistical heterogeneity (I^2^) is between 50% and 90% or 75% and 100%, which could not be explained due to small subgroups and may represent substantial or considerable heterogeneity, respectively.

**Indirectness:** *Not serious:* trial(s) were conducted in different countries or settings. *Serious:* trial(s) were conducted from a single country/setting. *Very serious:* evidence is not directly related to PICO question.

**Imprecision:** *Not serious:* Optimal Information Size (OIS) was reached (i.e., sample sizes with at least 200 participants per group may provide prognostic balance); and the entire confidence interval lies on one side of the threshold that may be considered clinically important (≥10% scale range or SMD ≥0.2 for continuous variables, ≥10% for binary variables), such that the clinical course of action would not differ if the upper versus the lower boundary of the confidence interval represented the truth. *Serious:* OIS would not have been reached (sample sizes with less than 200 participants per group); if the OIS was reached, the clinical course of action might differ if the upper versus the lower boundary of the confidence interval represented the truth. *Very serious:* similar to ‘serious’ but to a greater extent (e.g., very small sample sizes and confidence intervals crossing appreciable benefit and harm).

**Other considerations:** *Not serious:* Publication bias is undetected. *Serious/very serious:* Publication bias is strongly suspected.

#### Explanations

a. Elserty 2016 included 2 arms (fixed pulse TENS + exercise vs. exercise; adjusted pulse TENS + exercise vs. exercise). Both were included in meta-analysis by splitting the comparison group number in half. Petrofsky 2020 included 4 arms (Continuous TENS + spent sham heat vs. spent sham heat; continuous TENS + LLCH (low-level continuous heat) vs. LLCH; TENS last 15 min + LLCH vs. LLCH; TENS last 15 min + spent sham heat vs. spent sham heat). All were included in meta-analysis by splitting the comparison group numbers accordingly.

b. Risk of bias: We downgraded twice. Most or all of the trials were rated as overall high risk of bias.

c. Inconsistency: We did not downgrade. The point estimates are similar with overlapping confidence intervals; statistical heterogeneity is between 0% and 40%, which might not be important (i.e., I2 = 6%).

d. Indirectness: We did not downgrade. Trials are included from different countries both high- and lower-middle income.

e. Imprecision: We downgraded once. The sample size is small (OIS would not have been achieved). The point estimate did not reach the pre-specified threshold for what may be considered appreciable benefit (MD = -1). The confidence interval crossed the null but not the thresholds for what may be considered appreciable benefit (-1) or harm (+1).

f. Imprecision: We downgraded once. The sample size was small (OIS would not have been reached). The point estimate did not reach the pre-specified threshold for what may be considered clinically important (MD = -1); the confidence interval did not cross the null.

g. Inconsistency: We did not downgrade; however, there are no additional trials with which to compare these findings.

h. Indirectness: We downgraded once. This is a single trial from a single centre (high or upper-middle income country).

i. Inconsistency: We did not downgrade. The point estimates are similar with overlapping confidence intervals; statistical heterogeneity is between 0% and 40%, which might not be important (i.e., I2 = 0%).

j. Imprecision: We downgraded once. The sample size was small (OIS would not have been reached). The point estimate did not reach the threshold for what may be considered clinically important (MD = -1); the confidence interval did not cross the null.

k. Inconsistency: We downgraded once. The point estimates are close with some overlap in the confidence intervals. Statistical heterogeneity is between 50% and 90% (i.e., I2 = 65%). This could not be explained due to small subgroups and may represent substantial heterogeneity.

l. Indirectness: We did not downgrade because the trials were conducted in different countries (high or upper-middle income).

m. Imprecision: We downgraded twice. The sample size is small (OIS would have not been achieved). The point estimate did not reach the pre-specified threshold for what may be considered clinically important (MD = -1); the confidence interval crosses the null with the lower and upper boundaries crossing the pre-specified thresholds of appreciable benefit (MD = -1) and harm (MD = +1).

n. Inconsistency: We did not downgrade. The point estimates are similar with overlapping confidence intervals; statistical heterogeneity is between 0% and 40%, which might not be important (i.e., I2 = 10%).

o. Elserty 2016 included 2 arms (fixed pulse TENS + exercise vs. exercise; adjusted pulse TENS + exercise vs. exercise). Both were included in meta-analysis by splitting the comparison group number in half.

p. Inconsistency: We did not downgrade. The point estimates differ in direction, but the confidence intervals overlap; statistical heterogeneity is between 0% and 40%, which might not be important (i.e., I2 = 4%).

q. Indirectness: We did not downgrade because the trials were conducted in different countries (low or lower-middle income).

r. Inconsistency: We did not downgrade. The point estimates are similar with overlapping confidence intervals; statistical heterogeneity is between 30% and 60%, which may represent moderate heterogeneity (i.e., I2 = 48%).

s. Depaoli Lemos 2021 used 4 TENS sessions; Petrofsky 2020 used a single TENS session.

t. Risk of bias: We downgraded once due to the potential for selection, performance and other biases.

u. Kofotolis and Jamison: Participants had 20-90 treatment sessions.

v. Inconsistency: We downgraded twice. The point estimates were in different directions with little to no overlap in confidence intervals. Statistical heterogeneity is between 75% and 100% (i.e., I2 = 94%). This could not be explained due to small subgroups and may represent considerable heterogeneity.

w. Imprecision: We downgraded once due to small sample size (the OIS would not have been reached).

x. Inconsistency: We did not downgrade. Most of the point estimates are similar with overlapping confidence intervals; statistical heterogeneity is between 0% and 40%, which might not be important (i.e., I2 = 28%).

y. Imprecision: We downgraded once due to small sample size (the OIS would not have been reached). The point estimate reached the pre-specified threshold for what may be considered appreciable benefit (SMD = -0.2). The confidence interval crossed the null.

z. Indirectness: We downgraded once. This is a single trial from (low or lower-middle income country).

aa. Imprecision: We downgraded twice. The sample size is small (OIS would have not been achieved). The point estimate reached the pre-specified threshold for what may be considered clinically important (SMD = -0.2); the confidence interval crosses the null.

ab. Inconsistency: We did not downgrade. Most of the point estimates are similar with overlapping confidence intervals; statistical heterogeneity is between 0% and 40%, which might not be important (i.e., I2 = 39%).

ac. Imprecision: We downgraded once. The sample size is small (OIS would have not been achieved). The point estimate did not reach the pre-specified threshold for what may be considered clinically important (SMD = -0.2). The confidence interval crossed the null, but the upper boundary did not cross the pre-specified threshold for what may be considered appreciable harm (SMD = +0.2).

ad. Imprecision: We downgraded twice. The sample size is small (OIS would have not been achieved). The point estimate did not reach the pre-specified threshold for what may be considered clinically important (SMD = -0.2); the confidence interval crosses the null with the lower and upper boundaries crossing the pre-specified thresholds for what may be considered appreciable benefit (SMD = -0.2) and harm (SMD = +0.2).

ae. Imprecision: We downgraded once due to small sample size (the OIS would not have been reached). The point estimate did not reach the pre-specified threshold for what may be considered appreciable benefit (SMD = -0.2). The confidence interval crossed the null.

af. Inconsistency: We downgraded once. There was some difference in magnitude and direction of the point estimates, but there was some overlap in confidence intervals. Statistical heterogeneity is between 50% and 90% (i.e., I2 = 69%). This could not be explained due to small subgroups and may represent substantial heterogeneity.

ag. We did not downgrade because the trials were conducted in different countries.

ah. Inconsistency: We did not downgrade. Most of the point estimates are similar with overlapping confidence intervals; statistical heterogeneity is between 0% and 40%, which might not be important (i.e., I2 = 39%).

ai. Imprecision: We downgraded once due to small sample size (the OIS would not have been reached). The point estimate reached the pre-specified threshold for what may be considered appreciable benefit (SMD = -0.2). The confidence interval crossed the null, but the upper boundary did not cross the pre-specified threshold for what may be considered appreciable harm (SMD = +0.2).

aj. Depaoli Lemos 2021 used 4 TENS sessions.

ak. Inconsistency: We downgraded twice. The point estimates were in different directions with little to no overlap in confidence intervals. Statistical heterogeneity is between 75% and 100% (i.e., I2 = 97%). This could not be explained due to small subgroups and may represent considerable heterogeneity.

al. Imprecision: We downgraded twice due to small sample size (the OIS would not have been reached). The point estimate did not reach the pre-specified threshold for what may be considered appreciable harm (-10). The confidence interval crossed the null with the boundaries crossing the pre-specified thresholds for what may be considered appreciable harm (-10) and benefit (+10).

am. Imprecision: We downgraded once due to small sample size (the OIS would not have been reached). The point estimate did not reach the pre-specified threshold for what may be considered appreciable harm (MD = -10). The confidence interval crossed the null.

an. Imprecision: We downgraded twice due to small sample size (the OIS would not have been reached). The point estimate did not reach the pre-specified threshold for what may be considered appreciable benefit (MD = -2.1). The confidence interval crossed the null with the boundaries crossing the pre-specified thresholds for what may be considered appreciable benefit (-2.1) or harm (+2.1).

#### References

1.Depaoli Lemos VJ, Selau RC,Blos C,Baptista Dohnert M,Boff Daitx R,de Almeida Brito V. Electroacupuncture and Transcutaneous Electrical Nerve Stimulation in Chronic Nonspecific Low Back Pain: a Blind Randomized Clinical Trial. Muscles, ligaments & tendons journal; 2021.

2.Elserty N, Kattabei O,Elhafez H.. Effect of Fixed Versus Adjusted Transcutaneous Electrical Nerve Stimulation Amplitude on Chronic Mechanical Low Back Pain. J Altern Complement Med; 2016.

3.Tella BA, Oghumu SN,Gbiri CAO. Efficacy of transcutaneous electrical nerve stimulation and interferential current on tactile acuity of individuals with nonspecific chronic low back pain. Neuromodulation; 2021.

4.Kibar S, Konak HE, Ay S, Doganay Erdogan B,Evcik D. The effectiveness of combined transcutaneous electrical nerve stimulation and interferential current therapy on chronic low back pain: a randomized, double-blind, sham-controlled trial. Fiziksel Tip ve Rehabilitasyon Bilimleri Dergisi [Journal of Physical Medicine and Rehabilitation Sciences]; 2020.

5.Shimoji K, Takahashi N,Nishio Y,Koyanagi M,Aida S. Pain relief by transcutaneous electric nerve stimulation with bidirectional modulated sine waves in patients with chronic back pain: a randomized, double-blind, sham-controlled trial. Neuromodulation; 2007.

6.Kofotolis ND, Vlachopoulos SP,Kellis E. Sequentially allocated clinical trial of rhythmic stabilization exercises and TENS in women with chronic low back pain. Clin Rehabil; 2008.

7.de Alencar Caldas VV, Maciel DG, Cerqueira MS, et al. Effect of Pain Education, Cryotherapy, and Transcutaneous Electrical Nerve Stimulation on the Pain, Functional Capacity, and Quality of Life in Patients With Nonspecific Chronic Low Back Pain: A Single-Blind Randomized Controlled Trial. Am J Phys Med Rehabil; 2021.

8.Petrofsky J, Laymon M,Lee H. The effect of transcutaneous electrical nerve stimulation and low-level continuous heat on non-specific low back pain: a randomized controlled trial. Gazzetta Medica Italiana; 2020.

9.Jamison RN, Wan L,Edwards RR,Mei A,Ross EL. Outcome of a High-Frequency Transcutaneous Electrical Nerve Stimulator (hfTENS) Device for Low Back Pain: A Randomized Controlled Trial. Pain Pract; 2019.

10.Alizadeh MH, Ahmadizad S. A Comparison of Exercise Therapy and Transcutaneous Electrical Nerve Stimulation for the Treatment of Chronic Low Back Pain. World Journal of Sport Sciences; 2009.

**Online Resource 6.** GRADE Summary of Findings tables

Table 1: ***What are the benefits and harms of TENS in the management of community-dwelling adults (including older adults aged ≥60 years) with chronic primary low back pain (with or without leg pain) compared with sham?***

| Outcomes | **Anticipated absolute effects^*^** (95% CI) | | Relative effect (95% CI) | № of participants (trials) | Certainty of the evidence (GRADE) | Comments |
| --- | --- | --- | --- | --- | --- | --- |
|  | **Risk with sham** | **Risk with TENS** |  |  |  |  |
| **ALL ADULTS** | | | | | | |
| **Pain**  0 to 10; 0 = no pain follow-up: closest to 2 weeks | The mean pain was **0.95 to 9.67** | MD **0.9 lower** (1.54 lower to 0.26 lower) | - | 467 (9 RCTs)^a^ | ⨁◯◯◯ Very low^1,2,3,4,5,6,7,8,b,c,d,e^ | TENS reduces pain more than sham in the immediate term (may not be a clinically important amount). |
| **Pain**  0 to 10; 0 = no pain follow-up: closest to 3 months | The mean pain was **1.9 to 5.0** | MD **0.4 lower** (2.21 lower to 1.41 higher) | - | 117 (2 RCTs)^ae^ | ⨁◯◯◯ Very low^5,8,af,ag,q,t^ | TENS does not reduce pain more than sham in the short term. |
| **Function**  follow-up: closest to 2 weeks | The mean function was **4.09** (RMDQ, 0-24) **to 52.91** (ODI, 0-100) | SMD **0.96 SD lower** (3.2 lower to 1.28 higher) | - | 169 (4 RCTs)^ai^ | ⨁◯◯◯ Very low^2,5,7,10,aj,ak,b,q^ | TENS does not improve function more than sham in the immediate term. |
| **Function** 0 to 50; 0 = no disability follow-up: closest to 3 months | The mean function was **15.8 to 28.3** | MD **0.24 lower** (4.3 lower to 3.81 higher) | - | 117 (2 RCTs)^ae^ | ⨁◯◯◯ Very low^5,8,af,an,ao,q^ | TENS does not improve function more than sham in the short term. |
| **Health-related quality of life**  (physical component) 0 to 100; 0 = poor QofL follow-up: closest to 2 weeks | The mean health-related quality of life was **59.58 to 65.90** | MD **3.21 higher** (21.17 lower to 27.59 higher) | - | 64 (2 RCTs)^ai^ | ⨁◯◯◯ Very low^2,7,an,aq,b,q^ | TENS does not improve health-related quality of life (physical component) more than sham in the immediate term. |
| **Health-related quality of life**  mental component) 0 to 100; 0 = poor QofL follow-up: closest to 2 weeks | The mean health-related quality of life was **58.66 to 81.81** | MD **3.57 higher** (30.06 lower to 37.2 higher) | - | 64 (2 RCTs)^ai^ | ⨁◯◯◯ Very low^2,7,as,at,b,h^ | TENS does not improve health-related quality of life (mental component) more than sham in the immediate term. |
| **Depression**: 0 to 63; 0 = no depression follow-up: closest to 3 months | The mean depression was **9.3** | MD **3.04 higher** (19.15 lower to 25.22 higher) | - | 73 (1 RCT)^ae^ | ⨁◯◯◯ Very low^8,av,g,h,w^ | TENS does not reduce depression more than sham in the short term. |
| **Other psychological functioning** (fear avoidance, catastrophizing, anxiety, self-efficacy) | -- | **--** | - | (0 RCTs) | - | TENS does not reduce pain more than sham in the short term. |
| **Adverse events/harms** | Authors reported that no TENS-associated adverse events developed in any participants. | | - | 73 (1 RCT)^ae^ | ⨁◯◯◯ Very low^8,av,g,h,w^ | TENS does not contribute to adverse events/harms more than sham. |
| **Social participation** | -- | **--** | - | (0 RCTs) | - |  |
| **OLDER ADULTS (aged 60 years or more)** | | | | | | |
| **Pain**  0 to 10; 0 = no pain follow-up: closest to 2 weeks | The mean pain was **4.5** | MD **0.13 higher** (9.8 lower to 10.06 higher) | - | 28 (1 RCT)^r^ | ⨁◯◯◯ Very low^6,b,g,h,t^ | TENS does not reduce pain more than sham in the immediate term. |
| Function | -- | **--** | - | (0 RCTs) | - |  |
| Health-related quality of life | -- | **--** | - | (0 RCTs) | - |  |
| **Psychological functioning** (depression, fear avoidance, catastrophizing, anxiety, self-efficacy) | -- | **--**) | - | (0 RCTs) | - |  |
| **Social participation** | -- | **--** | - | (0 RCTs) | - |  |
| **Change in use of medications** | -- | **--** | - | (0 RCTs) | - |  |
| **Falls** | -- | **--** | - | (0 RCTs) | - |  |
| **Adverse events/harms** | -- | **--** | - | (0 RCTs) | - |  |
| ***The risk in the intervention group** (and its 95% confidence interval) is based on the assumed risk in the comparison group and the **relative effect** of the intervention (and its 95% CI).  **CI:** confidence interval; **MD:** mean difference; **SMD:** standardised mean difference | | | | | | |
| **GRADE Working Group grades of evidence** **High certainty:** we are very confident that the true effect lies close to that of the estimate of the effect. **Moderate certainty:** we are moderately confident in the effect estimate: the true effect is likely to be close to the estimate of the effect, but there is a possibility that it is substantially different. **Low certainty:** our confidence in the effect estimate is limited: the true effect may be substantially different from the estimate of the effect. **Very low certainty:** we have very little confidence in the effect estimate: the true effect is likely to be substantially different from the estimate of effect. | | | | | | |

#### Explanations

a. Four trials had 2 arms: Dias 2021 (TENS (GT100Hz) vs. sham, TENS (GT2Hz) vs. sham), Topuz 2004 (conventional TENS vs. sham, low-frequency TENS vs. sham), Yaksi 2021 (burst TENS vs. sham, conventional TENS vs. sham) and Shimoji 2007 (TENS bidirectional modulated sine wave vs. sham, TENS conventional bidirectional pulsed wave vs. sham). For each of these 4 trials we included both arms in meta-analysis and split the comparison groups in half. One trial reporting only p-values was not included in meta-analysis (Bloodworth 2004); results were reported narratively.

b. Risk of bias: We downgraded twice. Most or all of the trials were rated as overall high risk of bias.

c. Inconsistency: We downgraded once. There is similarity in some of the point estimates with overlapping confidence intervals. Statistical heterogeneity is between 50% and 90% (i.e., I2 = 77%); this could not be explained due to small subgroups and may represent substantial heterogeneity.

d. Indirectness: We did not downgrade. Multiple trials are included from different countries both high- and lower-middle income.

e. Imprecision: We downgraded once due to small sample size (OIS would have not been achieved). The point estimate did not reach the pre-specified threshold for what may be considered appreciable benefit (MD = -1); the confidence interval does not cross the null, but the lower boundary crosses the threshold for what may be considered appreciable benefit (MD = -1).

f. Risk of bias: We downgraded twice due to unclear items related to selection and reporting bias.

g. Inconsistency: We did not downgrade; however, there are no additional trials with which to compare these findings.

h. Indirectness: We downgraded once. This is a single trial from a single centre (high or upper-middle income country).

i. Imprecision: We downgraded twice due to low sample size (the OIS would not have been reached).

j. Imprecision: We downgraded once. The sample size is small (OIS would not have been achieved). The point estimate did not reach the pre-specified threshold for what may be considered appreciable benefit (MD = -1); the confidence interval crossed the null but not the thresholds for what may be considered appreciable benefit (-1) or harm (+1).

k. Inconsistency: We downgraded once. There is similarity in some of the point estimates with overlapping confidence intervals. Statistical heterogeneity is between 50% and 90% (i.e., I2 = 73%); this could not be explained due to small subgroups and may represent substantial heterogeneity.

l. Imprecision: We downgraded once due to small sample size (the OIS would not have been reached). The point estimate reached the pre-specified threshold for what may be considered appreciable benefit (MD = -1). The confidence interval did not cross the null.

m. Inconsistency: We downgraded once. There is similarity in the majority of the point estimates with overlapping confidence intervals. Statistical heterogeneity is between 50% and 90% (i.e., I2 = 74%); this could not be explained due to small subgroups and may represent substantial heterogeneity.

n. Imprecision: We downgraded once. The sample size is small (OIS would not have been achieved). The point estimate did not reach the pre-specified threshold for what may be considered appreciable benefit (MD = -1). The confidence interval crossed the null and the lower boundary crossed the threshold for what may be considered appreciable benefit (MD = -1).

o. These trials had 2 arms each: Dias 2021 (TENS (GT100Hz) vs. sham, TENS (GT2Hz) vs. sham), Topuz 2004 (conventional TENS vs. sham, low-frequency TENS vs. sham).

p. Inconsistency: We did not downgrade. The point estimates are similar with overlapping confidence intervals; statistical heterogeneity is between 0% and 40%, which might not be important (i.e., I2 = 0%).

q. Indirectness: We did not downgrade because the trials were conducted in different countries (high or upper-middle income).

r. Shimoji 2007 included 2 arms (TENS bidirectional modulated sine wave vs. sham, TENS conventional bidirectional pulsed wave vs. sham). Both were included in meta-analysis and the comparison group was split in half.

s. Inconsistency: We downgraded twice. The point estimates differ with some overlap in confidence intervals. Statistical heterogeneity is between 50% and 90% (i.e., I2 = 72%); this could not be explained due to small subgroups and may represent substantial heterogeneity.

t. Imprecision: We downgraded twice. The sample size is small (OIS would have not been achieved). The point estimate did not reach the pre-specified threshold for what may be considered clinically important (MD = -1); the confidence interval crosses the null with the lower and upper boundaries crossing the pre-specified thresholds of appreciable benefit (MD = -1) and harm (MD = +1).

u. Four trials had 2 arms: Dias 2021 (TENS (GT100Hz) vs. sham, TENS (GT2Hz) vs. sham), Topuz 2004 (conventional TENS vs. sham, low-frequency TENS vs. sham), Yaksi 2021 (burst TENS vs. sham, conventional TENS vs. sham), and Shimoji 2007. For each of these 4 trials we included both arms in meta-analysis and split the comparison groups in half.

v. Inconsistency: We downgraded once. There is similarity in some of the point estimates with overlapping confidence intervals. Statistical heterogeneity is between 50% and 90% (i.e., I2 = 78%); this could not be explained due to small subgroups and may represent substantial heterogeneity.

w. Risk of bias: We downgraded once due to the potential for selection and performance bias.

x. Indirectness: We downgraded once. This is a single trial from a single centre (low or lower-middle income country).

y. Two trials included 2 arms (Dias 2021: (TENS (GT100Hz) vs. sham, TENS (GT2Hz) vs. sham); and Shimoji 2007. All arms were included in the meta-analyses by splitting the comparison groups in half.

z. Inconsistency: We downgraded twice. Some estimates differ in direction. Statistical heterogeneity is between 50% and 90% (i.e., I2 = 64%); this could not be explained due to small subgroups and may represent substantial heterogeneity.

aa. Two trials had 2 arms each (Topuz 2004: conventional TENS vs. sham, low-frequency TENS vs. sham; Yaksi 2021: burst TENS vs. sham, conventional TENS vs. sham). For each of these 2 trials we included both arms in meta-analysis and split the comparison groups in half.

ab. Inconsistency: We downgraded once. There is similarity in some of the point estimates with overlapping confidence intervals. Statistical heterogeneity is between 50% and 90% (i.e., I2 = 84%); this could not be explained due to small subgroups and may represent substantial heterogeneity.

ac. Risk of bias: We downgraded once. Items were rated as unclear in the selection, performance and reporting domains.

ad. Inconsistency: We downgraded once. There is similarity in some of the point estimates with overlapping confidence intervals. Statistical heterogeneity is between 50% and 90% (i.e., I2 = 70%); this could not be explained due to small subgroups and may represent substantial heterogeneity.

ae. Yaksi 2021 had 2 arms (burst TENS vs. sham, conventional TENS vs. sham); both arms were included in the meta-analysis with the comparison group split in half.

af. Risk of bias: We downgraded twice due to the potential for selection, performance and reporting biases.

ag. Inconsistency: We downgraded once. There is similarity in the point estimates with overlapping confidence intervals. Statistical heterogeneity is between 30% and 60% (i.e., I2 = 50%); this could not be explained due to small subgroups and may represent moderate heterogeneity.

ah. Imprecision: We downgraded twice. The sample size is small (OIS would have not been achieved). The point estimate reached the pre-specified threshold for what may be considered clinically important (MD = -1); the confidence interval crosses the null.

ai. Topuz 2004 had 2 arms(conventional TENS vs. sham, low-frequency TENS vs. sham); both were included in the meta-analysis and the comparison group was split in half.

aj. Inconsistency: We downgraded twice. The results are in different directions with some non-overlapping confidence intervals. Statistical heterogeneity is between 75% and 100% (i.e., I2 = 92%); this could not be explained due to small subgroups and may represent considerable heterogeneity.

ak. Imprecision: We downgraded twice. The sample size is small (OIS would have not been achieved). The point estimate reached the pre-specified threshold for what may be considered clinically important (MD = -0.2); the confidence interval crosses the null.

al. Imprecision: We downgraded twice. The sample size is small (OIS would have not been achieved). The point estimate did not reach the pre-specified threshold for what may be considered clinically important (SMD = -0.2); the confidence interval crosses the null with the lower and upper boundaries crossing the pre-specified thresholds of appreciable benefit (MD = -0.2) and harm (MD = +0.2).

am. Imprecision: We downgraded once due to low sample size (the OIS would not have been reached).

an. Inconsistency: We downgraded once. The point estimates are in different directions with overlapping confidence intervals; statistical heterogeneity is between 0% and 40%, which might not be important (i.e., I2 = 0%).

ao. Imprecision: We downgraded once. The sample size is small (OIS would not have been achieved). The point estimate did not reach the pre-specified threshold for what may be considered appreciable benefit (MD = -5); the confidence interval crossed the null but not the thresholds for what may be considered appreciable benefit (-5) or harm (+5).

ap. Imprecision: We downgraded once. The sample size is small (OIS would not have been achieved). The point estimate did not reach the pre-specified threshold for what may be considered appreciable benefit (MD = -5). The confidence interval crossed the null; the lower boundary crossed the threshold for what may be considered appreciable benefit (-5).

aq. Imprecision: We downgraded twice. The sample size is small (OIS would have not been achieved). The point estimate did not reach the pre-specified threshold for what may be considered clinically important (MD = +10); the confidence interval crosses the null with the lower and upper boundaries crossing the pre-specified thresholds of appreciable benefit (MD = +10) and harm (MD = -10).

ar. Imprecision: We downgraded twice. The sample size is small (OIS would have not been achieved). The point estimate reached the pre-specified threshold for what may be considered clinically important favouring the comparison (MD = -10); the confidence interval crossed the null.

as. Imprecision: We downgraded once. The sample size is small (OIS would not have been achieved). The point estimate did not reach the pre-specified threshold for what may be considered appreciable benefit (MD = +10); the confidence interval crossed the null.

at. Inconsistency: We downgraded twice. The point estimates differ in direction and the confidence intervals do not overlap. Statistical heterogeneity is between 50% and 90% (i.e., I2 = 87%); this could not be explained due to small subgroups and may represent substantial heterogeneity.

au. Imprecision: We downgraded once. The sample size was small (OIS would not have been reached). The pointe estimate reached the threshold for what may be considered clinically important favouring the comparison (MD = -10); the confidence interval did not cross the null.

av. Imprecision: We downgraded twice. The sample size is small (OIS would have not been achieved). The point estimate did not reach the pre-specified threshold for what may be considered clinically important (MD = -6.3). The confidence interval crosses the null with the lower and upper boundaries crossing the pre-specified thresholds of appreciable benefit (MD = -6.3) and harm (MD = +6.3).

#### References

1.Cheing GL, Hui-Chan CW. Transcutaneous electrical nerve stimulation: nonparallel antinociceptive effects on chronic clinical pain and acute experimental pain. Arch Phys Med Rehabil; 1999.

2.de Alencar Caldas VV, Maciel DG, Cerqueira MS, et al. Effect of Pain Education, Cryotherapy, and Transcutaneous Electrical Nerve Stimulation on the Pain, Functional Capacity, and Quality of Life in Patients With Nonspecific Chronic Low Back Pain: A Single-Blind Randomized Controlled Trial. Am J Phys Med Rehabil; 2021.

3.Dias LV, Cordeiro MA, Schmidt de Sales R, et al. Immediate analgesic effect of transcutaneous electrical nerve stimulation (TENS) and interferential current (IFC) on chronic low back pain: Randomised placebo-controlled trial. Journal of Bodywork & Movement Therapies; 2021.

4.Ezema CI, Onyeso OK,Nna EO,et al. Transcutaneous electrical nerve stimulation effects on pain-intensity and endogenous opioids levels among chronic low-back pain patients: A randomised controlled trial. J Back Musculoskelet Rehabil; 2022.

5.Kofotolis ND, Vlachopoulos SP,Kellis E. Sequentially allocated clinical trial of rhythmic stabilization exercises and TENS in women with chronic low back pain. Clin Rehabil; 2008.

6.Shimoji K, Takahashi N,Nishio Y,Koyanagi M,Aida S. Pain relief by transcutaneous electric nerve stimulation with bidirectional modulated sine waves in patients with chronic back pain: a randomized, double-blind, sham-controlled trial. Neuromodulation; 2007.

7.Topuz O, Ozfidan E,Ozgen M,Ardic F. Efficacy of transcutaneous electrical nerve stimulation and percutaneous neuromodulation therapy in chronic low back pain. Journal of Back and Musculoskeletal Rehabilitation; 2004.

8.Yaksi E, Ketenci A, Baslo MB, Orhan EK. Does transcutaneous electrical nerve stimulation affect pain, neuropathic pain, and sympathetic skin responses in the treatment of chronic low back pain? A randomized, placebo-controlled trial. Korean J Pain; 2021.

9.Bloodworth DM, Nguyen BN,Garver W,et al. Comparison of stochastic vs. conventional transcutaneous electrical stimulation for pain modulation in patients with electromyographically documented radiculopathy. Am J Phys Med Rehabil; 2004.

10.Kibar S, Konak HE,Ay S,Doganay Erdogan B,Evcik D. The effectiveness of combined transcutaneous electrical nerve stimulation and interferential current therapy on chronic low back pain: a randomized, double-blind, sham-controlled trial. Fiziksel Tip ve Rehabilitasyon Bilimleri Dergisi [Journal of Physical Medicine and Rehabilitation Sciences]; 2020.

Table 2: ***What are the benefits and harms of TENS in the management of community-dwelling adults (including older adults aged ≥60 years) with chronic primary low back pain (with or without leg pain) compared to no treatment or treatments where the effect of TENS could be isolated?***

| Outcomes | **Anticipated absolute effects^*^** (95% CI) | | Relative effect (95% CI) | № of participants (trials) | Certainty of the evidence (GRADE) | Comments |
| --- | --- | --- | --- | --- | --- | --- |
|  | **Risk with no treatment** | **Risk with TENS** |  |  |  |  |
| **ALL ADULTS** | | | | | | |
| **Pain**  0 to 10; 0 = no pain follow-up: closest to 2 weeks | The mean pain was **1.21 to 5.09** | MD **0.19 lower** (0.51 lower to 0.14 higher) | - | 338 (8 RCTs) | ⨁◯◯◯ Very low^1,2,3,4,5,6,7,8,a,b,c,d,e^ | TENS does not reduce pain more than no treatment in the immediate term. |
| **Pain**  0 to 10; 0 = no pain follow-up: closest to 3 months | The mean pain was **1.2 to 5.9** | MD **0.98 lower** (16.83 lower to 14.88 higher) | - | 104 (2 RCTs) | ⨁◯◯◯ Very low^6,9,l,m,t,u,v^ | TENS does not reduce pain more than no treatment in the short term. |
| **Function**  follow-up: closest to 2 weeks | The mean function was **4** (RMDQ, 0-24) **to 22.07** (ODI, 0-100) | SMD **0.32 SD lower** (0.71 lower to 0.07 higher) | - | 199 (6 RCTs) | ⨁◯◯◯ Very low^1,2,3,4,7,10,b,d,o,x,y^ | TENS does not improve function more than no treatment in the intermediate term. |
| **Function**  follow-up: closest to 3 months | The mean function was **9.9** (ODI, 0-50) **to 34.9** (PDI, 0-70) | SMD **1.05 SD higher** (18.51 lower to 20.61 higher) | - | 104 (2 RCTs) | ⨁◯◯◯ Very low^6,9,aa,ak,b,h^ | TENS does not improve function more than no treatment in the short term. |
| **Health-related quality of life**  (physical component) 0 to 100; 0 = poor QofL follow-up: closest to 2 weeks | The mean health-related quality of life was **36.36** | MD **6.82 lower** (27.06 lower to 13.42 higher) | - | 22 (1 RCT) | ⨁◯◯◯ Very low^7,al,b,g,h^ | TENS does not improve health-related quality of life (physical component) more than no treatment in the immediate term. |
| **Health-related quality of life**  mental component) 0 to 100; 0 = poor QofL follow-up: closest to 2 weeks | The mean health-related quality of life was **78.18** | MD **2.91 lower** (10.25 lower to 4.43 higher) | - | 22 (1 RCT) | ⨁◯◯◯ Very low^7,am,b,g,h^ | TENS does not improve health-related quality of life (mental component) more than no treatment in the immediate term. |
| **Depression**: 0 to 21; 0 = no depression follow-up: closest to 3 months | The mean depression was **16.6** | MD **1.4 lower** (5.57 lower to 2.77 higher) | - | 60 (1 RCT) | -^9,an,g,h,t^ | TENS does not reduce depression more than no treatment in the short term. |
| **Catastrophizing**  0 to 52; 0 = no catastrophizing follow-up: closest to 3 months | The mean catastrophizing was **25.1** | MD **11.2 lower** (17.88 lower to 4.52 lower) | - | 60 (1 RCT) | ⨁◯◯◯ Very low^9,g,h,t,w^ | TENS reduces catastrophizing more than no treatment in the short term. |
| **Other psychological functioning** (fear avoidance, anxiety, self-efficacy) | The mean fear avoidance was **0** | **0**  (0 to 0 ) | - | (0 RCTs) | - |  |
| **Social participation** | The mean social participation was **0** | **0**  (0 to 0 ) | - | (0 RCTs) | - |  |
| **Adverse events/harms** | Authors reported that none of the participants reported experiencing any long-term adverse events from using high-frequency TENS. | |  | (1 RCT) | ⨁◯◯◯ Very low^g,h,t,w^ | TENS does not contribute to more adverse events/harms compared to sham. |
| **OLDER ADULTS (aged ≥60 years)** | | | | | | |
| **Pain** | The mean pain was **0** | **0**  (0 to 0 ) | - | (0 RCTs) | - |  |
| **Function** | The mean function was **0** | **0**  (0 to 0 ) | - | (0 RCT) | - |  |
| **Health-related quality of life** | The mean health-related quality of life was **0** | **0**  (0 to 0 ) | - | (0 RCTs) | - |  |
| **Psychological functioning** (depression, fear avoidance, catastrophizing, anxiety, self-efficacy) | The mean depression was **0** | **0**  (0 to 0 ) | - | (0 RCTs) | - |  |
| **Social participation** | The mean social participation was **0** | **0**  (0 to 0 ) | - | (0 RCTs) | - |  |
| **Adverse events** | The mean adverse events were **0** | **0**  (0 to 0 ) | - | (0 RCTs) | - |  |
| **Change in use of medications** | The mean change in use of medications was **0** | **0**  (0 to 0 ) | - | (0 RCTs) | - |  |
| **Falls** | The mean falls was **0** | **0**  (0 to 0 ) | - | (0 RCTs) | - |  |
| ***The risk in the intervention group** (and its 95% confidence interval) is based on the assumed risk in the comparison group and the **relative effect** of the intervention (and its 95% CI).  **CI:** confidence interval; **MD:** mean difference; **SMD:** standardised mean difference | | | | | | |
| **GRADE Working Group grades of evidence** **High certainty:** we are very confident that the true effect lies close to that of the estimate of the effect. **Moderate certainty:** we are moderately confident in the effect estimate: the true effect is likely to be close to the estimate of the effect, but there is a possibility that it is substantially different. **Low certainty:** our confidence in the effect estimate is limited: the true effect may be substantially different from the estimate of the effect. **Very low certainty:** we have very little confidence in the effect estimate: the true effect is likely to be substantially different from the estimate of effect. | | | | | | |

#### Explanations

a. Elserty 2016 included 2 arms (fixed pulse TENS + exercise vs. exercise; adjusted pulse TENS + exercise vs. exercise). Both were included in meta-analysis by splitting the comparison group number in half. Petrofsky 2020 included 4 arms (Continuous TENS + spent sham heat vs. spent sham heat; continuous TENS + LLCH (low-level continuous heat) vs. LLCH; TENS last 15 min + LLCH vs. LLCH; TENS last 15 min + spent sham heat vs. spent sham heat). All were included in meta-analysis by splitting the comparison group numbers accordingly.

b. Risk of bias: We downgraded twice. Most or all of the trials were rated as overall high risk of bias.

c. Inconsistency: We did not downgrade. The point estimates are similar with overlapping confidence intervals; statistical heterogeneity is between 0% and 40%, which might not be important (i.e., I2 = 6%).

d. Indirectness: We did not downgrade. Trials are included from different countries both high- and lower-middle income.

e. Imprecision: We downgraded once. The sample size is small (OIS would not have been achieved). The point estimate did not reach the pre-specified threshold for what may be considered appreciable benefit (MD = -1). The confidence interval crossed the null but not the thresholds for what may be considered appreciable benefit (-1) or harm (+1).

f. Imprecision: We downgraded once. The sample size was small (OIS would not have been reached). The point estimate did not reach the pre-specified threshold for what may be considered clinically important (MD = -1); the confidence interval did not cross the null.

g. Inconsistency: We did not downgrade; however, there are no additional trials with which to compare these findings.

h. Indirectness: We downgraded once. This is a single trial from a single centre (high or upper-middle income country).

i. Inconsistency: We did not downgrade. The point estimates are similar with overlapping confidence intervals; statistical heterogeneity is between 0% and 40%, which might not be important (i.e., I2 = 0%).

j. Imprecision: We downgraded once. The sample size was small (OIS would not have been reached). The point estimate did not reach the threshold for what may be considered clinically important (MD = -1); the confidence interval did not cross the null.

k. Inconsistency: We downgraded once. The point estimates are close with some overlap in the confidence intervals. Statistical heterogeneity is between 50% and 90% (i.e., I2 = 65%). This could not be explained due to small subgroups and may represent substantial heterogeneity.

l. Indirectness: We did not downgrade because the trials were conducted in different countries (high or upper-middle income).

m. Imprecision: We downgraded twice. The sample size is small (OIS would have not been achieved). The point estimate did not reach the pre-specified threshold for what may be considered clinically important (MD = -1); the confidence interval crosses the null with the lower and upper boundaries crossing the pre-specified thresholds of appreciable benefit (MD = -1) and harm (MD = +1).

n. Inconsistency: We did not downgrade. The point estimates are similar with overlapping confidence intervals; statistical heterogeneity is between 0% and 40%, which might not be important (i.e., I2 = 10%).

o. Elserty 2016 included 2 arms (fixed pulse TENS + exercise vs. exercise; adjusted pulse TENS + exercise vs. exercise). Both were included in meta-analysis by splitting the comparison group number in half.

p. Inconsistency: We did not downgrade. The point estimates differ in direction, but the confidence intervals overlap; statistical heterogeneity is between 0% and 40%, which might not be important (i.e., I2 = 4%).

q. Indirectness: We did not downgrade because the trials were conducted in different countries (low or lower-middle income).

r. Inconsistency: We did not downgrade. The point estimates are similar with overlapping confidence intervals; statistical heterogeneity is between 30% and 60%, which may represent moderate heterogeneity (i.e., I2 = 48%).

s. Depaoli Lemos 2021 used 4 TENS sessions; Petrofsky 2020 used a single TENS session.

t. Risk of bias: We downgraded once due to the potential for selection, performance and other biases.

u. Kofotolis and Jamison: Participants had 20-90 treatment sessions.

v. Inconsistency: We downgraded twice. The point estimates were in different directions with little to no overlap in confidence intervals. Statistical heterogeneity is between 75% and 100% (i.e., I2 = 94%). This could not be explained due to small subgroups and may represent considerable heterogeneity.

w. Imprecision: We downgraded once due to small sample size (the OIS would not have been reached).

x. Inconsistency: We did not downgrade. Most of the point estimates are similar with overlapping confidence intervals; statistical heterogeneity is between 0% and 40%, which might not be important (i.e., I2 = 28%).

y. Imprecision: We downgraded once due to small sample size (the OIS would not have been reached). The point estimate reached the pre-specified threshold for what may be considered appreciable benefit (SMD = -0.2). The confidence interval crossed the null.

z. Indirectness: We downgraded once. This is a single trial from (low or lower-middle income country).

aa. Imprecision: We downgraded twice. The sample size is small (OIS would have not been achieved). The point estimate reached the pre-specified threshold for what may be considered clinically important (SMD = -0.2); the confidence interval crosses the null.

ab. Inconsistency: We did not downgrade. Most of the point estimates are similar with overlapping confidence intervals; statistical heterogeneity is between 0% and 40%, which might not be important (i.e., I2 = 39%).

ac. Imprecision: We downgraded once. The sample size is small (OIS would have not been achieved). The point estimate did not reach the pre-specified threshold for what may be considered clinically important (SMD = -0.2). The confidence interval crossed the null, but the upper boundary did not cross the pre-specified threshold for what may be considered appreciable harm (SMD = +0.2).

ad. Imprecision: We downgraded twice. The sample size is small (OIS would have not been achieved). The point estimate did not reach the pre-specified threshold for what may be considered clinically important (SMD = -0.2); the confidence interval crosses the null with the lower and upper boundaries crossing the pre-specified thresholds for what may be considered appreciable benefit (SMD = -0.2) and harm (SMD = +0.2).

ae. Imprecision: We downgraded once due to small sample size (the OIS would not have been reached). The point estimate did not reach the pre-specified threshold for what may be considered appreciable benefit (SMD = -0.2). The confidence interval crossed the null.

af. Inconsistency: We downgraded once. There was some difference in magnitude and direction of the point estimates, but there was some overlap in confidence intervals. Statistical heterogeneity is between 50% and 90% (i.e., I2 = 69%). This could not be explained due to small subgroups and may represent substantial heterogeneity.

ag. We did not downgrade because the trials were conducted in different countries.

ah. Inconsistency: We did not downgrade. Most of the point estimates are similar with overlapping confidence intervals; statistical heterogeneity is between 0% and 40%, which might not be important (i.e., I2 = 39%).

ai. Imprecision: We downgraded once due to small sample size (the OIS would not have been reached). The point estimate reached the pre-specified threshold for what may be considered appreciable benefit (SMD = -0.2). The confidence interval crossed the null, but the upper boundary did not cross the pre-specified threshold for what may be considered appreciable harm (SMD = +0.2).

aj. Depaoli Lemos 2021 used 4 TENS sessions.

ak. Inconsistency: We downgraded twice. The point estimates were in different directions with little to no overlap in confidence intervals. Statistical heterogeneity is between 75% and 100% (i.e., I2 = 97%). This could not be explained due to small subgroups and may represent considerable heterogeneity.

al. Imprecision: We downgraded twice due to small sample size (the OIS would not have been reached). The point estimate did not reach the pre-specified threshold for what may be considered appreciable harm (-10). The confidence interval crossed the null with the boundaries crossing the pre-specified thresholds for what may be considered appreciable harm (-10) and benefit (+10).

am. Imprecision: We downgraded once due to small sample size (the OIS would not have been reached). The point estimate did not reach the pre-specified threshold for what may be considered appreciable harm (MD = -10). The confidence interval crossed the null.

an. Imprecision: We downgraded twice due to small sample size (the OIS would not have been reached). The point estimate did not reach the pre-specified threshold for what may be considered appreciable benefit (MD = -2.1). The confidence interval crossed the null with the boundaries crossing the pre-specified thresholds for what may be considered appreciable benefit (-2.1) or harm (+2.1).

#### References

1.Depaoli Lemos VJ, Selau RC,Blos C,Baptista Dohnert M,Boff Daitx R,de Almeida Brito V. Electroacupuncture and Transcutaneous Electrical Nerve Stimulation in Chronic Nonspecific Low Back Pain: a Blind Randomized Clinical Trial. Muscles, ligaments & tendons journal; 2021.

2.Elserty N, Kattabei O,Elhafez H.. Effect of Fixed Versus Adjusted Transcutaneous Electrical Nerve Stimulation Amplitude on Chronic Mechanical Low Back Pain. J Altern Complement Med; 2016.

3.Tella BA, Oghumu SN,Gbiri CAO. Efficacy of transcutaneous electrical nerve stimulation and interferential current on tactile acuity of individuals with nonspecific chronic low back pain. Neuromodulation; 2021.

4.Kibar S, Konak HE,Ay S,Doganay Erdogan B,Evcik D. The effectiveness of combined transcutaneous electrical nerve stimulation and interferential current therapy on chronic low back pain: a randomized, double-blind, sham-controlled trial. Fiziksel Tip ve Rehabilitasyon Bilimleri Dergisi [Journal of Physical Medicine and Rehabilitation Sciences]; 2020.

5.Shimoji K, Takahashi N,Nishio Y,Koyanagi M,Aida S. Pain relief by transcutaneous electric nerve stimulation with bidirectional modulated sine waves in patients with chronic back pain: a randomized, double-blind, sham-controlled trial. Neuromodulation; 2007.

6.Kofotolis ND, Vlachopoulos SP,Kellis E. Sequentially allocated clinical trial of rhythmic stabilization exercises and TENS in women with chronic low back pain. Clin Rehabil; 2008.

7.de Alencar Caldas VV, Maciel DG, Cerqueira MS, et al. Effect of Pain Education, Cryotherapy, and Transcutaneous Electrical Nerve Stimulation on the Pain, Functional Capacity, and Quality of Life in Patients With Nonspecific Chronic Low Back Pain: A Single-Blind Randomized Controlled Trial. Am J Phys Med Rehabil; 2021.

8.Petrofsky J, Laymon M,Lee H. The effect of transcutaneous electrical nerve stimulation and low-level continuous heat on non-specific low back pain: a randomized controlled trial. Gazzetta Medica Italiana; 2020.

9.Jamison RN, Wan L,Edwards RR,Mei A,Ross EL. Outcome of a High-Frequency Transcutaneous Electrical Nerve Stimulator (hfTENS) Device for Low Back Pain: A Randomized Controlled Trial. Pain Pract; 2019.

10.Alizadeh MH, Ahmadizad S. A Comparison of Exercise Therapy and Transcutaneous Electrical Nerve Stimulation for the Treatment of Chronic Low Back Pain. World Journal of Sport Sciences; 2009.

**References**

1. Alizadeh, M.H. and S. Ahmadizad, *A comparison of exercise therapy and transcutaneous electrical nerve stimulation for the treatment of chronic low back pain.* World Journal of Sport Sciences, 2009. **2**(1): p. 43-47.

2. Bloodworth, D.M., et al., *Comparison of stochastic vs. conventional transcutaneous electrical stimulation for pain modulation in patients with electromyographically documented radiculopathy.* Am J Phys Med Rehabil, 2004. **83**(8): p. 584-91.

3. Cheing, G.L. and C.W. Hui-Chan, *Transcutaneous electrical nerve stimulation: nonparallel antinociceptive effects on chronic clinical pain and acute experimental pain.* Arch Phys Med Rehabil, 1999. **80**(3): p. 305-12.

4. de Alencar Caldas, V.V., et al., *Effect of pain education, cryotherapy, and transcutaneous electrical nerve stimulation on the pain, functional capacity, and quality of life in patients with nonspecific chronic low back pain: a single-blind randomized controlled trial.* Am J Phys Med Rehabil, 2021. **100**(3): p. 243-249.

5. Depaoli Lemos, V.J., et al., *Electroacupuncture and transcutaneous electrical nerve stimulation in chronic nonspecific low back pain: a blind randomized clinical trial.* Muscles, ligaments, tendons J, 2021. **11**(4): p. 719‐727.

6. Dias, L.V., et al., *Immediate analgesic effect of transcutaneous electrical nerve stimulation (TENS) and interferential current (IFC) on chronic low back pain: randomised placebo-controlled trial.* J Bodyw Mov Ther, 2021. **27**: p. 181-190.

7. Elserty, N., O. Kattabei, and H. Elhafez, *Effect of fixed versus adjusted transcutaneous electrical nerve stimulation amplitude on chronic mechanical low back pain.* J Altern Complement Med, 2016. **22**(7): p. 557-62.

8. Ezema, C.I., et al., *Transcutaneous electrical nerve stimulation effects on pain-intensity and endogenous opioids levels among chronic low-back pain patients: a randomised controlled trial.* J Back Musculoskelet Rehabil, 2022.

9. Jamison, R.N., et al., *Outcome of a high-frequency transcutaneous electrical nerve stimulator (hfTENS) device for low back pain: a randomized controlled trial.* Pain Pract, 2019. **19**(5): p. 466-475.

10. Kibar, S., et al., *The effectiveness of combined transcutaneous electrical nerve stimulation and interferential current therapy on chronic low back pain: a randomized, double-blind, sham-controlled study.* Fiziksel Tip ve Rehabilitasyon Bilimleri Dergisi [J Phys Med Rehabil Sci] 2020;23(1):32-40, 2020.

11. Kofotolis, N.D., S.P. Vlachopoulos, and E. Kellis, *Sequentially allocated clinical trial of rhythmic stabilization exercises and TENS in women with chronic low back pain.* Clin Rehabil, 2008. **22**(2): p. 99-111.

12. Petrofsky, J., M. Laymon, and H. Lee, *The effect of transcutaneous electrical nerve stimulation and low-level continuous heat on non-specific low back pain: a randomized controlled trial.* Gazzetta Medica Ital, 2020. **179**(6): p. 419-27.

13. Shimoji, K., et al., *Pain relief by transcutaneous electric nerve stimulation with bidirectional modulated sine waves in patients with chronic back pain: a randomized, double-blind, sham-controlled study.* Neuromodulation, 2007. **10**(1): p. 42-51.

14. Tella, B.A., S.N. Oghumu, and C.A.O. Gbiri, *Efficacy of transcutaneous electrical nerve stimulation and interferential current on tactile acuity of individuals with nonspecific chronic low back pain.* Neuromodulation 2021 Aug 17:Epub ahead of print, 2021.

15. Topuz, O., et al., *Efficacy of transcutaneous electrical nerve stimulation and percutaneous neuromodulation therapy in chronic low back pain.* J Back Musculoskelet Rehabil, 2004. **17**: p. 127–133.

16. Yaksi, E., et al., *Does transcutaneous electrical nerve stimulation affect pain, neuropathic pain, and sympathetic skin responses in the treatment of chronic low back pain? A randomized, placebo-controlled study.* Korean J Pain, 2021. **34**(2): p. 217-228.

17. Akhmadeeva, L., et al., *The effects of transcutaneous electrical nerve stimulation (TENS) for patients with low back pain: first two randomized contolled trials in Russia with dynamic TENS devices.* J Neurol, 2014.

18. Akhmadeeva, L., N. Setchenkova, and R. Magzhanov, *Effectiveness of transcutaneous dynamic electrical nerve stimulation in low back pain: a pilot randomized controlled trial.* J Neurol Sci 2009. **Conference**: p. S320.

19. Cheing, G. and C.W.Y. Hui-Chan. *Repeated applications of transcutaneous electrical nerve stimulation (TENS) produce cumulative effects on chronic clinical pain but not acute experimental pain in chronic low back pain patients*. in *8th World Congress on the International Association for the Study of Pain* 1996.

20. Efanov, O.I., *[Clinical use of random currents].* 1984.

21. Elvir, O.L., et al., *Analgesic efficacy of TENS in chronic low back pain: effect of fixed vs variable-rate stimulation.* Anesth Analg, 2010. **110**: p. S-366.

22. Evanoff, A. and W.P. Newton, *An alternative treatment for low back pain.* J Fam Pract, 1999. **48**(6): p. 416-7.

23. Hazime, F.A., et al., *Analgesic efficacy of cerebral and peripheral electrical stimulation in chronic nonspecific low back pain: a randomized, double-blind, factorial clinical trial.* BMC Musculoskelet Disord, 2015. **16**: p. 7.

24. Hush, J., *TENS of unknown value in the treatment of chronic low back pain. Commentary.* 2006.

25. Jamison, R., et al., *Efficacy of high-frequency transcutaneous electrical nerve stimulation for chronic low back pain: does hypersensitivity matter? .* 2018.

26. Ketenci, A. and E. Yahsi, *Evaluation of the pain, neuropathic pain and sympathetic skin response to transcutaneous electrical nerve stimulation treatment in chronic mechanical low back pain patients.* Osteoporosis Int, 2017. **28**(Suppl 1): p. S127-S636.

27. Magbagbeola, J., et al. *Transcutaneous electrical nerve stimulation (TENS) for relieving low-back pain in Saudi Arabia, Eastern Province*. 1987.

28. Sodipo, J.O.A., *Transcutaneous electrical nerve stimulation (TENS) and acupuncture: comparison of therapy for low-back pain.* Pain, 1981(Supp1): p. S277.

29. Thiese, M.S., M. Hughes, and J. Biggs, *Electrical stimulation for chronic non-specific low back pain in a working-age population: a 12-week double blinded randomized controlled trial.* BMC Musculoskelet Disord, 2013. **14**: p. 117.

30. Itoh, K., et al., *A pilot study on using acupuncture and transcutaneous electrical nerve stimulation to treat chronic non-specific low back pain.* Complement Ther Clin Pract, 2009. **15**(1): p. 22-5.

31. Kaur, G., *An experimental study to see the efficacy of manual therapy and conventional therapy in low back pain.* Indian J Physiother Occup Ther, 2015. **9**(4): p. 23-27.

32. Marchand, S., et al., *Is TENS purely a placebo effect? A controlled study on chronic low back pain.* Pain, 1993. **54**(1): p. 99-106.

33. Poitras, S. and L. Brosseau, *Evidence-informed management of chronic low back pain with transcutaneous electrical nerve stimulation, interferential current, electrical muscle stimulation, ultrasound, and thermotherapy.* Spine J, 2008. **8**(1): p. 226-33.

34. Popovic, D.B., et al., *Lumbar stimulation belt for therapy of low-back pain.* Artif Organs, 2009. **33**(1): p. 54-60.

35. Rutkowski, B., T. Niedzialkowska, and J. Otto, *Electrical stimulation in chronic low-back pain.* Br J Anaesth, 1977. **49**(6): p. 629-32.

36. Akhmadeeva, L.R., et al., *[Randomized blind placebo-controlled study of the effectiveness of transcutaneous adaptive electrostimulation in the treatment of nonspecific low back pain].* Zh Nevrol Psikhiatr Im S S Korsakova, 2010. **110**(4): p. 57-62.

37. Aguilar Ferrandiz ME, et al., *Auto-targeted neurostimulation is not superior to placebo in chronic low back pain: a fourfold blind randomized clinical trial* Pain Physician, 2016. **19**: p. E707-E719.

38. Bergeron-Vezina, K., et al., *Adjusting pulse amplitude during transcutaneous electrical nerve stimulation does not provide greater hypoalgesia.* J Altern Complement Med, 2018. **24**(3): p. 262-267.

39. Buchmuller, A., et al., *Value of TENS for relief of chronic low back pain with or without radicular pain.* Eur J Pain, 2012. **16**(5): p. 656-65.

40. Deyo, R.A., et al., *A controlled trial of transcutaneous electrical nerve stimulation (TENS) and exercise for chronic low back pain.* N Engl J Med, 1990. **322**(23): p. 1627-34.

41. Hawamdeh, M., et al., *Comparison between the effectiveness of using transcutaneous electrical nerve stimulation (TENS) with back strengthening exercises and the use of back strengthening exercises only on patients with chronic low back pain: a pilot crossover double blind study.* Indian J Physiother Occup Ther, 2015. **9**(2): p. 210-214.

42. Facci, L.M., et al., *Effects of transcutaneous electrical nerve stimulation (TENS) and interferential currents (IFC) in patients with nonspecific chronic low back pain: randomized clinical trial.* Sao Paulo Med J 2011. **129**: p. 206-216.

43. Hsieh, R.L. and W.C. Lee, *One-shot percutaneous electrical nerve stimulation vs. transcutaneous electrical nerve stimulation for low back pain: comparison of therapeutic effects.* Am J Phys Med Rehabil, 2002. **81**(11): p. 838-43.

44. Hsieh, C.Y., et al., *Functional outcomes of low back pain: comparison of four treatment groups in a randomized controlled trial.* J Manipulative Physiol Ther, 1992. **15**(1): p. 4-9.

45. Jarzem, P.F., et al., *Transcutaneous electrical nerve stimulation [TENS] for chronic low back pain.* J Musculoskelet Pain 2005. **13**(2): p. 3-9.

46. Jarzem, P.F., et al., *Transcutaneous electrical nerve stimulation [TENS] for short-term treatment of low back pain–randomized double blind crossover study of sham versus conventional TENS.* J Musculoskelet Pain, 2005. **13**(2): p. 11-17.

47. Kumar, S., V.P. Sharma, and M.P.S. Negi, *Efficacy of dynamic muscular stabilization techniques (DMST) over conventional techniques in rehabilitation of chronic low back pain [with consumer summary].* J Strength Cond Res 2009. **23**(9): p. 2651-2659.

48. Leemans, L., et al., *Transcutaneous electrical nerve stimulation and heat to reduce pain in a chronic low back pain population: a randomized controlled clinical trial.* Braz J Phys Ther, 2021. **25**(1): p. 86-96.

49. Lehmann, T.R., D.W. Russell, and K.F. Spratt, *The impact of patients with nonorganic physical findings on a controlled trial of transcutaneous electrical nerve stimulation and electroacupuncture.* Spine (Phila Pa 1976), 1983. **8**(6): p. 625-34.

50. Lehmann, T.R., et al., *Efficacy of electroacupuncture and TENS in the rehabilitation of chronic low back pain patients.* Pain, 1986. **26**(3): p. 277-290.

51. Moore, S.R. and J. Shurman, *Combined neuromuscular electrical stimulation and transcutaneous electrical nerve stimulation for treatment of chronic back pain: a double-blind, repeated measures comparison.* Arch Phys Med Rehabil, 1997. **78**(1): p. 55-60.

52. Sakai, T., et al., *Multi-center randomized controlled trial of acupuncuture with electric stimulation and acupuncture-like transcutaneous electrical nerve stimulation for lumbago (in Japanese).* Journal of the Japan Society of Acupuncture Moxibustion 2002: p. 175-184.

53. Wang, L., et al., *Clinical effects of electrical stimulation therapy on lumbar disc herniation-induced sciatica and its influence on peripheral ROS level.* J Musculoskelet Neuronal Interact, 2018. **18**(3): p. 393-398.

54. Warke, K., et al., *Efficacy of transcutaneous electrical nerve stimulation (tens) for chronic low-back pain in a multiple sclerosis population: a randomized, placebo-controlled clinical trial.* Clin J Pain, 2006. **22**(9): p. 812-9.

55. Alrwaily, M., et al., *Stabilization exercises combined with neuromuscular electrical stimulation for patients with chronic low back pain: a randomized controlled trial* Braz J Phys Ther, 2019. **23**(6): p. 506-515.

56. Bordiak, F.C. and E.B. da Silva, *Electrical stimulation and core training on pain and range of motion in low back pain.* Fisioter Mov 2012. **25**(4): p. 759-66.

57. Durmus, D., et al., *Effects of electrical stimulation program on trunk muscle strength, functional capacity, quality of life, and depression in the patients with low back pain: a randomized controlled trial.* Rheumatol Int, 2009. **29**(8): p. 947-54.

58. Durmus, D., Y. Durmaz, and F. Canturk, *Effects of therapeutic ultrasound and electrical stimulation program on pain, trunk muscle strength, disability, walking performance, quality of life, and depression in patients with low back pain: a randomized-controlled trial.* Rheumatol Int, 2010. **30**(7): p. 901-10.

59. Kim, Y.K., S.Y. Cho, and K.H. Lee, *Effects of transcutaneous electrical nerve stimulation and instrument-assisted soft tissue mobilization combined treatment on chronic low back pain: a randomized controlled trial.* J Back Musculoskelet Rehabil, 2021. **34**(5): p. 895-902.

60. Kim, T.H., E.H. Kim, and H.Y. Cho, *The effects of the CORE programme on pain at rest, movement-induced and secondary pain, active range of motion, and proprioception in female office workers with chronic low back pain: a randomized controlled trial.* Clin Rehabil, 2015. **29**(7): p. 653-62.

61. Neuwersch-Sommeregger, S., et al., *[Electrical muscle stimulation in combination with heat for patients with chronic, nonspecific low back pain : a randomized, double-blind, stratified, placebo-controlled clinical trial].* Der Schmerz, 2020. **34**: p. 65-73.

62. Ozkaraoglu, D.K., D. Tarakci, and Z.C. Algun, *Comparison of two different electrotherapy methods in low back pain treatment.* J Back Musculoskelet Rehabil 2020. **33**(2): p. 193-199.

63. Pelegrini ACA, G.E., Bussolaro JM, Segatti G, de Albuquerque CE, Bertolini GRF. , *The analgesic action of Aussie current in women with non-specific chronic lumbar pain.* Int J Ther Rehabil 2019.

64. Potturi, G., et al., *Effect of dexamethasone iontophoresis combined with strong surged faradic current on piriformis syndrome - a simple randomized control clinical trial.* Indian J Physiother Occup Ther 2014. **8**(8): p. 265-271.

65. Schabrun, S.M., et al., *Targeting chronic recurrent low back pain from the top-down and the bottom-up: a combined transcranial direct current stimulation and peripheral electrical stimulation intervention.* Brain Stimul, 2014. **7**(3): p. 451-9.

66. Starkweather, A.R., et al., *Decreased low back pain intensity and differential gene expression following Calmare(R): results from a double-blinded randomized sham-controlled study.* Res Nurs Health, 2015. **38**(1): p. 29-38.

67. Thompson, J.W., S. Bower, and S.P. Tyrer, *A double blind randomised controlled clinical trial on the effect of transcutaneous spinal electroanalgesia (TSE) on low back pain.* Eur J Pain, 2008. **12**(3): p. 371-7.

68. Ebadi, S., et al., *No immediate analgesic effect of diadynamic current in patients with nonspecific low back pain in comparison to TENS.* J Bodyw Mov Ther, 2018. **22**(3): p. 693-699.

69. Ghoname, E.A., et al., *Percutaneous electrical nerve stimulation for low back pain: a randomized crossover study.* JAMA, 1999. **281**(9): p. 818-23.

70. Ghoname, E.A., et al., *Percutaneous electrical nerve stimulation: an alternative to TENS in the management of sciatica.* Pain, 1999. **83**(2): p. 193-9.

71. Grabianska, E., et al., *Porownanie dzialania przeciwbolowego pradow interferencyjnych i TENS u pacjentow z dolegliwosciami bolowymi w czesci ledzwiowo-krzyzowej kregoslupa (Comparison of the analgesic effect of interferential current (IFC) and TENS in patients with low back pain) [Polish].* Wiadomosci Lekarskie 2015;68(1):13-19, 2015.

72. Rajfur, J., et al., *Efficacy of selected electrical therapies on chronic low back pain: a comparative clinical pilot study.* Medical Science Monitor 2017 Jan 7;23:85-100, 2017.

73. Ratajczak, B., et al., *Effectiveness of diadynamic currents and transcutaneous electrical nerve stimulation in disc disease lumbar part of spine.* J Back Musculoskelet Rehabil, 2011. **24**(3): p. 155-9.

74. Tousignant-Laflamme, Y., et al., *A randomized trial to determine the duration of analgesia following a 15- and a 30-minute application of acupuncture-like TENS on patients with chronic low back pain* Physiother Theory Pract, 2017. **33**(5): p. 361-369.

75. Weizemann, C., et al., *Effect of transcutaneous electrical nerve stimulation and hypnosis on chronic low back pain.* BrJP, 2021. **4**(1): p. 26-30.

76. Ariel, E., et al., *The effects of TENS, interferential stimulation, and combined interferential stimulation and pulsed ultrasound on patients with disc herniation-induced radicular pain.* J Back Musculoskelet Rehabil, 2022. **35**(2): p. 363-371.

77. Rojhani-Shirazi, Z. and T. Rezaeian, *The effects of transcutaneous electrical nerve stimulation on postural control in patients with chronic low back pain.* J Med Life, 2015. **8**(Spec Iss 2): p. 19-27.
